# Conceptualising systems thinking and complexity modelling for circular economy quantification: A systematic review and critical analysis

**DOI:** 10.1177/0734242X251413436

**Published:** 2026-02-16

**Authors:** Soumava Boral, Leon Black, Costas A Velis

**Affiliations:** 1 School of Civil Engineering, University of Leeds, Leeds, UK; 2 Department of Civil and Environmental Engineering, Imperial College London, London, UK

**Keywords:** Circular economy, systems thinking, complexity modelling, agent-based modelling and simulation, system dynamics

## Abstract

Quantification of circular economy (CE) is essential for effective implementation, yet also fundamentally challenging, because it is inherently complex, featuring multiple interactions and system-level dynamicity. Two main approaches of systems thinking, commonly used to model complexities in intricate systems, are: system dynamics (SD), providing a top-down, macroscopic view; and agent-based modelling and simulation (ABMS), offering a bottom-up, microscopic perspective. Here we conducted a Preferred Reporting Items for Systematic Reviews and Meta-Analyses for Scoping Reviews (PRISMA-ScR) review, examining 60 studies applying SD or ABMS to CE, across sectors such as bio-based materials, construction and industrial symbiosis. Both methods capture aspects of circularity’s feedback loops and time evolution, but they are often used in isolation in the absence of integrated platforms along with concerns over computational costs. This limits their capacity to comprehensively model internal dynamics at multiple scales and provide system-wide decision support. Few studies explore the potential of combining SD and ABMS or attempt to integrate them with static tools, such as life-cycle assessment and multi-criteria decision analysis. Standardised metrics and operational holistic evaluation tools incorporating economic, environmental, technical and social sustainability aspects are missing – especially with the latter. A more unified and comprehensive systems approach to support informed decisions on circularity would improve evidence-based policymaking and empower wider industrial adoption.

## Introduction

The concept and practice of circular economy (CE)/circularity intend to go beyond the established practices of waste management and resource recovery, towards an even more sustainable management of material resources. Numerous circular economy/circularity definitions have been proposed, as summarised in detail by Kirchherr et al. (2017, 2023). For example, the United Nations Environment Programme (UNEP) described CE as *‘an economy that reduces consumption of resources and the generation of waste, and reuses and recycles waste throughout the production, distribution and consumption processes’* (United Nations Environment Programme, 2011). More recently, BS ISO 59004:2024 defined circular economy as an *‘economic system that uses a systemic approach to maintain a circular flow of resources by recovering, retaining or adding to their value, while contributing to sustainable development’* (The British Standards Institution, 2024a). An alternative earlier definition, closer to the beginning of the recent update of the circular economy notion, proposed that *‘[. . . circular economy] is restorative and regenerative by design and aims to keep products, components, and materials at their highest utility and value at all times, distinguishing between technical and biological cycles’* (Ellen MacArthur Foundation, 2013); therefore, encouraging a shift from ‘cradle-to-grave’ thinking to a ‘cradle-to-cradle’ philosophy, thus promoting the idea of maximising the positive ‘value’ associated with material, components and products (MCPs). This wording emphasises the concept of MCPs’ value regeneration and redistribution through their entire lifecycle, and multiple iterations thereof.

Nonetheless, this approach does not define the inherently multi-dimensional and therefore complex meaning of ‘value’, which usually goes beyond the monetary loss/benefits, to incorporate environmental benefits, social equity and prosperity and minimal technical performance loss (e.g. design for reuse) (Iacovidou et al., 2017a, 2017b; Millward-Hopkins et al., 2018). BS ISO 59004:2024 defines ‘value’ as *“‘gain(s) or benefit(s) from satisfying needs and expectations, in relation to the use and conservation of resources”’* (The British Standards Institution, 2024a). Such a value transforms: it is created/destroyed/transferred between places and owned by organisations, as MCPs are physically moved and transformed in the extraction of resources, materials, components, semi-finished goods and goods are manufactured, retailed, used and becoming after-use/waste and entering the waste and resource recovery/disposal part of the cycle. Measuring this value as an attempt to quantify circularity is therefore feasible only in a comparative way, within a system at which the value carried by MCPs takes specific ‘states’/‘levels’ at different points.

For example, simplified typologies on the mode of circularity have been historically narrated via ‘R’-type based frameworks (Table 1), and the definitions of the various R’s are provided in Supplemental section S.2 as per BS ISO 59004:2024 (The British Standards Institution, 2024a). They offer a simple conceptual hierarchy of circularity modalities applicable at various MCPs lifecycle stages, focusing on slowing, closing or narrowing resource flows. Similarly, quantifying the nature and degree of circularity achieved by these R-type modalities requires as a bare minimum definition of ratios between parts of a material flows system. From a whole system’s perspective, the implementation of each ‘R’ philosophy is highly time-dependent, short- and long-term impacts (i.e. time dynamicity and delays) with potential benefits (e.g. diversion from landfill) or drawbacks (e.g. insufficient recycling capabilities driving increased disposal), and it depends on stakeholders’ activities along value chains and value networks (Walzberg et al., 2022b), that is, in material flow systems.

**Table 1.** A variety of ‘R’ concept-based frameworks proposed.

Framework	‘R’- type content	References
3-Rs-based	Reduce, reuse and recycle	Brennan et al. (2015)
4-Rs-based	Reduce, reuse, recycle, recover	Yang et al. (2017)
6-Rs-based	Reduce, redesign, recover, reuse, remanufacture, recycle	Jawahir and Bradley (2016)
9-Rs-based	Refuse, reduce, reuse, repair, refurbish, remanufacture, repurpose, recycle and recover energy	Van Buren et al. (2016)
10-Rs-based	Rethink, reduce, reuse, repair, refurbish, remanufacture, repurpose, recycle, recover and refuse	Kirchherr et al. (2017)

In such material flow systems, multiple variables may cause system dynamicity: i.e., qualitative and quantitative, endogenous and exogeneous, linear or non-linear. Understanding stocks and flows of material, energy, finance, etc. is key to sufficiently describe such a CE system, where multiple stakeholders are involved along the entire value-chain, sometimes in closed loops introducing feedback in the system, each aiming to maximise its own benefits, therefore transforming the monetised or wider perceived ‘value’ of MCPs at each system point over time. As a result, such systems feature numerous complexities, and feature trade-offs among aspects of ‘value’ which would be very difficult, if at all possible, to capture/summarise solely through traditional analytical, equations-based, approaches (Sun et al., 2016).

Similarly, system boundaries (time, geographical, administrative, scale) may play a crucial role in systems thinking, and while the actions of system agents may not reveal impacts at a ‘micro’ scale, they may do so at ‘meso’ or ‘macro’ scales. For example, even where systems tend towards a balanced state, external agents all along the value chain, such as early adopters of new technologies, can disrupt this state until a new balanced state develops. A series of system-level failures have been proposed as preventing the creation and operation of genuine circular economy (Velis, 2018): description, verification and quantification of such phenomena, however, would necessitate a systems approach. Quantifiable metrics of circularity could be exhibiting highly non-linear behaviours, as a system emerges through the adaptive nature of agents. However, the BS ISO 59004:2024 does not specifically mention or define *‘micro’*, *‘meso’* and *‘macro’* level implementation of CE, and their associated modelling of complexity (The British Standards Institution, 2024a).

Therefore, even though BS ISO 59020:2024 provides an indicative list of circularity indicators, we argue that ultimately, effective qualification and quantification of circularity by default can be only achieved through a so-called ‘systems thinking’ approach. Additionally, BS ISO 59020:2024 mentions that value in CE systems is *“complex, and difficult to measure, and requires careful consideration”*. But it does not provide or propose a structured process to model ‘value’ (The British Standards Institution, 2024b). Simply put, systems thinking adopts holistic and interconnected approaches to understanding and addressing inherent complexities of an entire system, rather than its isolated components (Arnold and Wade, 2015; Bassi et al., 2021; Demartini et al., 2023). Richmond (1994) defined systems thinking as the art and science of making trustworthy decisions about behaviour by developing deep understanding of underlying structures. Meanwhile, Sterman (2000) defined systems thinking as an approach and mindset for understanding and analysing complex systems as interconnected and dynamic entities, and thus asserting the principle of dynamic complexity for solving systemic challenges. It involves recognising that a system is more than the sum of its parts; and that its behaviours and properties emerge from interactions and interdependencies among its components. A methodologically analytical approach therefore has the potential to identify leverage points in the system under a multiplicity of conditionalities.

Systems thinking and its pertinence to CE are discussed in detail S.3.1 and S.4 of the Supplemental sections (denoted as **S** for tables, figures and sections). Two prominent approaches in this regard are system dynamics (SD) modelling and agent-based modelling and simulation (ABMS). System dynamics and ABMS approaches can, to varying degrees, address challenges around static and linear descriptions of circularity. While Supplemental sections S.3.2 and S.3.3 offer more details of system dynamics and ABMS theorising, the approaches are summarised below. System dynamics models enable us to analyse the dynamic non-linear relationships and time-dependent pattern changes of complex systems. It considers the complexity of systems thinking by employing feedback loops, stocks and flow diagrams, time delays, nonlinear relationships, and causal loop diagrams, while adopting an iterative approach. Whereas ABMS is a computational technique used to simulate complex systems composed of autonomous entities called agents. Each agent has its own behaviour, decision-making rules, and interactions with other agents and the environment. This approach has been widely adopted for its ability to model complex human-environmental systems by capturing the dynamics and heterogeneity of individual actors.

To date, applied research on CE and systems thinking has often focused on complexity modelling of the *‘micro’* scale. For example, addressing the meaning of systems thinking in a CE context, but not the inherent complexities and dynamicity of the whole system in *‘macro’* scale (Iacovidou et al., 2021). Recent research has advanced CE, but often still omitting systems thinking and even more so its corresponding quantification (Table 2), which, as we argue, could provide a sound base for more holistic, meaningful and reliable circularity analytics.

**Table 2.** Reviews on systems thinking and its quantification from a CE perspective.

Summary	Scope limitations	References
Focused on common modelling approaches to analyse industrial symbiosis.	Did not focus on dynamics of circularity metrics and parameter quantification.	Demartini et al. (2022)
Reviewed life cycle assessment studies of a biorefinery system.	Omitted quantification of systems thinking by complexity modelling.	Vance et al. (2022)
Integrated review of CE concerning construction and the built environment.	Omitted systems thinking and dynamic quantification of CE.	Çimen (2021)
Comprehensive review on adoption of system dynamics and agent-based models in construction waste management.	Did not focus on the necessity of systems thinking, its complexity, and thereafter dynamic quantification of circularity metrics.	Ding et al. (2018)

CE: circular economy.

To the best of our knowledge, there are no reviews and analyses with a comprehensive scope on the quantification of circularity through systems thinking and its associated complexity modelling. Therefore, here we adopt a systematic scoping review approach according to Preferred Reporting Items for Systematic Reviews and Meta-Analyses for Scoping Reviews (PRISMA-ScR; Peters et al., 2020; Tricco et al., 2018), concerning the application of systems thinking through system dynamics and ABMS (refer to Supplemental sections S.5–S.8 for more details). Our approach and scope can offer novel insights on:

(a) considering how complexity arises in CE systems, highlights the relevance of systems thinking applied to CE, and particularly its quantification;
(b) by the way of case studies categorised by sector, it applies a magnifying lens on applications of system dynamics and/or agent-based modelling in quantifying different aspects of CE in the bio-based, construction, electrical and electronics products, single materials, manufacturing, industrial symbiosis and miscellaneous sectors, specifically also considering circularity metrics and wider parameters, which BS ISO 59004:2024 and BS ISO 59020:2024 do not provide; and,
(c) by describing and analysing how other decision-support tools have been integrated with system dynamics and ABMS aiming at more refined analytics.

Additionally, we also consider case study geography, along with the impact of policy and governance aspects, where available. Notably, we do not analyse, criticise or comparatively examine the specific findings of each publication we have reviewed here, but we instead focus on documenting and examining their methodological approaches to quantifying circularity via systems methods.

## Methodological approach to systematic literature review

**Figure 1.**
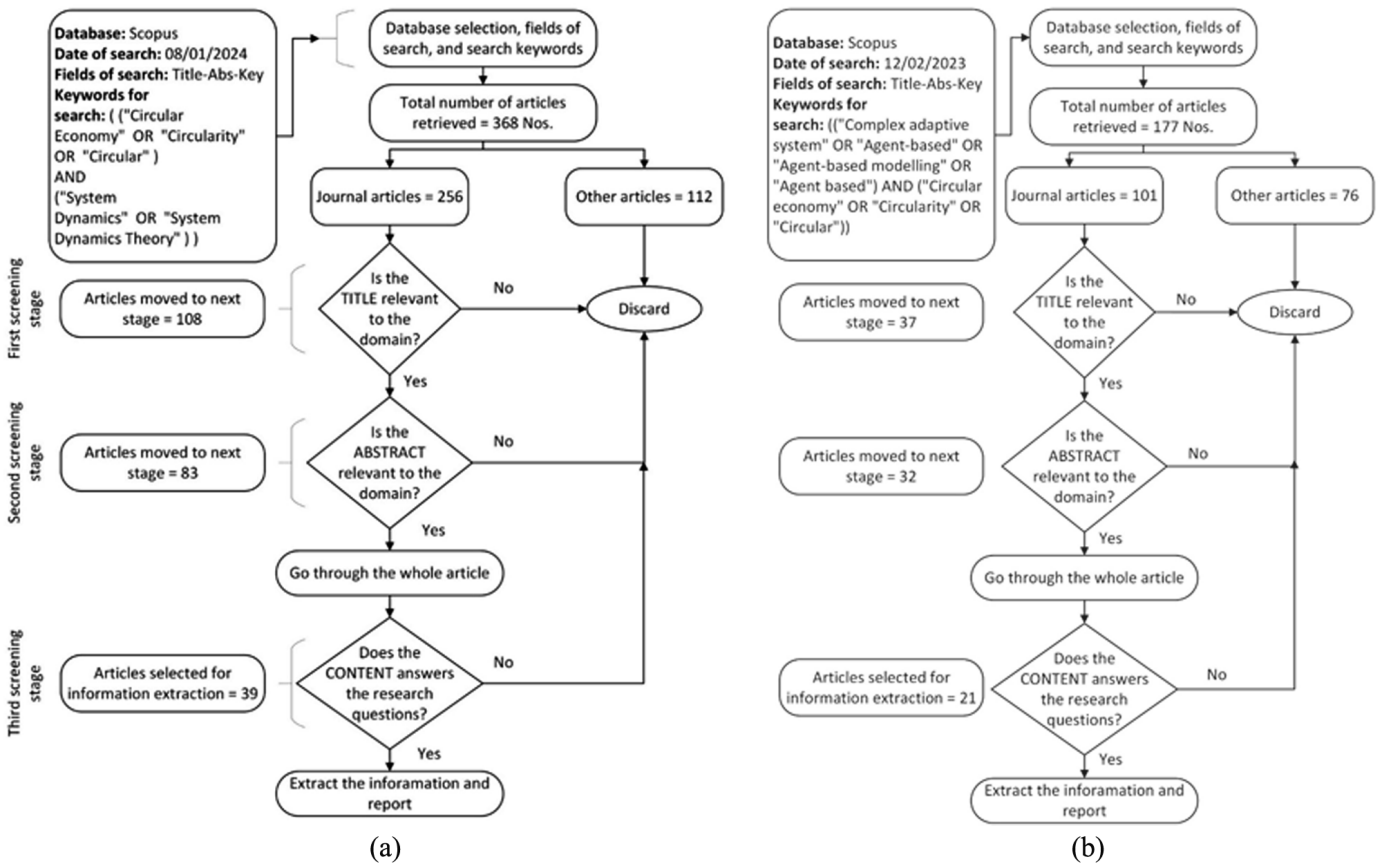
(a) Literature selection flow diagram to identify key publications related to applications of system dynamics in the circular economy context. (b) Literature selection flow diagram to identify key publications related to applications of agent-based modelling and simulation in circular economy context.

We conducted a systematic scoping review following the PRISMA-ScR guidelines (Peters et al., 2020) and the associated preferred reporting items checklist (Tricco et al., 2018; refer Figure 1(a) and (b) and Supplemental Table S.3). The review explored three specific questions:

- RQ1: What were the contexts/necessities of applying system dynamics modelling and ABMS approaches in CE?
- RQ2: Which types of decisions were provided through system dynamics and ABMS approaches?
- RQ3: Were any other tools/methods applied in conjunction with system dynamics, and ABMS? If so, why?

**Figure 2.**
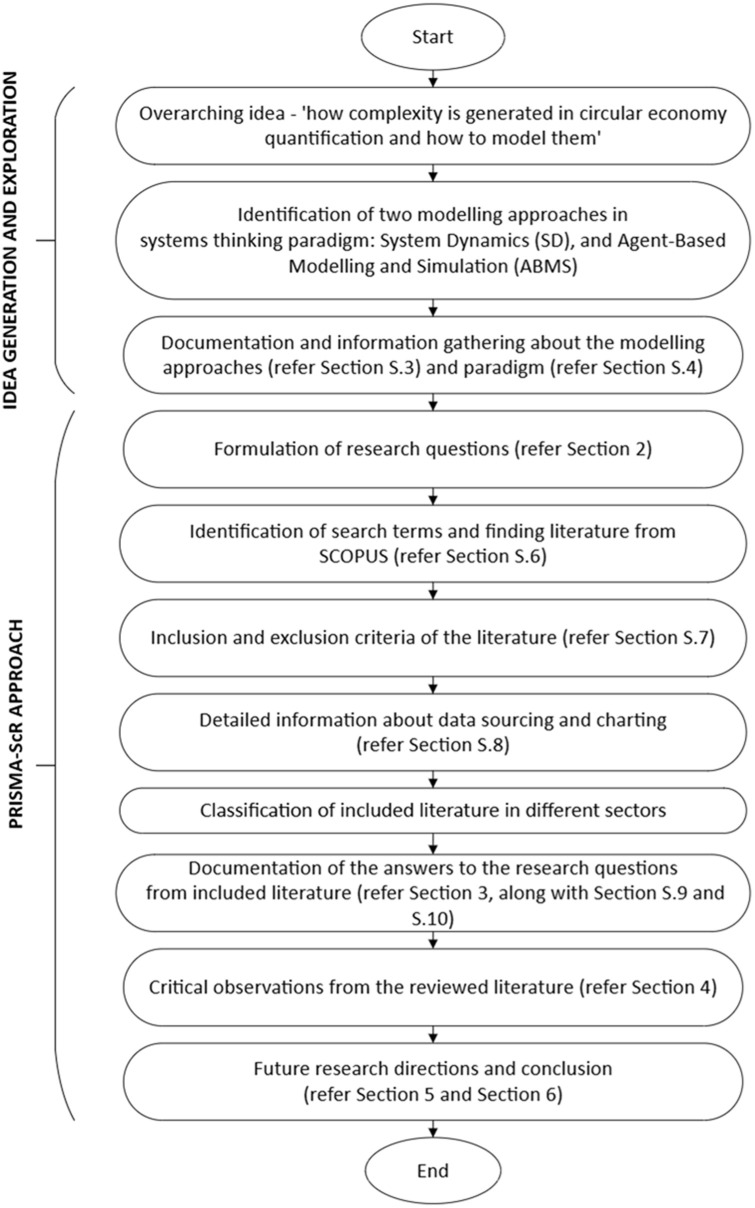
General methodological flow diagram and signposting of information for this research.

The complete flow diagram of the work is presented in Figure 2. The search was practically restricted to recently published articles, arbitrarily defined as in the last 9 years at the point of the start of the review: 2016–2024. However, this approach was justified by looking at earlier outputs, where only three relevant articles were retrieved from 2003 to 2015, and these were not useful to answer the research questions. Having identified the articles which answered the research questions, these were grouped according to application domain. This identified the following sectors: bio-based, construction, electrical and electronic products, single materials, manufacturing, industrial symbiosis and miscellaneous. The precise definitions of these sectors are given in the relevant sections.

## Sector-wise applications of SD and ABMS in quantitative circular economy

### Bio-based sector

We have used the term bio-based sector to refer generally to material of organic or biological origin. Exact application domains are given in Tables 3 and 4.

**Table 3.** Case-study domains, quantified metrics, consideration of ‘R’ principles and use of other tools in system dynamics applications within the bio-based sector.

Case-study domain	Metrics and/or parameters quantified	Which ‘R’s were considered?	Use of other tools	References
Chicken farming	Behaviour of egg laying chickens, egg, hatchery, and day-old chick stocks, poultry litter and poultry litter to banana farm stock, chicken flow to slaughterhouse, waste flow to fish farm, total income, profit and total cost of the stakeholders	Reuse, repurpose, recycle, recover	n.a.	Abbasi et al. (2024)
Food-waste mitigation strategy by food-sharing platform	Total amount of food waste prevented, total CO_2_ emissions prevented and food-sharing platform performance.	Rethink, reduce and reuse	Bass diffusion model to account the adoption of food-sharing app by the population.	Ranjbari et al. (2024)
Food waste reduction	Intermediate cost, total gross value added (GVA) and sectoral GVA, severe food insecurity rate, food waste generation, redistribution cost, redistribution benefit	Reduce, reuse, recycle, recover	n.a.	Parsa et al. (2024)
Food waste reduction in FEWC nexus	Food footprint, food waste generation, energy footprint, water footprint and carbon footprint	Reduce, reuse, recycle, recover	Group model building to create, refine, and restructure the system dynamics model with stakeholders’ interventions.	Parsa et al. (2023)
Food and agricultural system	Total reactive nitrogen output, air nitrogen emission, soil reactive nitrogen emission, water reactive nitrogen emission	Reuse and recycle	n.a.	Xing et al. (2023)
Fuel: waste cooking oil to second-generation fuel	Household consumption and industrial consumption of vegetable oil, waste oil not recycled, water pollution and oil to recycle	Recycle	n.a.	de Carvalho Freitas et al. (2022)
Food, energy, water, waste nexus	Reclaimed water utilisation, food waste reuse, waste to energy generation, food production, carbon footprint, water footprint, water resilience index, food supply index and different types of wastes produced	Reduce, recycle, reuse, recovery	TOPSIS, a MCDM tool was used to identify the most suitable scenario based on sustainability and resilience indicators.	Valencia et al. (2022)
Phosphorus (P) recycling	Worker safety, employment equality, employment rate, poverty rate, child labour, water use, nutrition supply, P efficiency and security	Recycle and recovery	n.a.	El Wali et al. (2021)

n.a.: not applicable; GVA: gross value added; FEWC: food, energy, water and climate; TOPIS: technique for order of preference by similarity to ideal solution; MCDM: multi-criteria decision-making.

**Table 4.** Case-study domains, quantified metrics, consideration of ‘R’ principles and use of other tools in agent-based modelling applications within the bio-based sector.

Case-study domain	Metrics and/or parameters quantified	Which ‘R’s were considered?	Use of other tools	References
European organic waste treatment facilities	VFAP adoption rate by changing the: (a) subsidies (both investment and operational), (b) market growth in PHA segment, (c) improvement in technological efficiencies, (d) social pressure.	Recycle	n.a.	Farahbakhsh et al. (2023)
Carbon containing waste of MSW	Climate change, terrestrial acidification, fossil resource scarcity, system cost and impact on local environment for different defined scenarios.	Recycle	n.a.	Voss et al. (2022)
Household recyclable wastes	Participation rate of households regarding three choices: landfilling, placing waste at a central container or being collected, every day for a period.	Recycle, recovery	TPB was used to identify key influencing factors affecting the residents’ inclination to recycling.	Tong et al. (2018)

n.a.: not applicable; VFAP: volatile fatty acid platform; PHA: polyhydroxyalkanoates; TPB: theory of planned behaviour; MSW: municipal solid waste.

#### System dynamics modelling in bio-based sector

Articles cover a diverse range of contexts, such as; food waste reduction in the food, energy, water and climate (FEWC) nexus (Parsa et al., 2023, 2024) or through an online food sharing application (Ranjbari et al., 2024), the flow of chicken and associated waste products in the economy (Abbasi et al., 2024), addressing reactive nitrogen’s societal contribution (Xing et al., 2023), utilising waste cooking oil as second-generation fuel (de Carvalho Freitas et al., 2022), examining urban sustainability transition in food, energy, water and waste (FEWW) sectors (Valencia et al., 2022) and scrutinising phosphorus recycling process (El Wali et al., 2021).

The initial step of system dynamics modelling involves the creation of causal loop diagrams, offering a qualitative understanding of intricate relationships between different parts. Afterwards, stocks and flows within the system are identified, and quantitative analysis is executed by stocks and flow diagrams. Some authors exclusively presented the causal loop diagrams before developing the stocks and flow diagrams (Abbasi et al., 2024; de Carvalho Freitas et al., 2022; Ranjbari et al., 2024; Xing et al., 2023), whereas some incorporated the causality of different variables within the stocks and flow diagram (Valencia et al., 2022). Some did not report the stocks and flow diagram directly, but presented the flows of materials, energy or finance through Sankey diagrams (Parsa et al., 2023, 2024).

Stocks and flow diagrams were used to illustrate the dynamic interactions of flows of FEWC between different sectors in an agri-food supply chain (Parsa et al., 2023), to highlight socio-economic interactions coupled with material and finance flows (Parsa et al., 2024), and to integrate the forward and reverse value-chain mapping in chicken farming (Abbasi et al., 2024). Furthermore, they aided the understanding and mapping of flows within various scenarios: for example reactive nitrogen flows from food waste and its associated impact on the nitrogen cycle (Xing et al., 2023); waste cooking oil generation pattern by households, and its impact on water pollution (de Carvalho Freitas et al., 2022); FEWW flow (Valencia et al., 2022). Some authors even modelled material flows (viz., surplus food) with the knowledge of the system entities (viz., population; Ranjbari et al., 2024) and coupled materials flow with social metrics (El Wali et al., 2021).

System dynamics models are in principle able to identify key leverage points of systems, and in scenario generation and metrics forecasting, which, in turn, could help policy recommendations. Key leverage points may not be obvious in baseline scenarios but emerge through examining various alternative scenarios. For instance, crop planting and aquaculture were identified through scenario generation as the main sectors for emitting reactive nitrogen (Xing et al., 2023). In de Carvalho Freitas et al. (2022), household cooking oil consumption patterns were identified as the key influencer on water pollution. Meanwhile, food waste and environmental footprints could be minimised by reducing waste generation at consumer and redistribution levels (Parsa et al., 2023). Children’s knowledge of a food-sharing app was identified as a key lever to reducing food waste (Ranjbari et al., 2024). In Parsa et al. (2024), the key sectors for food waste minimisation were identified which, in turn, benefited the socio-economic metrics. Some studies demonstrated self-sustainability through landfill gas and stormwater reuse (Valencia et al., 2022). Holistic linkages between small and medium-scale farms have been emphasised for sustainable development (Abbasi et al., 2024). Additionally, the practice of CE enhanced global phosphorus security and social prosperity in low and middle-income countries as found in El Wali et al. (2021).

Additionally, all these studies mapped stocks and flows over extended time periods, across various geographical scales (El Wali et al., 2021) and coupled them with various CE metrics (see Table 3), not only to map the current situation, but also to forecast future scenarios. The variables considered in the models were often non-linear. For details, refer to Supplemental Tables S6 and S7.

#### ABMS in bio-based sectors

ABMS is a bottom-up system modelling approach. In Farahbakhsh et al. (2023), it was utilised to identify the barriers to adopting emerging technologies in waste treatment plants, exploring the potential contribution of chemical recycling of carbon-containing waste of municipal solid waste (MSW). It has also been used for dynamic life-cycle sustainability assessment (LCSA) to contribute to UN sustainable development goals (SDGs) (Voss et al., 2022), and to investigate households’ intentions regarding recycling (Tong et al., 2018).

In general, we sense that ABMS is preferred over systems dynamics when the heterogeneities of the stochastic agents are essential to modelling. In Farahbakhsh et al. (2023), it was induced by agents’ (viz., waste treatment plants) environmental awareness, investment required to adopt emerging technologies, and expected returns, etc. In Voss et al. (2022), agents were characterised based on their geographic locations, annual residual MSW production volume, treatment capacity, etc. In Tong et al. (2018), households’ intentions towards recycling and/or landfilling were considered as heterogeneities.

These heterogeneous agents sustain by dynamically adapting to their environment. For example, in the case of valorising organic waste into high-end products, the agents adapted within a global market in response to economic, social and environmental pressures (Farahbakhsh et al., 2023). Strict sustainability regulations led agents (viz., administrative areas and waste treatment plants) to adapt in Voss et al. (2022) regarding circular utilisation of organic residual MSW as a chemical feedstock via gasification. While in Tong et al. (2018) waste separation and recycling were agents triggered by changes in consumer behaviour.

Interactions between agents and with the environment can lead to the emergence of new, not easily foreseeable, behaviours. This can result from social pressure provided by other agents to adopt CE technologies (Farahbakhsh et al., 2023), or be reward-driven (Tong et al., 2018). Temporal (Farahbakhsh et al., 2023; Tong et al., 2018; Voss et al., 2022) and spatial considerations (Tong et al., 2018; Voss et al., 2022) offer a nuanced understanding of dynamic processes. This consideration over temporal scales led to scenario analysis and metrics quantification as outlined in Table 4 (for further details, see Supplemental Tables S20–S22).

### Construction sector

The construction sector refers to anything related to construction, and a detailed consideration of the domains covered is presented in Table 5. No studies were identified that use ABMS to quantify circularity.

**Table 5.** Quantified metrics, consideration of ‘R’ principles in system dynamics applications within the construction sector.

Case-study domain	Metrics and/or parameters quantified	Which ‘R’s were considered?	Use of other tools	References
Recycled paver block circularity	Manufacturing cost of recycled paver block	Recycle	n.a.	Gandhi et al. (2024)
Decarbonisation potential of US commercial buildings	Annual commercial floorspace, embodied emissions	Reuse, reduce,	n.a.	Eissa and El-adaway (2024)
Policy impacts on CE transition within the construction industry	Policy supports, organisational incentive scheme and sustainable development	n.a.	n.a.	Ghufran et al. (2022)
Circularity potential of recycled building materials to replace virgin materials	Demand for aggregates for concrete, demand for recycled aggregates for concrete, savings in sand and gravels, unused recycled aggregates	Reduce, recycle	n.a.	Mostert et al. (2022)
Barriers in adopting CE transition in the construction industry	Recycling and recovering quota of CDW and excavation materials, primary gravel price, disposal price	Reduce, recycle, recovery	n.a.	Kliem et al. (2021)

n.a.: not applicable; CE: circular economy; CDW: construction and demolition waste.

#### System dynamics modelling in the construction sector

System dynamics has been used for a range of applications in the construction sector. It was used to examine the circularity potential of recycled paver blocks (RPBs) Gandhi et al., 2024), and the decarbonisation potential of US commercial buildings (Eissa and El-adaway, 2024). Ghufran et al. (2022) explored enablers for the CE transition in the construction sector, focusing on causal interdependencies, while Kliem et al. (2021) specifically concentrated on policy. Mostert et al. (2022) meanwhile estimated and forecast construction and demolition materials flows in the German construction sector.

Causal loop diagrams have been constructed to depict the dynamic interdependencies between cost components when manufacturing RPBs (Gandhi et al., 2024), to examine causal links between CE enablers in the construction industry (Ghufran et al., 2022), and public policy instruments’ impacts on CE business model (Kliem et al., 2021). Stocks and flow diagrams meanwhile aided quantification of the temporal evolution of manufacturing cost of RPBs (Gandhi et al., 2024), flows of newly built floorspace, demolished floorspace, total carbon emissions associated with concrete and emissions per unit floorspace (Eissa and El-adaway, 2024). They have also been used to examine organisational incentive schemes, policy supports and sustainable development (Ghufran et al., 2022), flows of demolished materials in the economy (Mostert et al., 2022), and in modelling sand and gravel quarry and disposal volumes (Kliem et al., 2021). Thus, stocks and flow diagrams prove versatile, not only capturing material flows, but also evolving policy variables (Ghufran et al., 2022).

All these studies considered the temporal evolution of dynamic systems, and subsequent scenario analysis led to recommendations based on reported metrics (see Table 5) and identifying the key leverage points. For instance, Gandhi et al. (2024) identified contractor profit, overhead expenses and labour costs as the main barriers to using RPBs in India. Eissa and El-adaway (2024) suggested that a comprehensive CE policy in the United States could deliver a 52% decarbonisation potential by 2050, while policy support and incentive schemes have been recognised as key factors for CE transition in Ghufran et al. (2022).

Delay modelling, another key feature of system dynamics, as adopted by Kliem et al. (2021) and Mostert et al. (2022). The CE transition in the construction sector not only requires adequate recycling, but also a constant supply of secondary materials. While Ghufran et al. (2022) considered global participants, all other studies were conducted at the micro or meso-scale. For further information see Supplemental Tables S8 and S9.

### Electrical and electronics products sector

As the name suggests, this sector comprised studies considering electrical and electronic products. It encompassed waste from electrical and electronics equipment (WEEE), batteries, mobile phones, photovoltaics, computer equipment and household white goods. Details about the specific case-study domain can be found in Tables 6 and 7.

**Table 6.** Case-study domains, quantified metrics, consideration of ‘R’ principles and use of other tools in system dynamics applications within the electrical and electronics products sectors.

Case-study domain	Metrics and/or parameters quantified	Which ‘R’s were considered?	Use of other tools	References
Waste lithium	Lithium demand by application, lithium content in waste stream and lithium accumulation	Reduce, refuse, repurpose, remanufacture, recycle and reuse	n.a.	Lähdesmäki et al. (2023)
Waste from electrical and electronics equipment (EEE)	Material extraction, official EEE collection, inadequate disposal of EEE, ratio of material treated and ratio of material lost	Reuse, recycle	n.a.	Guzzo et al. (2022b)
Waste from electrical and electronics equipment	Official EEE collection, inadequate disposal of EEE, ratio of material treated in the last years	Recycle, reuse, recovery	n.a.	Guzzo et al. (2022a)
Waste from electrical and electronic equipment	Availability of raw material, material extraction, EEE commissioning, total EEE in use, disposal of EEE as WEEE, WEEE recycled	Reuse, remanufacture, repair, recycle, recovery	Bass diffusion model to model the diffusion of technology in the system.	Guzzo et al. (2021)
Waste from electrical and electronics equipment	AEB, repurchase price, WEEE generated, profits	Repair, recycle, remanufacture, recovery	Mixed-integer non-linear programming approach was used to fine tune the system dynamics parameters.	Llerena-Riascos et al. (2021)
EOL management of photovoltaic (PV) solar cells	Collection fraction, collection rate, total recovered materials, landfill amount, payback period and number of dwellings with photovoltaic (PV) solar cells	Reuse, repair, recycling, recovery	Quantitative–qualitative triangulation method was used to capture the mental models of the stakeholders.	Salim et al. (2021)
Gold recovery from cell phone recycling	Depletion/savings of gold reserve, gold from E waste, gold from discarded cell phones, economic benefits, environmental benefits, social benefits (job creations), collection efficiency	Reuse, refurbish, recycle, recovery	n.a.	Chaudhary and Vrat (2020)
E-waste reverse supply chain	Depletion of material reserve, material conversion rate, extracted material, economic, environmental and social benefits, increase of collection coverage, and pre-processing capacity, demand of recycled materials, change in recycled products with change in refurbish and reuse rate, etc.	Reuse, refurbish, recycle, recovery	n.a.	Chaudhary and Vrat (2019)
Electric vehicle batteries	Gross benefit of remanufacturing, remanufacturing margin, and price for remanufactured and new electric vehicle batteries	Reuse, remanufacture, repurpose, recycle, recovery	n.a.	Alamerew and Brissaud (2020)
Mobile phone recycling	Gold use by phone manufacturers and gold recovery at the end-of-life of phone, loop leakage, closed loop efficiency of global mobile phone product systems	Reuse, refurbish, recycle, recovery	Opt Quest optimiser to optimise high and low sensitive parameters.	Sinha et al. (2016)

n.a.: not applicable; AEB: avoided environmental burden; WEEE: waste electrical and electronics equipment; EEE: electrical and electronic equipment; EOL: end-of-life.

**Table 7.** Case-study domains, quantified metrics, consideration of ‘R’ principles and use of other tools in agent-based modelling applications within the electrical and electronics products sectors.

Case-study domain	Metrics and/or parameters quantified	Which ‘R’s were considered?	Use of other tools	References
End of management of magnets of hard-disk drives	Recovered mass of REE, material value, and avoided GHG emission by recycling, HDD reuse, and magnet reuse, enhancement of trust towards data wiping.	Reuse, recycle, recovery	TPB was used to model customers’ intention to reuse/recycle the hard-disk drives. Data uncertainties and qualities were modelled through data pedigree matrix.	Walzberg et al. (2022a)
Photovoltaic (PV) cell circularity	Recycling costs, material recycling rate, material recovery rate, initial recycling costs, recyclers’ net income, reuse rate, landfill costs, societal costs	Recycle, reuse, recovery	TPB was used to model decision-making for PV cell purchase/reuse.	Walzberg et al. (2021)
Waste electrical appliance recovery industry	Waste appliance generated per capita, extent of environmental pollution perceived by residents, service level of each recycler perceived by the residents, residents’ recycling tendency with respect to each recycler, recovery rate, policy costs, profits of the agents.	Recycle, recovery	TPB was used to model residents’ recycling behaviour.	Luo et al. (2019)
Cell phone leasing	Total buy/lease decision count, tendency to lease next cell phone if the lease term is increased, percentage of leases over time for different new product prices.	Reuse, recycle, recovery	Discrete choice analysis to predict future market demand considering social influences of new product adoption.	Mashhadi et al. (2019)
Washing machine	Number of customers served, aggregated lifecycle cost, aggregated lifecycle impact, and material savings.	Reuse, remanufacture, recycle	Discrete event simulation was used to model the closed loop-supply chain movements.	Lieder et al. (2017b)
Washing machine	Market share, customer satisfaction, price, environment friendliness, service.	Refurbish, reuse, recycle, recovery	n.a.	Lieder et al. (2017a)

n.a.: not applicable; REE: rare earth element; GHG: greenhouse gas; HDD: hard-disk drives; TPB: theory of planned behaviour.

#### System dynamics modelling in the electrical and electronics sectors

Lähdesmäki et al. (2023) modelled the CE potential of lithium in a global context, while Guzzo et al. (2021, 2022a, 2022b) showed how CE policy interventions facilitated nationwide electrical and electronics waste collection and treatment. Llerena-Riascos et al. (2021) explored the operative-strategic interdependency in improving the representation and performance of WEEE collection and processing steps. Salim et al. (2021) delved into socio-technical transition pathways for end-of-life management of rooftop photovoltaic solar panels. In both works, Chaudhary and Vrat (2019, 2020) and Sinha et al. (2016) used systems dynamics to investigate circularity regarding mobile phones. Chaudhary and Vrat (2019, 2020) highlighted the drivers for proper circular flow of gold in mobile phones, while Sinha et al. (2016) drew attention to the potential of closing material flow loops in global mobile phone product systems, addressing sustainability challenges of material recovery.

Causal loop diagrams have been key in offering an explicit representation of dynamic interdependencies (Alamerew and Brissaud, 2020; Chaudhary and Vrat, 2019, 2020; Salim et al., 2021), while others presented causality passively (Guzzo et al., 2021, 2022a; Sinha et al., 2016). Facilitation of CE was found to be not only dependent on cost and revenues, but also on strategic regulatory decisions, and proper policy implementation (Alamerew and Brissaud, 2020; Chaudhary and Vrat, 2019). Whereas, circular value-chain mapping of gold, e-waste and mobile phones with their sustainability benefits were evident in Chaudhary and Vrat (2019, 2020) and Sinha et al. (2016), where sustainability indicators were causally linked. Additionally, inappropriate disposal of hazardous waste was addressed in Salim et al. (2021), urging proper recovery and recycling.

Stocks and flow diagrams were prevalent throughout the literature, effectively representing material flows inside system boundaries. However, Guzzo et al. (2021) used them to represent the flow of technology adoption processes, and Salim et al. (2021) for recycling fund flow. Delay and lifetime distribution modelling through different distributions were shown in Guzzo et al. (2021, 2022a, 2022b). Notably, Guzzo et al. (2022a) presented an inductive–deductive system dynamics model, where the empirical theory was constructed in the inductive stage, and then deductive theory was tested in another case study.

All the studies reviewed in this sub-section explored the common capabilities of system dynamics modelling – temporal dynamics, scenario analysis, metrics quantification and thereby recommendations (see Table 6). The work of Sinha et al. (2016) was a global-scale study. In scenario analysis, multiple previous scenarios can be superimposed (Guzzo et al., 2021; Lähdesmäki et al., 2023). Specifically, Lähdesmäki et al. (2023) recommended to implement a combined CE policy, informed by such multiple scenarios. Guzzo et al. (2021) suggested the systemic change for CE implementation. While Salim et al. (2021) found that there were high uncertainties in waste collection, recovery performance and landfill disposal.

System dynamics can also handle uncertainties of the parameters, and can provide decisions accounting, as shown by Salim et al. (2021). Chaudhary and Vrat (2020) underscored the value of consumer awareness and stakeholder incentives for better CE implementation, while their earlier work (Chaudhary and Vrat, 2019) emphasised the policy interventions for addressing challenges associated with the informal handling of e-waste without environmental and health protection. Meanwhile, Sinha et al. (2016) recommended closed-loop recycling through improved collection systems, longer mobile phone retention time, improved recycling in developing countries and shorter phone hibernation times. This work proposed reducing informal recycling of e-waste, as they claim that it leads to lower resource recovery along with higher pollution. Further details are in Supplemental Tables S10 and S11.

#### ABMS in the electrical and electronics sectors

ABMS has been employed to assess the influence of techno-economic and social factors on the circularity of hard-disk drives (Walzberg et al., 2022a). The importance of considering social factors in empirical CE analysis has been underscored, alongside techno-economic considerations (Walzberg et al., 2021). ABMS was also adopted to model waste household appliance recovery (Luo et al., 2019). Mashhadi et al. (2019) stressed that product service systems, such as mobile phone leasing, can overcome CE implementation barriers. ABMS was instrumental in quantifying demand behaviour for circular business models (leasing or functional sales; Lieder et al., 2017a). Another study by the same authors employed multi-method simulation approach combining ABMS and discrete event simulation (DES) to quantify design efforts for circular options (reuse, remanufacture and recycle) and supply chain settings (buy-back, pay-per-use and leasing; Lieder et al., 2017b). The details of each study and the types of agents can be found in Supplemental Tables S23–S25.

The ability of ABMS to capture the agents’ distinct characteristics, attributes, behaviours and decision-making rules, that is, heterogeneity, has been useful in tracking agents’ specific CE pathways (Walzberg et al., 2021). By integrating the theory of planned behaviour (TPB) and its associated factors, Walzberg et al. (2022a) determined how the agents’ decisions at the individual level led to changes at the systemic level, specifically in their adoption of CE pathways. The heterogeneity of residents, governments and recycling agents was considered by Luo et al. (2019), who included factors such as waste appliance generation, industrial standards development, tax incentives, distances from residents, etc. The ‘memorising’ capability of agents was demonstrated by Lieder et al. (2017a) and Mashhadi et al. (2019) where previous positive experiences influenced the subsequent decisions regarding adopting CE options (e.g. buying/leasing/pay-per-use, etc.). Meanwhile, heterogeneity demonstrated a single component’s four distinct stages (Lieder et al., 2017b): manufactured, assembled in product, disassemble after use and material recovered.

With more knowledge of the positive impacts of CE, agents can be self-encouraged, that is, show adaptation. While agents always try to choose a position where their benefits are maximised, the rewards are dependent on the path taken (Luo et al., 2019). Adaptation has been demonstrated due to socio-economic and social status (Walzberg et al., 2021, 2022a), environment-friendly awareness and peer-pressure (Mashhadi et al., 2019), socio-demographics, social networks and product utility (Lieder et al., 2017a), and proper marketing and pricing strategy (Lieder et al., 2017b). Complex interactions between agents, and their stochastic natures, were consistently emphasised in each work. ABMS has been employed to represent the flow of materials (Lieder et al., 2017b; Luo et al., 2019; Walzberg et al., 2021), and also spatial considerations (Luo et al., 2019; Walzberg et al., 2021). Meanwhile, agent heterogeneity was obtained from survey data by Mashhadi et al. (2019) and used within an ABMS framework.

Scenarios analysis led to recommendations, as evidenced by metric quantification, for achieving increased circularity (see Table 7). For instance, the reuse of hard-disk drives was found to be more environmentally friendly (Walzberg et al., 2021). Additionally, CE practice was found to be facilitated by proper education on recycling and recovery options, effective regulatory frameworks, technological innovations, improving product eco-design and establishment of waste appliance recovery networks (Luo et al., 2019). Scenario analysis also revealed agents’ previous positive experience on buying/leasing affects further buying/leasing decisions (Mashhadi et al., 2019). The *‘pay-per-use’* approach supported by advertisements provided environmental friendliness and service-orientation. This was further aligned with a *‘buy-back’* scenario for more returns after a certain period in Lieder et al. (2017a).

### Circularity of single materials

The following sub-section describes the quantification of different aspects of circularity of specific single materials. This covers plastic bottles, platinum, zinc and copper. While the term plastics itself covers a broad array of materials, Ghosh et al. (2023) considered it as a single material. Note also that no studies were found related to the application of ABMS to quantify circularity of single materials.

#### System dynamics modelling for circularity of single materials

In a ‘learning-by-doing’-based system dynamics model, Ghosh et al. (2023) explored end-of-life pathways for Polyethylene Terephthalate (PET) bottles, calculating circularity indicators considering technological, economic, and policy constraints. Meanwhile, Saidani et al. (2021) employed a multi-method simulation approach to identify, classify and assess key parameters and action levers to close the material cycle loop of platinum in catalytic converters. System dynamics also modelled key endogenous policy impacts on the development of a circular model for zinc manufacturing growth (Ojha and Vrat, 2020), considering exploration, demand, collection, recycling, repair and reuse. Finally, Pfaff et al. (2018) applied system dynamics to examine sectoral primary and secondary copper flows and stocks in Germany.

Causal loop diagrams were presented by Ojha and Vrat (2020) and Saidani et al. (2021) to illustrate the dynamic connections between collection, recovery, circularity rate and influence of linear and circular economy factors affecting manufacturing output growth. Ghosh et al. (2023) didn’t show a stock-and-flow diagram, but conveyed it implicitly to perform the lifecycle and techno-economic analyses, which considered the flow of plastics. Stocks and flow diagrams have been presented to illustrate the flow of platinum (Saidani et al., 2021), zinc (Ojha and Vrat, 2020) and copper (Pfaff et al., 2018). Temporal evolution of material flows was intrinsic in each study. However, spatial aspects were considered less frequently (Ghosh et al., 2023; Pfaff et al., 2018).

With the exception of Saidani et al. (2021), all studies conducted scenarios analysis, leading to recommendations through metrics quantification (see Table 8). For instance, optimal PET circularity was when *‘recyclate glycolysis’* was adopted with improved collection access, due to increased replacement of virgin materials with recycled resin (Ghosh et al., 2023). Meanwhile, increased geological exploration, improved collection facilities, cost-effective remanufacturing technologies and better incentive schemes were recommended for improving zinc circularity (Ojha and Vrat, 2020). Finally, Pfaff et al. (2018) concluded that imported primary copper had detrimental environmental impacts. However, this work was contingent on extensive data requirements. See Supplemental Tables S12 and S13 for more details.

**Table 8.** Case-study domains, quantified metrics, consideration of ‘R’ principles and use of other tools in system dynamics applications concerning circularity of single materials.

Case-study domain	Metrics and/or parameters quantified	Which ‘R’s were considered?	Use of other tools	References
Plastics (Polyethylene Terephthalate [PET] bottles)	Circularity (closed loop circularity, open loop circularity, upcycling circularity, incineration circularity, average inflow outflow circularity, landfill diversion circularity) environmental impact (GWP in kg CO_2_ eq.) and life cycle costs	Recycle, recover	n.a.	Ghosh et al. (2023)
Platinum in vehicular catalytic converters	Platinum circularity, potential collection, collection of catalytic converters, recovery of platinum	Recycle and recovery	Fuzzy cognitive mapping to identify action levers, drivers, and parameters responsible for loop-closure. FAST and MICMAC to list the potential action levers and to select and cluster the key variables, respectively.	Saidani et al. (2021)
Zinc recycling	Behavioural trends of four variables: green reserve, extraction material, manufacturing output and total refeed for different developed scenarios	Repair, reuse, remanufacture, recycle, recovery	n.a.	Ojha and Vrat (2020)
Copper production and recycling sector	EOL collection rate and recycling rate, total copper input to domestic production of finished goods, total collected domestic EOL copper scrap, primary copper input to domestic production of finished goods.	Reuse, recycle, recovery	ASTRA-based macroeconomic model was combined with system dynamics to evaluate the economic aspects of the sector.	Pfaff et al. (2018)

n.a.: not applicable; PET: Polyethylene Terephthalate; GWP: global warming potential; FAST: functional analysis system technique; MICMAC: matrix impact cross-reference multiplication applied to a classification; EOL: end-of-life.

### Manufacturing sector

The following sub-section describes the applications of system dynamics as a modelling approach to quantify the aspects of circularity related to manufacturing. While this sector covered, amongst others, household white goods and electrical motors, the focus of the studies was on the manufacturing or remanufacturing process. No articles were found related to the use of ABMS for circularity quantification in this sector. Details about the domain can be found in Table 9.

**Table 9.** Case-study domains, quantified metrics, consideration of ‘R’ principles and use of other tools in system dynamics applications within the manufacturing sector.

Domain of case study	Metrics and/or parameters quantified	Which ‘R’s were considered?	Use of other tools	Reference
White goods manufacturing	Lifecycle costs, environmental performance (kg CO_2_ eq.), revenue streams and profit over time.	Reuse, refurbish, recycle, recovery	ABMS was adopted to describe the heterogeneity of the agents (i.e. manufacturers, customers, etc.). DES was utilised to describe the process flow (e.g. manufacturing processes and logistic activities).	Roci et al. (2022)
Hypothetical manufacturing firm	Products collected for recycling, recycled products inventory, recycling waste rate, recycling rate, recycled products sales rate, purchase rate, products in use, landfill products, products collected for recycling, etc.	Repair, reuse, refurbish, recycle	Bass diffusion model was adopted to model the shape of the distribution of the short and long-life product.	Franco (2019)
Remanufacturing process of electric motors (rotor and shaft)	Time spent for remanufacturing based on the CPQ value, effect of CPQ on reusable products.	Reuse, repair, refurbish, recovery	DES for enumerating the sequence of operations in a remanufacturing process.	Charnley et al. (2019)

CPQ: certainty of product quality; ABMS: agent-based modelling and simulation; DES: discrete event simulation.

#### System dynamics modelling applications in the manufacturing sector

A multi-method simulation approach was proposed for a circular manufacturing system (CMS) of washing machines (Roci et al., 2022), integrating ABMS, DES and system dynamics modelling. Franco (2019) demonstrated the impacts of product design (i.e. slowing resource flows through design for longevity, ease of maintenance and repair; and by design for disassembly and recycling), business models (i.e. slowing resource flow by product-service-system and maintenance and reuse) and post-use strategies on facilitating CE. In a theoretical study on data-driven circular economy, Charnley et al. (2019) developed the concept of certainty of product quality (CPQ) and applied it to electric motor circularity through remanufacturing, by integrating DES and system dynamics modelling.

Neither Charnley et al. (2019) nor Roci et al. (2022) developed causal loop diagrams, but they have been presented to highlight the causality between different indices (e.g. disassembly index, recyclability index, functional risk, etc.). Stock-and-flow diagrams accounted material flows (Charnley et al., 2019; Franco, 2019), as well as dynamic evolutions of finance, and emission flows (Roci et al., 2022).

With Austria as their system boundary, scenario analysis and recommendations driven by metrics quantification revealed the benefits of circular economy for washing machines (see Table 9; Roci et al., 2022). The work also found that manufacturing cost dominated lifecycle costs, followed by installation, refurbishment and deinstallation costs. However, environmental impact was dominated by the use phase, possibly due to the long lifespan of white goods. Franco (2019) observed that when perceived functional risks of recycled or reused products were low, it positively impacted circularity. Additionally, increased CPQ was helpful in promoting the CE as shown in Charnley et al. (2019). See Supplemental Tables S14 and S15 for more information.

### Industrial symbiosis

Industrial symbiotic networks (ISNs) represent clusters of firms engaged in exchanging residual materials, energy and information, with the overarching goals of fostering economic prosperity, social advancements and mitigating environmental impacts. They have been considered as ‘complex adaptive systems’ for compelling reasons: actors emerge into coherent forms over time, their adaptive nature reflecting their ability to change over time, self-organisation because of their ability to find new partners and collaborations based on mutual utility, path-dependency and non-linearity (Fraccascia and Yazan, 2018).

#### System dynamics modelling in industrial symbiosis

While ABMS is considered as more adept at capturing symbiotic patterns with heterogeneous agents in ISNs, some authors tried to model it through system dynamics models. A dynamic empirical evaluation model for capturing sustainable development of eco-industrial parks was introduced by Zhao et al. (2023), using an emergy index. Meanwhile, Morales et al. (2022) modelled a meso-scale CE implementation for bio-based industrial symbiosis (BBIS) of the sugar-beet value chain, emphasising the concept of viable value chain (VVC) during disruptive events (e.g. COVID-19, climate change). In a different context, Dong et al. (2017) proposed a system dynamics model to understand the long-term materials flow inside a coal power plant and a cement production plant for integrated circularity.

The inclusion of a causal loop diagram in Zhao et al. (2023) facilitated capture of the dynamics of different industrial sectors. Causality of economy and ecology by sharing of the (waste) materials was presented in Dong et al. (2017). The concept of emergy consists of money, material, and energy flows, and either or both were captured through stocks and flow diagrams in all studies (Table 10).

**Table 10.** Case-study domains, quantified metrics, consideration of ‘R’ principles in system dynamics applications for the manufacturing sector.

Case-study domain	Metrics and/or parameters quantified	Which ‘R’s were considered?	Use of other tools	References
Eco-industrial park	Economic development (EDR,^a^ EYR^b^), environmental compatibility (EWR,^c^ ELR^d^), social acceptability (ED,^e^ and CP^f^)	Not mentioned.	n.a.	Zhao et al. (2023)
Sugar-beet value chain	Sugar production, CO_2_ emission, stillage and bioethanol production	Recycle	n.a.	Morales et al. (2022)
Cement and coal production plant	Natural resource saving (e.g. gypsum, clay, limestone, coal consumption, water consumption), reduction of pollutants (SO_2_, smoke dust emission), carbon emission from calcination, costs savings, sales revenue, increase of costs of water, SDD of fly ash, de-sulphurised gypsum and slag generation, electricity power yield, cement yield	Reduce, recycle, repurpose, recover	n.a.	Dong et al. (2017)

n.a.: not applicable; SDD: supply demand difference.

a EDR is the ratio of total emergy use and industrial added value of the park in 1 year.

b EYR is the ratio of the total emergy output to the emergy purchased from society (e.g. fuels, goods and services).

c EWR is the ratio of the sum of emergy, with three wastes (viz., waste gas, wastewater, and solid waste) to the total emergy, which was used to measure the pressure of wastes on the ecosystem.

d ELR is the ratio of purchased and non-renewable local emergy to the free/renewable resources emergy.

e ED is the ratio of emergy created by production processes to the area of the eco-industrial park.

f CP is the ratio of available and per capita emergy usage.

All of these studies considered system boundaries at the meso-scale and included temporal dynamics. Through scenarios analysis, and metrics quantification (see Table 10), these studies provided insights for decision-making. Zhao et al. (2023) adopted a science and technology-driven scenario to deliver optimal sustainable development. Systematic consideration of the value chain in industrial symbiosis to prevent supply chain collapse during shocks was underscored in Morales et al. (2022), with their forecasts unveiling complexities overlooked by linear economic models. Meanwhile, efficient supply chain eco-design emerged as critical for circularity of two mutually dependent sectors (Dong et al., 2017). See Supplemental Tables S16 and S17 for details.

#### ABMS in industrial symbiosis

ABMS modelled actors’ behavioural patterns (viz., waste suppliers, waste processor), and their intricate relationships towards ISN robustness (in terms of network survival for a particular time period, and cash flows per tonne of waste) (Lange et al., 2021a). While the viability of ISN survivability was examined for two business models (Lange et al., 2021b), namely circular waste management and waste as by-product. The dynamics of the construction supply chain, focusing on recycled concrete aggregate in an ISN was presented by Yu et al. (2021). Geographically oriented symbiotic relationships were examined in Raimbault et al. (2020), and material flows in a complex stakeholders’ involvement network in Fernandez-Mena et al. (2020a, 2020b). Several aspects of ISN survival through information sharing (Fraccascia and Yazan, 2018), cost-sharing negotiations between companies (Yazan and Fraccascia, 2020), redundancy strategy (Fraccascia et al., 2020) and the impact of online platforms (Fraccascia, 2020) have been shown.

Agent heterogeneity (Supplemental Table S27) has been captured in terms of changing supply chain quantity, physical degradation of waste and chemical composition (Lange et al., 2021a, 2021b). Agents were also heterogeneous in terms of their quantity, location, storage capacity, material flow rate (destination agent), and various attributes for *‘vehicle’* agent (Yu et al., 2021), and through their demand-offer functions (Raimbault et al., 2020). Finally, agents were heterogeneous due to industry type plus waste processor (Fernandez-Mena et al., 2020a, 2020b) or input–output characteristics (Fraccascia, 2020; Fraccascia and Yazan, 2018; Fraccascia et al., 2020; Yazan and Fraccascia, 2020).

Emergent patterns due to agents’ adaptation capability ranged from leaving an ISN network (Raimbault et al., 2020), choosing new partners (Lange et al., 2021b), and navigating uncertain encounters with the environment (Fraccascia et al., 2020). Materials flow (Fernandez-Mena et al., 2020a, 2020b), and cash flow (Lange et al., 2021b) between agents have also been quantified. In Raimbault et al. (2020) and Yu et al. (2021), material flows were modelled stochastically. The spatial dispersions of the agents (Fernandez-Mena et al., 2020a, 2020b; Yu et al., 2021) were also contemplated through ABMS, proving beneficial over system dynamics.

The memorising capability of agents in ABMS was showcased through ‘fitness to process the waste’, ‘leaving threshold’ in Lange et al. (2021a). A similar ‘fitness function’, based on economic benefits, encouraged agents to stay in ISNs (Yazan and Fraccascia, 2020). How the agents’ activities were triggered by realising the material transfer from the vehicle partner was presented in Yu et al. (2021). Note that, unlike with system dynamics, all the agents in the ABMS are not required to function during the entire simulation period. For instance, allocation of materials to agents only when they have the capacity to accommodate it (Fernandez-Mena et al., 2020a).

Scenarios analysis, leading to recommendations through metrics quantification (see Table 11), was conducted across most studies, apart from (Lange et al., 2021a; Raimbault et al., 2020). Notably, changes in scenarios in ABMS allowed observations at both micro and system levels, presenting its unique advantage over system dynamics. Additionally, during scenario analysis in ABMS, new emergent patterns could develop, which is not the case in system dynamics modelling. For instance, economic behaviour preceded behavioural patterns, such as ISN partners leaving networks due to insufficient waste supply (Lange et al., 2021b). Yu et al. (2021) suggested that increasing upcycling efficiency and subsidising stakeholders play a pivotal role in RCA circularity. Distances between agents were key for ISN survival (Fernandez-Mena et al., 2020a; Raimbault et al., 2020). A circularity scenario has been reported to mitigate greenhouse gas emissions, but at a substantial trade-off between food production and livestock number (Fernandez-Mena et al., 2020b). Sensitive information sharing through gradual trust-building was emphasised in Fraccascia and Yazan (2018). Minimising the differences between input–output quantities of waste facilitated the ISN survivability (Yazan and Fraccascia, 2020). Transaction cost played a pivotal role in economic and environmental benefit provided by ISN (Fraccascia et al., 2020). While Fraccascia (2020) found that when over 60% of ISN members engaged with online platform sharing, partners who did not participate in the ISN were disadvantaged, and adversely impacted the environmental performance. For more information, see Supplemental Tables S26–S28.

**Table 11.** Case-study domains, quantified metrics, consideration of ‘R’ principles and use of other tools in agent-based modelling applications for industrial symbiosis.

Case-study domain	Metrics and/or parameters quantified	Which ‘R’s were considered?	Use of other tools	References
ISN partners: (a) large-scale agricultural area, (b) small-scale urban agricultural area focused on sustainable food production and recreation, (c) a business park	Cash flow per each industrial symbiotic network actor, and failure or success of network (robustness).	Recycle, recovery	TPB was used to model agents’ negotiation and self-evaluation process.	Lange et al. (2021a)
Bio-based material suitable for anaerobic digestion for processing local waste and energy production from biogas	Circular business model survival rate percentage, value captured or lost per actor for each of the scenario.	Reuse, recovery	TPB was used for bilateral negotiations between waste processor and suppliers.	Lange et al. (2021b)
Recycled concrete aggregate ISN	Delivered recycled concrete aggregates, reduced CO_2_ emissions and space of cooperation/industrial symbiosis probability between firms involved in the network for different scenarios.	Reuse, recycle	GIS was adopted to articulate the complex spatial relationships of industrial actors.	Yu et al. (2021)
Hypothetical symbiotic network	Total waste flow and relative cost.	n.a.	Multi-objective optimisation was adopted to minimise the cost and waste products given that agents were distributed in a different geographical location.	Raimbault et al. (2020)
Agro-food network	Nitrogen flow, CO_2_ emissions, number of local flows, CO_2_ eq. emitted per gigagram of protein and tera-calorie of metabolisable energy in food production, crop production, meat and milk production, animal feeding district balance, biogas and electricity production.	Reuse, recycle and recovery	GIS was adopted to highlight the distances between the agents.	Fernandez-Mena et al. (2020a)
Agro-food network	Different local material flows within the network (e.g. local fertilisation flow, animal requirements flow and energy flows), the average distance in exchanges of manure and grass and the CO_2_ emission from material transport.	Reuse, recycle and recovery	Multi-criteria assessment to compare the performances of different scenarios.	Fernandez-Mena et al. (2020b)
Hypothetical industrial symbiosis case studies comprising marble waste and concrete production, and alcohol slops used for fertiliser production	Percentage of waste exchange by each company involved in the IS, platform usage rate, and amount of saved residuals.	Reuse, recycle and recovery	n.a.	Fraccascia (2020)
A hypothetical marble-concrete industrial symbiosis case study	Economic performance indicator ( $\frac{\mathrm{economic}\;\mathrm{benefits}\;\mathrm{created}\;\mathrm{by}\;\mathrm{IS}}{\mathrm{production}\;\mathrm{costs}\;\mathrm{of}\;\mathrm{firms}}$ ), and environmental performance measure ( $\frac{\mathrm{total}\;\mathrm{waste}\;\mathrm{diverted}\;\mathrm{from}\;\mathrm{landfill}}{\begin{array}{c} primary\;inputs\;saved+total\;waste\\ produced\\ +required\;primary\;inputs \end{array}}$ ),	Reuse, recycle and recovery	n.a.	Fraccascia et al. (2020)
A hypothetical marble-concrete industrial symbiosis case study	Economic benefits, probability of implementation of industrial symbiosis.	Reuse, recycle and recovery	n.a.	Yazan and Fraccascia (2020)
Hypothetical industrial symbiosis case studies comprising marble waste and concrete production, and alcohol slops used for fertiliser production	Economic performance indicator = ( $\frac{\mathrm{economic}\;\mathrm{benefits}\;\mathrm{created}\;\mathrm{by}\;\mathrm{IS}}{\mathrm{production}\;\mathrm{costs}\;\mathrm{of}\;\mathrm{firms}}$ ), and environmental performance measure = ( $\frac{\mathrm{total}\;\mathrm{waste}\;\mathrm{diverted}\;\mathrm{from}\;\mathrm{landfill}}{\begin{array}{c} primary\;inputs\;saved+total\;waste\\ produced\\ +required\;primary\;inputs \end{array}}$	Reuse, recycle, recovery	Physical and monetary flows were modelled through EIOA.	Fraccascia and Yazan (2018)

n.a.: not applicable; TPB: theory of planned behaviour; GIS: geographic information system; EIOA: enterprise input–output analysis; ISN: industrial symbiotic network.

### Miscellaneous sectors

This section covers a range of studies which we were unable to allocate to any aforementioned sector. Details about the domain of application can be found in Tables 12 and 13.

**Table 12.** Case-study domains, quantified metrics, consideration of ‘R’ principles and use of other tools in system dynamics applications for miscellaneous sectors.

Case-study domain	Metrics and/or parameters quantified	Which ‘R’s were considered?	Use of other tools	References
Sustainable development of a city in Turkey	Annual gross domestic product (GDP) growth rate, social well-being, sustainable land use, CO_2_ emission, renewable energy production, technology innovation index, waste generation, and territorial competitiveness index.	Reuse, reduce, recycle, recover.	n.a.	Sezer et al. (2024)
Motor industry residues (waste oil, spent solvent, battery waste, and dirty wipes)	Outflows of waste oil, emissions (CO_2_), human toxicity, energy cost	Recycle, recovery	n.a.	Viruega Sevilla et al. (2022)
Aseptic paper packaging waste	Collection rate, recycling rate, extended producer responsibility fund, collection flow, recycling flow and waste in landfill	Recycle, repurpose	n.a.	Kuo et al. (2021)
Regional CE of Guangdong province in China	Mass of total and direct material input and output components, Resource consumption and waste emission (biological substance consumption, fossil fuel consumption, solid waste consumption), intensity efficiency index (total material input of 10,000 RMB of GDP, and total material output of 10,000 RMB of GDP), building material consumption and industrial exhaust emission	NA	n.a.	Gao et al. (2020)
A meso-scale implementation of a FPVC area in China	Benefits of livestock faeces recycling (e.g. biogas production rate, recycling amount of faeces, conversion amount of organic fertiliser, pollution free fruits and vegetables output), effects of water savings (e.g. water saving amount, annual recycling of wastewater, terrace area, total number of water cellar), effects of waste recycling (e.g. utilisation amount of potato residue, annual straw utilisation and burning, mulching fil remained, feeding beef cattle), effects of energy savings (e.g. fossil energy decreases, CO_2_ emissions reduction).	Reduce, reuse, recycle, repurpose, recovery	n.a.	Cheng et al. (2019)
Circular product systems	Environmental performance, cost-based economic performance and profit-based economic performance.	Remanufacturing, reuse, recycle	ABMS was coupled with system dynamics to capture the market information (population, income of the population, etc.) and offer attributes (price to offer, convenience of the offer, etc.)	Asif et al. (2016)

n.a.: not applicable; GDP: gross domestic product; ABMS: agent-based modelling and simulation; CE: circular economy; FPVC: fragility-economic poverty vicious cycles.

**Table 13.** Domains of case studies, quantified metrics, consideration of ‘R’ principles and use of other tools in agent-based modelling applications for miscellaneous sector.

Domain of case study	Metrics and/or parameters quantified	Which ‘R’s were considered?	Use of other tools	References
Onshore wind turbine blades circularity	Regional cumulative mean landfill rate according to different transportation costs, landfill behaviour under logistic barriers, adoption of thermoplastic blade design and the dissolution recycling pathway.	Reduce, reuse, repurpose, recycle	TPB was adopted to represent agents’ behaviour and its effects on the neighbouring agents.	Walzberg et al. (2022b)
Fashion renting process	Customers’ attitude towards fashion renting, performance of the service store and experience of the customer.	Reuse, refurbish, recovery	DES was used to model the fashion renting process.	Fani et al. (2022)

TPB: theory of planned behaviour; DES: discrete event simulation.

#### System dynamics modelling applications in miscellaneous sectors

Territorial competitiveness index (TCI) has emerged as a significant metric for gauging sustainable growth. Sezer et al. (2024) projected the development trajectory of Izmir in Turkey through TCI. In Viruega Sevilla et al. (2022), authors showed the system of waste oil flow in the motor industry and its circularity. Meanwhile, recognising the role of extended producer responsibility towards promoting CE, Kuo et al. (2021) developed a system dynamics model to determine the optimal subsidy (i.e. minimisation of cost and maximisation of recycling rate) between stakeholders pertaining to aseptic paper packaging. A parallel approach has been adopted to quantify and evaluate the implementation and effectiveness of regional CE (Gao et al., 2020). Tracking material and energy flows between industries considering the impact of environmental fragility-economic poverty vicious cycles were shown in Cheng et al. (2019). Additionally, Asif et al. (2016) developed an integrated ABMS and systems dynamics approach to understand the dynamic relationships between business approaches, supply chains and product design, along their influences on economic and environmental performance.

Causal loop diagrams outlined the causal interplay between sustainability indicators and TCI in Sezer et al. (2024). A similar diagram was utilised in Kuo et al. (2021) to represent the dynamics of waste generation, incorporating interventions related to production, costs, and recycling. When the multiple metrics for a particular value domain in CE is considered, there are always some cross-domain interconnectedness as presented in Asif et al. (2016) and Gao et al. (2020), where resource consumption subsystem, environmental impact subsystem, etc. were causally linked.

With the exception of Sezer et al. (2024), all studies in this sub-section developed stocks and flow diagrams to represent material flows. Sezer et al. (2024) used it for showing the accumulation of gross domestic product (GDP) growth, alongside material flows (i.e. waste generation). Temporal consideration was integral in all studies, leading to scenario analysis and recommendations through metrics quantification (see Table 12). For instance, Sezer et al. (2024) projected a peak of TCI in 2020, followed by a moderate decline to 2022, then a subsequent rise to 2027, due to development of sustainable policies, efficient resource utilisation, GDP increase, etc. Similarly, in Viruega Sevilla et al. (2022), authors used stocks and flow diagrams to show the flow of waste oil in the motor industry. While, Kuo et al. (2021) identified collection cost and recycler capacity were the most sensitive parameters in the system, highlighting the decreased collection costs increased extended producer responsibility (EPR) fund generation, and decreased recycler capacity led to increased landfilled waste. Gao et al. (2020) identified that, along with growth of CE, GDP also increased, contingent to slight decrease in birth rate and development of tertiary industries. Cheng et al. (2019) showed that CE improved the ecological and economic benefits in terms of improved livelihoods and reduced pollution of the considered system. For more information, see Supplemental Tables S18 and S19.

#### ABMS applications for miscellaneous sectors

ABMS has been adopted to examine circularity of wind power generation, considering end-of-life options for turbine blades (Walzberg et al., 2022b). Whereas, circularity via fashion renting, that is, a product-service system has been examined using ABMS to represent customers’ behaviour and interactions (Fani et al., 2022).

Both studies showed the heterogeneity of agents in terms of behaviour. This led to different paths for material circularity. With TPB used to represent the behaviours of the agents’, ABMS was crucial in representing how micro-level changes of the TPB parameters impacted the whole dynamics of the system. In each study, all of the agents were interconnected, and connected with the environment. Changes in any of them led to the emergence of new behavioural patterns to adapt them in the system. Spatial and temporal considerations were inherent, along with the stochastic natures of the agents. The quantified metrics are shown in Table 13. Furthermore, Walzberg et al. (2022b) found that regulatory pressure and attitudes positively impacted recycling, and agents were more prone to recycle when the recycling facilities were located close by. For more information, see Supplemental Tables S29–S31.

## Critical observations

This section examines a number of key themes highlighted by this study. It starts by examining the major themes considered by both system dynamics and ABMS; comparing and contrasting their application. It then discusses the metrics assessed by each of the modelling approaches, before considering the external tools used with each modelling approach. Finally, it considers which of the ‘R’-based frameworks are considered by each technique and in each sector. The section is not intended as a critical analysis of circularity, but rather of the modelling tools used for circularity quantification.

### Major themes of system dynamics and ABMS approaches

#### Why is system dynamics modelling favoured in CE quantification?

The preferences of system dynamics modelling in quantifying CE can be attributed to several key themes identified in the earlier discussions. These themes encompass causal loop diagrams, stock and flow diagrams, consideration of non-linearity or unpredictable variable evolution, temporal scale, spatial considerations, delay modelling, scenario generation and recommendations achieved through quantification of metrics.

A circular economy system is naturally complex, with lots of uncertainty and variability due to its constantly changing patterns. Thus, organisations seek an approach that can model systems and analyse their behaviour before actual implementation. System dynamics has both qualitative and quantitative analysis capabilities (Sumari et al., 2013). The qualitative capability is exemplified through causal loop diagrams (e.g. reinforcing and balancing) to account for the causal relationships between system variables. Quantification involves the transformation of causal loop diagrams into stocks and flow diagrams. The usual workflow for developing a system dynamics model is shown in Supplemental Figure S1. It shows that initial models often cannot capture reality, and system dynamics modelling is therefore an iterative approach, where the model is continually updated based on inputs from the system thinker. This was clearly stated by Sterman (2000).

The stock and flow diagrams in the literature are not only being used to represent the flow of materials but also to model flow of money, energy, policy variables, etc. The diagrams represent the aggregation and disaggregation of stocks and flows in a continuous time domain through various differential equations, accounting for the causality of other system variables. Thus, compared to dynamic material flow analysis (MFA) by system dynamics models, static MFA and Bayesian MFA do not allow extrapolation and exploration of future scenarios, but they rather provide snapshots of systems at a given time, and do not consider non-deterministic causality and/or interdependency of other system variables. However, this aggregation in a continuous time domain leads to loss of individual properties, and a perfect mixing condition becomes prevalent in terms of dynamic MFA (Guerrero et al., 2016). The (commercially) established/ standardised dynamic MFA performing platforms, often associated with life-cycle assessment approaches (e.g. STAN, Umberto and OpenLCA, SimaPro) do not consider the internal dynamics within the system.

Additionally, stocks and flows within any circular economy system (or complete value-chain) may be a stop-start process. For example, products may stay in a process, for example, use phase, for some period, causing delays. This delay modelling is a unique characteristic of system dynamics modelling and has been incorporated either stochastically or deterministically (Guzzo et al., 2021). This delay modelling can give rise to non-linearity, as can causal loops; where one parameter may have a non-linear relationship with other parameters, in turn affecting stocks and flows. Each of the sub-sections has shown examples of system dynamics being used to demonstrate changes over time.

While both ABMS and system dynamics can perform spatial analysis, the former requires more, finer system details than the latter. Conversely, the aggregation property of the latter provides decisions at a system level and may not provide finer details at the local level. While finer details may be possible, this comes at the cost of increased computational complexity.

#### Why is ABMS favoured in CE quantification?

The ABMS literature revealed major themes: heterogeneity, adaptation, agent-agent and agent-environment interactions (sometimes leading to emergent properties), spatial considerations, flow modelling, stochasticity of agents and scenarios analysis.

In terms of individuality modelling, ABMS is superior to system dynamics as it can handle finer details (Borshchev and Filippov, 2004). The memorising capability of ABMS leads to adaptation potential through path dependency. In other words, if agents are rewarded for following a particular path, then their future behaviour is more likely to also follow that particular path. This rewarding information is sometimes shared with neighbouring agents. Thus, discrete-time disaggregated agents interact with each other and/or their environment at each time step, and based on pre-defined state-chart rules, they either move to the next state or stay in the current state. The interaction space between agents is usually user-defined (e.g. circle; Fernandez-Mena et al., 2020b) and can be adjusted as per the case study (Guerrero et al., 2016). Furthermore, some interesting phenomena in the context of CE systems, such as, technology adoption, word-of-mouth and residents’ intention lead authors to ABMS. Although these can be modelled with a system dynamics approach, it is at the cost of time and complexity (i.e. by incorporating different variables and thereafter setting their values and causal relationships).

Another benefit of ABMS for simulating CE scenarios is its capability to incorporate the spatial scale. This has been attempted with a spatial system dynamics model (Neuwirth and Peck, 2013), (in different context) by integration of the geographic information system (GIS) system in the model, but its use is still not widespread. As shown in section ‘ABMS in industrial symbiosis’ and in Raimbault et al. (2020), the clustering of industrial symbiotic partners is highly relevant to circular economy systems at the meso- (regional) or macro- (national or global) scales.

The delay modelling in system dynamics and triggering of a particular event in ABMS are ostensibly analogous, but quite different in mechanism. While the former is part of the model’s internal process and is governed by mathematical equations, the latter signifies the activation of a particular agent, governed by rules. Furthermore, the former does not produce emergent properties, rather it influences the flows, while the latter may produce emergent properties but cannot be predicted in advance. Thus, depending on the rules, which are generally stochastic in nature, an event is triggered in the ABMS, while at the system level, properties are generated.

Conversely, ABMS can deal with qualitative data, which may be represented in terms of scale values. For instance, considering the technological yield increase probability and attention to social reasoning as discrete values between [0,1] (Farahbakhsh et al., 2023) and incorporating these in simulations and scenario analysis. Thus, when such parameters are not easily quantifiable, ABMS can be considered a suitable approach.

Scenario analysis in system dynamics and ABMS yield are both feasible, but from different perspectives. While system dynamics considers aggregate properties at the system level, in ABMS emergent properties are observed, which are in turn dependent on the agents and their dynamic interactions with each other and their environment. System dynamics scenario analysis often yields results from a single simulation run for a given parameter set, while ABMS typically requires multiple iterations to account for stochasticity and derive robust calculations. To elaborate, a single simulation run in system dynamics provides a complete picture of the system’s behaviour for a given set of initial conditions and parameters. However, the scenario analysis through system dynamics requires multiple parameters’ adjustments, which in turns require to re-run the model with the adjusted parameters to get the complete understanding of the system. Thus, each scenario typically involves a deterministic simulation unless uncertainty is explicitly incorporated. However, in ABMS, due to the stochasticity of the individual agents, and their associated rules, the system is itself stochastic. To understand the system’s behaviour, multiple iterations or simulation are required to account for the variability and derive statistical properties (e.g. confidence intervals).

#### Flow consideration through system dynamics and ABMS

**Figure 3.**
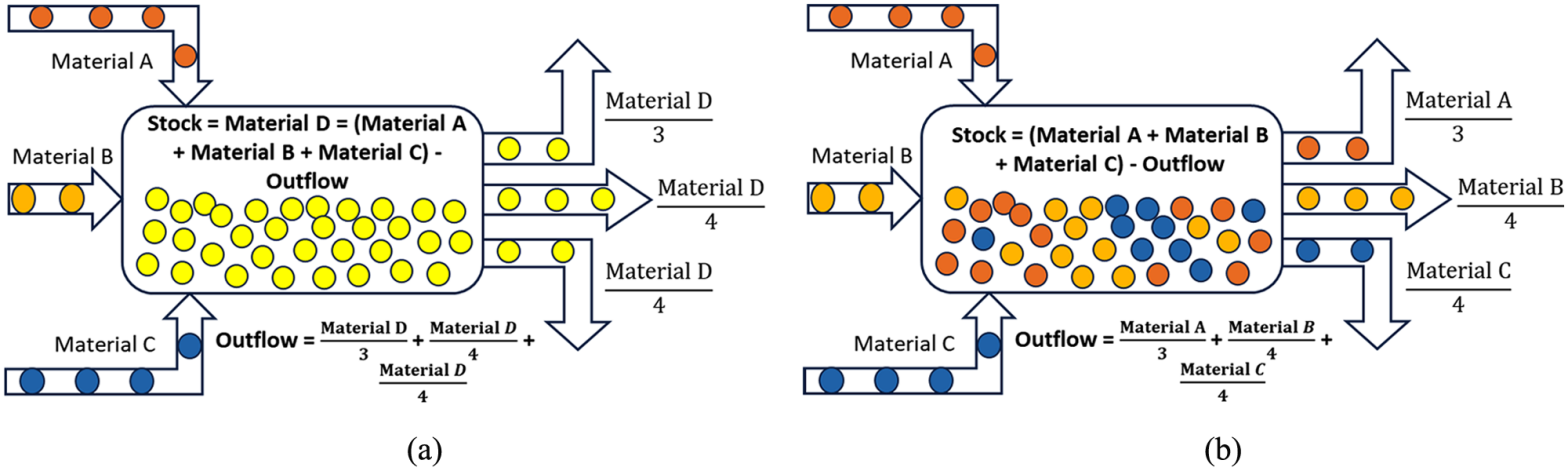
(a) Materials flows in system dynamics modelling approach; and (b) materials flow in ABMS approach. Materials identity is lost in SD models but retained in ABMS modelling, as indicated by the colour visualisation. SD: system dynamics; ABMS: agent-based modelling and simulation.

Flows are critical to revealing circularity, be they materials, energies, money, or policy. However, their modelling in ABMS and system dynamics models differ, as the former retains individuality, while it is lost in system dynamics, as shown in Figure 3(a) and (b) (considering the example of material flows).

Examining the papers in this study more closely highlighted a significant aspect. Boundaries conditions are a vital component of life cycle assessment, social life cycle assessment, and life cycle sustainability assessment. While CE modelling through system dynamics and/or ABMS can adopt proper steps to select system boundaries as per the cut-off criteria, as well as clear identification between elementary flows, reference flows, etc., these were absent from all the reviewed studies in this article. It should be imperative that the modeller identify and report the key aims and objectives of any CE system modelling. This will enable the proper modelling approach, plus coupling value metrics with flow modelling.

To explain further, consider the work of Walzberg et al. (2021), where agents were triggered in the material exchanges by economics or peer pressure. Additionally, at each time-step, the agent decided whether the waste material was landfilled or recycled, based on the cost-constraint. Similarly, in Raimbault et al. (2020), at each time step, each agent looked whether their waste outputs could be another agent’s input. They then considered spatial and economic perspectives (because increased inter-agent distances increased associated transportation costs). Only if these requirements were fulfilled, did material exchange take place. Conversely, material exchange in Fernandez-Mena et al. (2020b) was completely stochastic in nature, because the preference coefficient was not given as an input by the modeller but randomly selected by the simulator. Thus, ABMS modelling, considers material exchange both from the micro-level perspective, but also after meeting various criteria. While this can also be modelled by system dynamics models, it is at the cost of modelling complexity.

### Joint consideration of metrics through system dynamics and agent-based models

**Figure 4.**
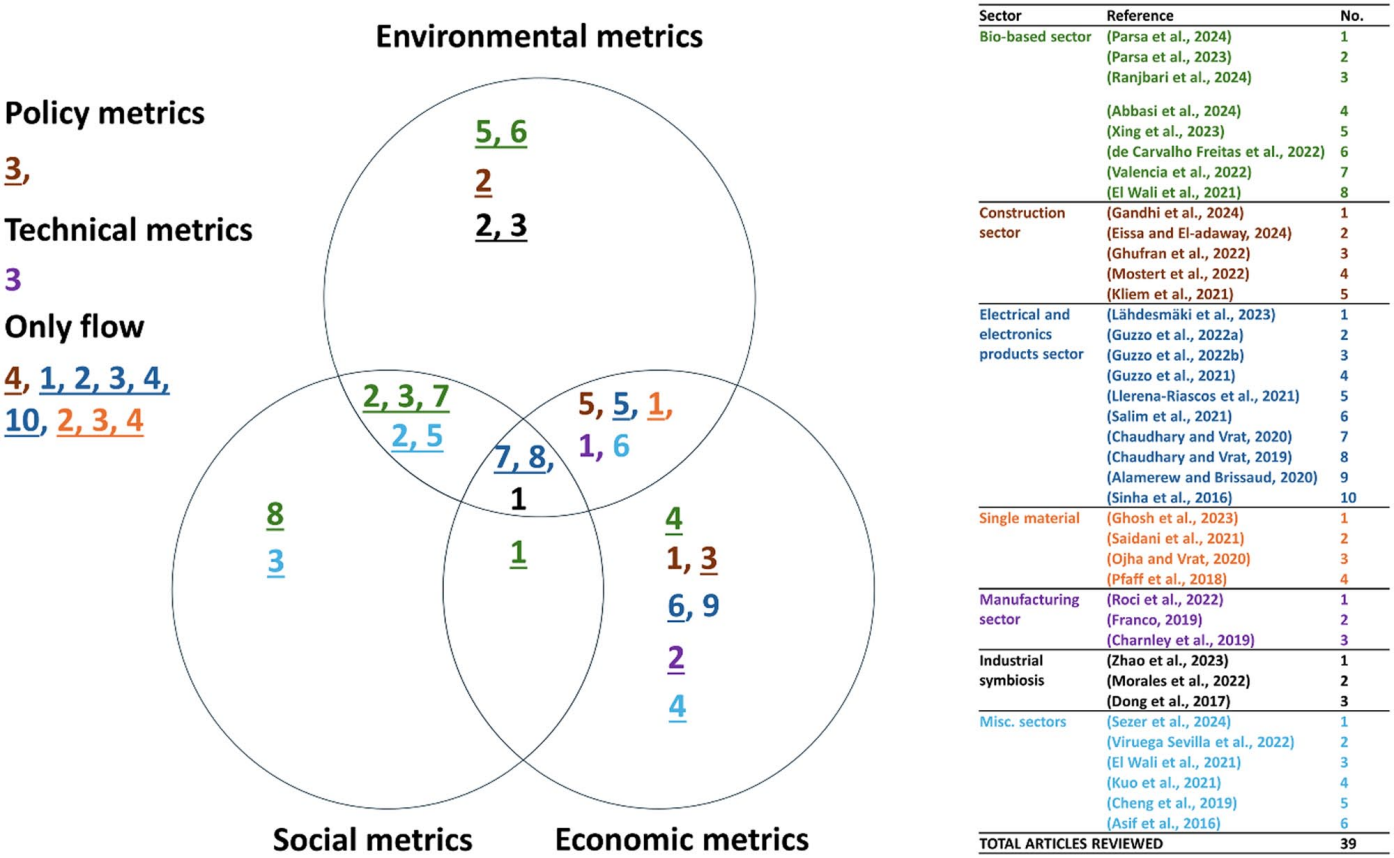
Consideration of different circularity metrics in system dynamics studies. The colours reflect the different sectors, while studies considering ‘flow’ are shown as underlined. Apart from the two studies in electrical and electronics products sector and one study in industrial symbiosis sector, none of the reviewed system dynamics studies jointly considered the quantification of environmental, economic and social metrics.

CE has been considered a practical approach to progress towards a sustainable future (Geissdoerfer et al., 2017; Kirchherr et al., 2017), adopting the three pillars of sustainability, as first proposed by Brundtland (1987) – economic gains, cleaner environment and societal prosperity. Figure 4 shows how various studies in the different sectors considered ‘value’-associated metrics from each of these three pillars. Half of the studies considered more than one metric, yet only around 10% considered value metrics covering all three of environmental, economic and social domains.

Overall, most studies focused on economic and then environmental aspects. However, there were differences between sectors. Studies in the bio-based sector had a greater emphasis on social and environmental metrics, while studies in the electrical and electronics sectors had a greater emphasis on economic aspects. Most of the studies considered flow metrics jointly with other metrics’ groups, but five studies from electrical and electronics products sector, three from single material sector and one from construction sector focused on quantification of the flow metrics only.

**Figure 5.**
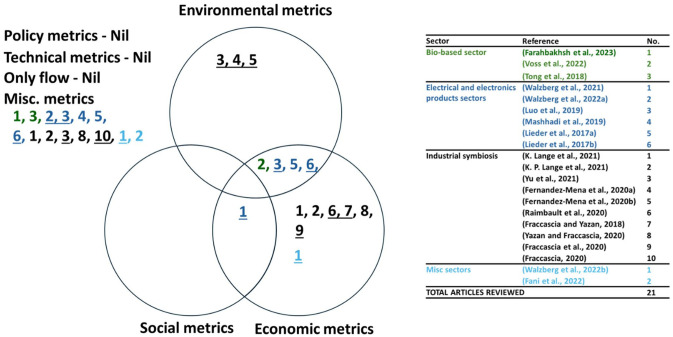
Consideration of different circularity metrics in ABMS studies. The colours reflect the different sectors, while studies considering MCP flow are underlined. None of the reviewed ABMS studies jointly considered the environmental, economic and social metrics. ABMS: agent-based modelling and simulation; MCP: material, components and product.

Similarly, the metrics studied by ABMS can be grouped into economic, environmental, social value, others, and MCPs flows (Figure 5). Focusing on environmental, economic and social value metrics, economic metrics dominated, followed by joint consideration of environmental and economic metrics, and environmental metrics. Meanwhile, only one study, in the electrical and electronic products sector, considered social aspects, in conjunction with economic metrics. While about half of the system dynamics studies considered multiple metrics, far fewer ABMS studies considered more than a single metric. Aside from studies in the electrical and electronic products sector only one study, in the bio-based sector, considered more than one metric. However, the presence of miscellaneous metrics not related to circular economy metrics become prominent in this group. Furthermore, none of the studies considered policy, technical or flow alone metrics.

The dominance of single-value metric studies using ABMS can be understood by recognising that this approach is mostly focused on how the changes in the micro-level agents’ behaviours impact the whole system. Possibly, researchers have avoided to jointly quantify the CE value metrics through ABMS due to increased computational complexity and traceability of models’ microstructures.

However, describing circular economy requires a holistic approach, where overall economic, environmental and social benefits must be quantified from the systems thinking perspective for informed decision-making. This enables targeted policy implementation through identification of key leverage points of the whole system. Furthermore, while analysis of individual systems has proved useful, these systems have still been somewhat isolated from a global perspective, and there have been limited endeavours to couple them and quantify metrics from a global perspective. This is possibly because there is, as yet, no global consensus on quantification of circular economy value metrics (Hossain et al., 2024; Mani et al., 2025; Negrete-Cardoso et al., 2022). Furthermore, value optimisation at key leverage points in the various systems considered here is missing.

### Utilisation of external tools along with system dynamics and ABMS

System dynamics can be considered as both rigid and flexible in its approach. It is rigid in terms of maintaining its fundamental principles as outlined in Supplemental Table S1, yet flexible in terms of its integration capability with other analytical tools. The inputs to the model can be optimised or tweaked, but there can be no changes in the basic principles of a system dynamics model. Meanwhile, outputs from system dynamics models can be further analysed using other tools, for example, for optimal CE scenario selection, plus cost and benefits of each process, or environmental impacts through life cycle analysis (LCA). In this context, DES has been used to quantify flows but requires more abstract knowledge of the system, which can be resource-intensive. This is possibly the reason why some studies resort to describing their models through hypothetical case studies.

ABMS can also be coupled with external tools. Many studies considered the TPB for modelling agents’ thought processes/behaviours, which influenced the state transition of the ABMS. It has also been adopted to incorporate regulatory behaviour and logistic constraints. Numerous studies have considered material flows in terms of enterprise input–output analysis, with metrics quantified through employing stochastic- and/or indicator-based ABMS. DES has been employed, but as mentioned earlier, it requires extensive abstract data, which is difficult to obtain. Additionally, data quality, uncertainty and selection of pertinent parameters have been considered in ABMS models.

Thus, both SD and ABMS are flexible in modelling the complexities of system thinking, by integrating various tools and techniques to quantify different aspects of circular economy, which in turn leads to more informed decision-making. However, this integration requires judicious thinking and case-specific challenges, which solely depends upon the modeller’s expert judgement.

### Where we are in CE tree? – ‘R’-Based Value Retention

**Figure 6.**
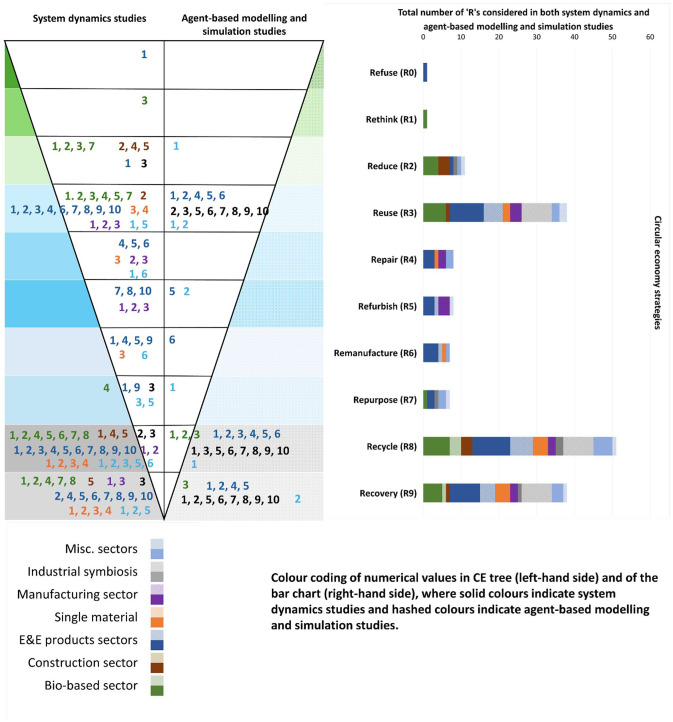
Prevalence of ‘R’-based strategies according to each of the sectors investigated in this study. Most of the studies are focusing at the lower end of a hypothetical CE hierarchy – that is, more extended effort cycles (i.e. recycle), where chances of value loss are perceived as being (substantially) higher. CE: circular economy.

In the introduction, the ‘R’-philosophies of CE frameworks were presented, with a more detailed elaboration available elsewhere (Jawahir and Bradley, 2016; Potting et al., 2017). Tables 3–13 show how the various studies considered each ‘R’ for each sector. Here, we discuss the aggregated view, as shown in Figure 6. Again, it has to be noted that we are reviewing the applications of system dynamics and ABMS to circularity, not research on circularity per se.

It has been argued that to maximise value retention of MCPs, focus should be on the upper part of the ‘R’-based CE-tree (‘R0’), instead of the lower part (‘R9’), where there are increased risks of value loss (Ellen MacArthur Foundation, 2013; Kirchherr et al., 2023). Our review demonstrates that this has not been the case to date when quantifying systems circularity: the overwhelming majority of studies reviewed here focus on recycle (R8) and recovery (R9), aside from some studies focused on reuse (R3). However, it should be noted that R2 (reduce), R3 (reuse), R8 (recycle) and R9 (recover) were the first four additions to the ‘R’ lexicon (Brennan et al., 2015; Yang et al., 2017) and hence have had longer to be considered within the literature. Conversely, refuse (R0) and rethink (R1) were each considered only once, despite being considered as key goals of a circular economy. However, these terms were some of the more recent additions to the ‘R’ lexicon (Kirchherr et al., 2017), and so some of the earlier studies may have predated their introduction. Also, much of the literature considers materials which have already entered the anthroposphere before circular economy ideas were popularised. Thus, the situation may change over the coming years as industry adopts circular design principles, incorporating durability, repairability and recyclability.

Additional information can be gleaned by considering which R philosophies have been considered in which sector. The bio-based sector (section ‘Bio-based Sector’) has focused on reduce, reuse, recycle and recovery. There has, perhaps obviously, been no consideration of repair, refurbish or remanufacture since the bio-based sector does not really concern itself with durable engineered products and these approaches are not applicable. Conversely, the products which are a focus of the manufacturing and electrical and electronic products sectors feature heavily from R3 (reuse) to R7 (repurpose). While there were not many studies focused on the construction sector, they considered reduce (R2), reuse (R3), recycle (R8) and recovery (R9).

Of these studies, three of the four which considered ‘R’ philosophies looked at demolition at either a system level (Eissa and El-adaway (2024) or at the material level (Gandhi et al., 2024; Kliem et al., 2021; Mostert et al., 2022). This reflects the status of the construction industry, with global production of concrete standing at about 30 billion tonnes per year (Purton, 2024), the overwhelming majority of which is used with very little consideration for circularity. This is placing increasing strain on the supply of high-quality aggregates (Kisku et al., 2017), with demand for sand trebling over the past two decades (Gallagher and Peduzzi, 2019). Furthermore, it must be remembered that the built environment typically has a long lifespan. Buildings coming to the ends of their lives now were designed and built maybe 50–80 years ago, certainly at a time predating interest in circularity as we perceive it today.

In addition, the rapid rate of modernisation and industrialisation has led to the generation of substantial quantities of debris originating from construction and demolition waste (CDW) (Abbas et al., 2006; Limbachiya et al., 2012). Over 700 million tonnes of CDW are produced each year (Soto-Paz et al., 2023), with estimates suggesting that, in the EU at least, about 35% is end-of-life concrete (Joseph et al., 2022). This is driving significant interest in the reuse of recycled concrete aggregate. This does not mean that there is no interest in reuse and repurposing, just that its benefits have not been quantified by SD or ABMS.

**Figure 7.**
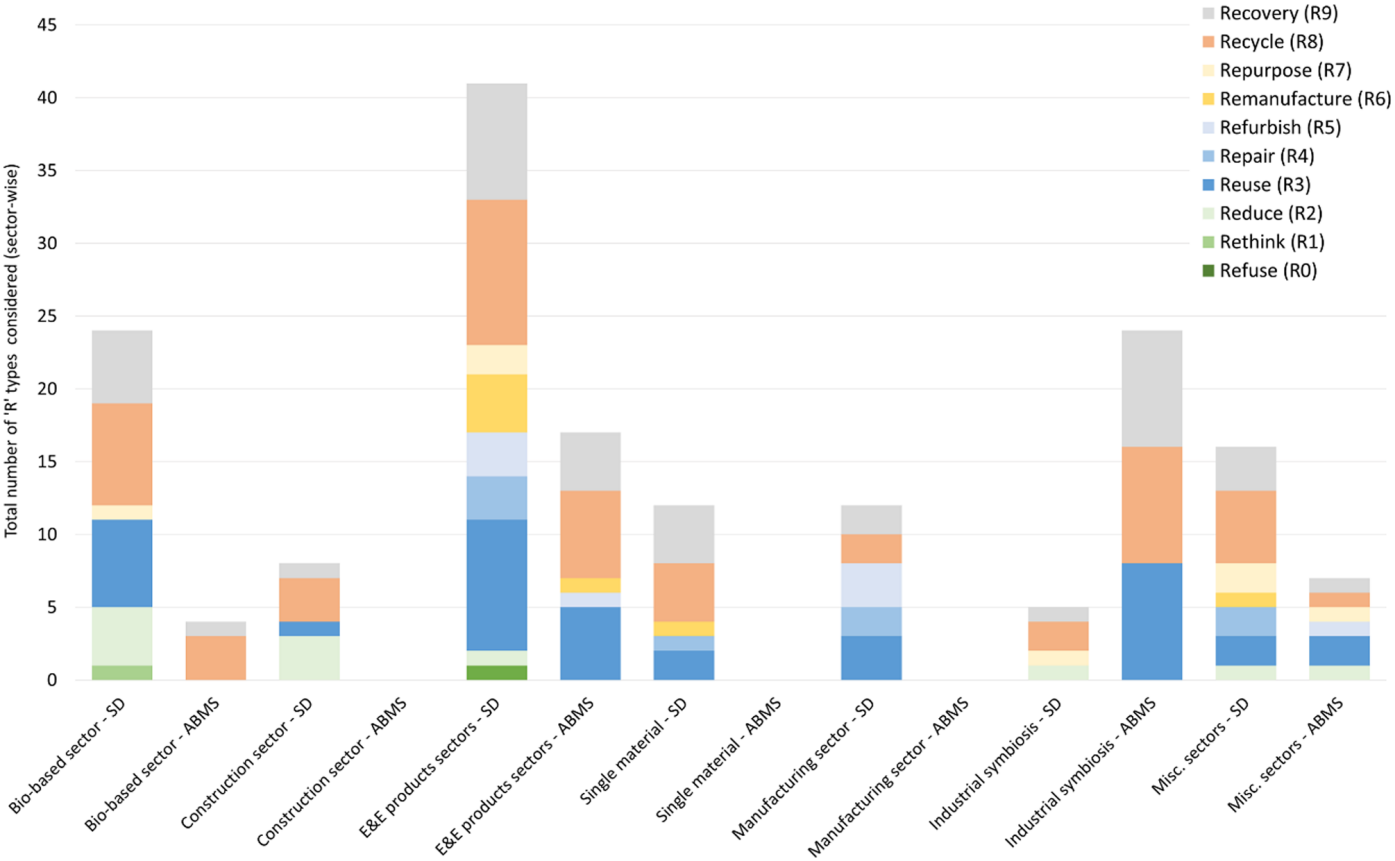
Total number of R-types (as stated in key) considered in studies pertaining to each sector, in adopting SD or ABMS approaches. SD: system dynamics; ABMS: agent-based modelling and simulation.

ABMS is a recent development compared to system dynamics. This has been evident throughout this study, with there being two to three times more papers on the latter compared to the former. This is confirmed by Figure 7, which groups the studies based on the sector. Indeed, industrial symbiosis is the only sector where studies using ABMS dominate. In this sector, the behaviour of individual agents is of key importance, hence the applicability of ABMS as an approach, with greater consideration of reuse, recycle and recovery. As seen with the studies adopting a systems dynamics approach, the focus in the bio-based sector is on recycling, reuse and recovery, with repair, refurbish and remanufacture not really applicable to the sector. In the construction sector, the focus is on reduce and recycle, reflecting perhaps greater awareness of the need to improve management of CDW arising from existing building stocks. While there is interest in reuse, repair, refurbish and repurpose within the construction industry, it is more at the design and construction stage than at end-of-life (European Committee for Standardization, 2025). Focus in the higher-value electrical and electronics products sector is on reuse, recycle and recovery options. Similarly, studies focusing on the manufacturing sector primarily considered reuse and refurbish, again reflecting the focus of the studies being products more than systems. The focus of the studies on single materials was recycle and recovery, reflecting the relatively high-value materials being considered in many of the studies. Finally, the miscellaneous sectors mostly focused on reuse, recycle and recovery options. Additionally, from the ABMS perspective, recycle was mostly widely considered, followed by reuse, and recovery. Interestingly, refuse, rethink and repair are missing from all studies.

## Future research directions

Based on the analysis and discussion, the following future research directions could be suggested, mapping with the research questions (RQs) we formulated initially:

RQ1: Need for holistic consideration for identification of leverage points in the circular value-chain – This paper started with a discussion of systems thinking and its associated complexity. The purpose of systems thinking is identifying root causes of challenges and solutions. In the CE context, this entails closing, slowing, or narrowing the materials and energy loops. The systematic literature review showed how previous literature has considered systems thinking, thereby identifying benefits of different CE-related metrics, and quantifying them. Various metrics have been considered in the value chain of the MCPs by using system dynamics and ABMS. However, information is still lacking along the whole value chain of MCPs from the raw material excavation to end-of-life processing, and reintroduction into the value chain. The values (i.e. economic, environmental, social and technical) generated/destroyed/transferred need to be considered at each process, and thereafter identifying the key leverage points (Iacovidou et al., 2017b; Millward-Hopkins et al., 2018). This will enable informed policy decisions, which will act to disrupt the current system, and in doing so reveal further leverage points, promoting further interventions, and so on in a continuous process (Boral et al., 2024). At this point, not only will the MCPs be flowing in a circular way, but so will the decisions and associated impacts. This cannot be visualised and quantified without the aid of simulation approaches. Furthermore, the review has highlighted the interdisciplinarity of circular economy research. This should be continued and encouraged for more informed circular economy decision making.
RQ2: Modelling scale and external shocks and interventions – Interventions from governments or policy makers, and external shocks (e.g. COVID pandemic) can distort, positively or negatively simulation outputs. The impacts of interventions cannot be quantified in a deterministic way, but the stochastic and emergent properties of ABMS can model this. Also, techniques such as Poisson process (where shocks are random and independent), renewal process (where shocks occur at constant rate), and Gamma process (shocks occur at a rate that increases with time) can model shocks. Each implementation software has its own benefits and limitations, with some more suited to system dynamics and others to ABMS. The ‘PySD’ package in Python has the capability to model the system dynamics architecture (Martin-Martinez et al., 2022), and the ‘Mesa’ package in the same platform can incorporate ABMS (Masad and Kazil, 2015). However, despite being open-sourced and freely available, to date, no study has explicitly demonstrated the integration of system dynamics and ABMS within Python-based software. This integration is particularly important because, in addition to the ‘PySD’ and ‘Mesa’ packages, Python offers a wide range of AI and machine learning libraries that can be incorporated into hybrid models (e.g. for imputing missing data through various ML algorithms). Furthermore, by utilising cloud computing resources, computational time can be significantly reduced – an advantage not typically available in other platforms that support SD–ABMS integration (e.g. AnyLogic). Without such integrated, scalable implementations, holistic modelling efforts will remain largely theoretical, thereby limiting their potential for widespread industrial adoption.
RQ3: Use of multiple simulation approaches for holistic consideration – Circular processes and associated decisions cannot be comprehensively modelled by a single simulation approach. Each approach has its advantages and drawbacks, as elaborated in detail in Supplemental sections S.3.2 and S.3.3. These simulation approaches are also sufficiently flexible to be integrated with other decision-making tools, typically environmental or sustainability-focused, enabling more informed decision-making, although the static nature on non-systems tools limits inherent compatibility. The integration of different tools and techniques requires experienced systems thinkers and analysts who are also conversant with circular economy and wider sustainability assessment.

Across the systems quantification, we observed a general state on data availability, uncertainty and quality. Circular economy research suffers from a lack of complete data in terms of materials, energy flows, etc. (Boral and Black, 2024; Lysaght et al., 2023; Velis et al., 2021; Wang et al., 2022, 2024). Moving across scales, from ‘micro’ level analysis to ‘meso’ or ‘macro’ level, another challenge is data aggregation, assuming data is available. Then, when system boundaries expand, modelling data uncertainty and quality are further challenges. Although fuzzy and Bayesian MFA are options to account for data uncertainty, they cannot handle the system dynamicity, and thus their application to holistic circular economy systems is still lacking. This can only be solved through open data and software platforms capable of multiple modelling approaches.

Although BS ISO 59004:2024 has recently proposed some overarching aspects of CE practice, along with how different stakeholders can benefit from that (The British Standards Institution, 2024a), it omits details of modelling complex CE systems, and thereby their quantification. Likewise, while the standard provides a list of CE metrics and states how value metrics are complex, the list of metrics inevitably cannot be comprehensive, and the standard does not consider how the metrics may change through a system’s inherent complexity. Our study is the first to highlight how different circularity metrics have been quantified using system dynamics and ABMS approaches. Overall, our PRISMA-ScR review provides insights into the strengths and limitations of these approaches, paving the way for further research in CE systems modelling.

## Conclusions

Systems thinking in the CE context refers to a holistic, interconnected approach that recognises economies, industries and societies as complex systems where all parts interact and influence one another. The application of systems thinking to circularity is not a new concept, but its application to quantify aspects of circularity is still at a nascent stage. Furthermore, systems thinking is a theoretical paradigm, but it remains unclear how it can be adopted to capture the complexities associated with CE system, and thereby quantify different metrics without them existing in isolation, but as interconnected entities. This review, based on a PRISMA-ScR framework, synthesised studies that used system dynamics and ABMS to capture CE complexities and demonstrate how abstract systems concepts can be operationalised. System dynamics provides a top-down, aggregated view through causal loops, stocks and flows and temporal dynamics, while ABMS enables a bottom-up representation of heterogeneity, adaptiveness, emergence and spatio-temporal interactions, which are particularly relevant to practical CE modelling. This latter approach is more recent and so is still developing. Consequently, the combination of system dynamics and ABMS has not yet been applied in a circular economy context; similarly limited are attempts to concurrently consider metrics from separate circularity domains of value – especially incorporating social aspects – despite the strong potential for enhanced circularity quantification such an approach could bring. Further combining system-level quantification with other sustainability-focused decision-support tools (well-established but currently mainly static) could enable more informed decision support on advancing circular economy theory and practice by industry and policymakers.

## Supplemental Material

sj-docx-1-wmr-10.1177_0734242X251413436 – Supplemental material for Conceptualising systems thinking and complexity modelling for circular economy quantification: A systematic review and critical analysis Supplemental material, sj-docx-1-wmr-10.1177_0734242X251413436 for Conceptualising systems thinking and complexity modelling for circular economy quantification: A systematic review and critical analysis by Soumava Boral, Leon Black and Costas A Velis in Waste Management & Research
