# Supplementary material for: Conceptualising systems thinking and complexity modelling for circular economy quantification: A systematic review and critical analysis
Source: Waste Manag Res. 2026 Feb 16;44(7):943–72. doi: 10.1177/0734242X251413436 (PMC13279986; doi:10.1177/0734242X251413436)
Supplement: sj-docx-1-wmr-10.1177_0734242X251413436 – Supplemental material for Conceptualising systems thinking and complexity modelling for circular economy quantification: A systematic review and critical analysis [file sj-docx-1-wmr-10.1177_0734242X251413436.docx]

***Supplementary Material***

***for***

**Conceptualizing systems thinking and complexity modelling for circular economy quantification: A systematic review and critical analysis**

Soumava Boral^1^, Leon Black^1^, Costas A. Velis^1,2^ *

^1^ School of Civil Engineering, University of Leeds, LS2 9JT, Leeds, UK

^2^ Department of Civil and Environmental Engineering, Imperial College London, SW7 2AZ, London, UK

*Corresponding author: [c.velis@imperial.ac.uk](mailto:c.velis@imperial.ac.uk)

# **Supplementary Information Overview**

The following information are presented here:

1. Modelling complexities in systems thinking by system dynamics and agent-based modelling and simulation **(S.2)**,
2. Systems thinking in circular economy **(S.3)**
3. PRISMA-ScR checklist **(S.4)**,
4. Methods including choices on the application of the systematic review, Boolean search queries, inclusion exclusion criteria, selection of sources and data charting (**S.5**, **S.6**, **S.7**),
5. Descriptions of the key points, detailed answers to the research questions, and other information related to applications of system dynamics modelling in circular economy **(S.8)**,
6. Descriptions of the key points, detailed answers to the research questions, and other information related to applications of agent-based modelling and simulation approach in circular economy **(S.9**),
7. Definitions of different ‘R’s used in CE literature (**S.10**).

# **Definitions of Different R’s Used in Circular Economy Literature**

In this section, we reproduce the definitions of R’s as presented in BS ISO 59004:2024 (The British Standards Institution, 2024):

- **Refuse (R0):** Refusing referrers to re-evaluating resource use patterns to ensure that organisations pursue sufficiency and are set up to prevent overusing resources, Refusing can make a solution(s) redundant by demonstrating to an organisation that it is not required, or its function can be replaced by a solution with the same function or with radically different solution.
- **Rethink (R1):** Rethinking refers to a reconsideration of design and manufacturing decisions with a different mindset (e.g. making service use more intensive, sharing or by putting multi-functional products on the market). Rethink solutions should be easily repaired, maintained, refurbished, remanufactured, upgraded or reused.
- **Reduce (R2):** Reducing demand for a product helps in lowering the associated resource use. This strategy can also include reduction with the intent to increase efficiency in product manufacturing or by consuming fewer natural resources.
- **Re-use (R3):** Re-use a discarded product which is still in working condition and fulfils its original function. Reusing products or components that a user no longer needs for the same function for which they were originally used over multiple usage cycles is an important measure for reducing resource use and losses and increasing value capture from existing products.
- **Repair (R4):** Restore a defective or damaged product so that it can be used in its original function. If a product malfunctions or breaks it cannot be suitable for (re)use and risks being discarded. To avoid that, preventive or predictive maintenance is necessary. To enable maintenance and repair, the availability of spare parts, maintenance and repair instructions as well as services are essential.
- **Refurbish (R5):** Restore to a useful condition during expected service life with similar quality and performance characteristics. Refurbishing can include activities such as repair, rework, replacement of worn parts, and update of software or hardware, but does not include activities that result in the need for a new product certification and a legal manufacturer status of the refurbisher. Refurbishing does not include restoration after the expected lifetime.
- **Remanufacture (R6):** Return an item, through an industrial process, to a like-new condition from both a quality and performance perspective. By remanufacturing products, components or parts, a company contributes to the circular economy by extending the lifetime of those elements and thus creates additional value for the organisation, the customer, the workers, and the environment. Remanufactured products or parts can often come with a warranty equivalent to the warranty the product received when new.
- **Repurpose (R7):** Adapt a product or its parts for use in a different function that it was originally intended without making major modifications to its physical or chemical structure.
- **Recycle (R8):** Recover and process material to obtain the same (high grade) or lower (low grade) quality through activities such as recovery, collection, transport, sorting, cleaning and re-processing. Recycling can involve a mechanical, physical, chemical process or biological processes, or a combination of these processes. Consideration should be given to how much energy is used for the recycling process.
- **Recover (R9):** In a circular economy, waste is minimised by intention. However, waste will still be created throughout the transition towards a circular economy and needs proper management and treatment. Within the circular economy, products or components that have insufficient value for the holder are given a temporary waste status in some countries because of regulatory requirements. Nevertheless, they can become a recovered resource if there is an active recovery process in place. Otherwise, they can intentionally be removed from the system through landfilling or incineration. In the case of incineration with energy recovery, resources are effectively removed from the system, but their energy content is recovered.

Energy recovery is an end-of-life operation deployed to collect and generate heat or power from a resource that has surpassed its ability to flow through the circular economy. Energy recovery is most effective when coupled with an end-of-life resource recovery process, such as anaerobic digestion, which created conditions to capture nutrients and produce and agricultural input while generating energy. Other energy recovery processes, such as combined heat and power, can produce ash or sludge as a by-product that can be used as an input for yet another recovery process. A residual material input that feeds into the creation of a new fossil-based fuel is also primarily an energy recovery practice.

Whereas material recovery is the method of recapturing and reutilising recoverable resources specifically for reuse, refurbishing, remanufacturing, recycling or other methods that add or retain value of a resource.

# **Modelling Complexities in Systems Thinking by System Dynamics and Agent-Based Modelling and Simulation**

This section describes the key concepts related to complexities associated with systems thinking and how they can be modelled through system dynamics and agent-based modelling and simulation (ABMS). Interested readers are directed to (Sterman, 2000) and (Railsback and Grimm, 2019) for detailed explanations of system dynamics and ABMS, respectively.

## **Systems Thinking: A Brief Introduction**

A system is *“an interconnected set of elements that is coherently organised in a way that achieves something”* (Ellen MacArthur Foundation, 2019). Systems thinking is supposed to be critical in handling the complexities of the future world, but it still sits at the educational margins (Arnold and Wade, 2015). Richmond, who proposed the term *‘systems thinking’,* said *“as interdependency increases, we must learn to learn in a new way. It’s not good enough simply to get smarter and smarter about our particular “piece of the rock”. We must have a common language and framework for sharing our specialised knowledge, expertise and experience with “local experts” from other parts of the web. We need systems Esperanto. Only then will we be equipped to act responsibly”* (Richmond, 1994).

Systems thinking consists of three things: elements, interconnections, and a function or purpose (Arnold and Wade, 2015). However, to present the *‘function’* or *‘purpose’* of the system, and to model the system from a holistic (or *‘collaborative’* as mentioned in (Kopainsky et al., 2011) point of view, feedback, emergence, delays, stocks and flows, non-linearity, boundaries and perspective, and dynamicity over time have to be considered (Sweeney and Sterman, 2000). Only then it will provide individuals and organizations deeper insights into the behaviour and dynamics of complex systems, enables informed policy as well as decisions making to address challenges and create positive change. This was also argued by Arnold and Wade while they tried to find the missingness in the research of systems thinking: *“…answer to the elusive concept of systems thinking in a way that will allow it to be measured”* (Arnold and Wade, 2015).

## **System Dynamics Model: Key Characteristics**

The term model is defined as *“an abstract and simplified representation of a given reality, either already existing or just planned. Models are commonly defined in order to study and explain observed phenomenon or to foresee future phenomenon”* (Bandini et al., 2009). System dynamics models enable us to analyse the dynamic non-linear relationships and time-dependent pattern changes of complex systems. It has roots in the theory of non-linear dynamics and feedback control frequently used in mathematics, physics, and engineering. It aims to understand different scenarios generated from complex systems, optimising outcomes according to (or constrained by) necessities.

It provides a set of concepts, principles, and methodologies for modelling, simulating, and analysing the dynamic behaviour of systems (Sterman, 2000). System dynamics theory has been widely applied to various domains, including economics, management, engineering, environmental science, public policy, and social systems. The flow-chart for developing a system dynamics model is shown in Figure S1, whereas Table S1 provides a checklist for developing an system dynamics model.

System dynamics theory considers the complexity of systems thinking by employing feedback loops, stocks, and flows diagrams (SFDs), time delays, nonlinear relationships, and causal loop diagrams (CLDs), while adopting an iterative approach(Sterman, 2000)

However, the system dynamics theory is not free from criticism, such as listed below:

1. It excludes interactions between different agents (Farahbakhsh et al., 2023). For the sake of simplicity, although they are present, the modeller has to exclude them.
2. The solutions provided by system dynamics models can be sub-optimal. The approach also struggles to deal with imprecise or subjective information (Xu et al., 2009).
3. A successful system dynamics model should include as few as possible exogenous variables (Sterman, 2000) (e.g., policy recommendations, external impacts, etc.), which may not be the actual scenario.


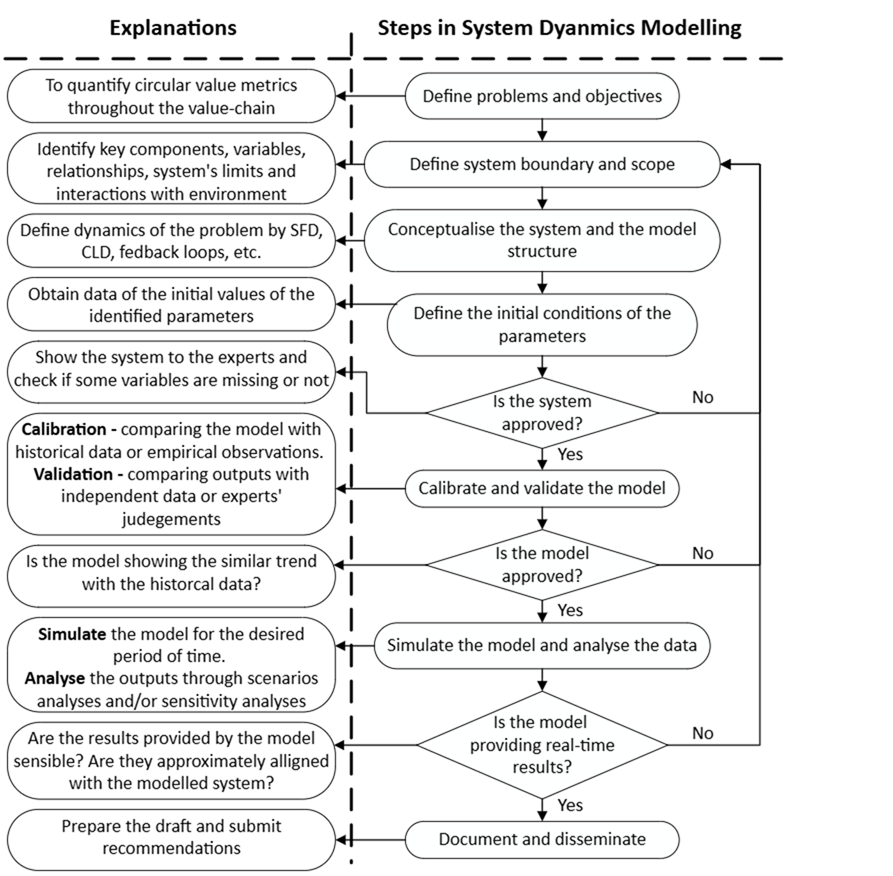


Figure S1. Steps involved in developing a system dynamics model.

Table S1. Checklists for developing a system dynamics model (Sterman, 2000).

| **Sections and sub-sections** | **Questions to be addressed** | **Responses** | **Where is it located?** |  |
| --- | --- | --- | --- | --- |
| **Problem articulation (boundary selection)** | | | | |
| Selection of theme | What is the problem? |  |  |  |
|  | Why it is a problem? |  |  |  |
| Key variables | What are the key variables and concepts the modeller must consider? |  |  |  |
| Time horizon | How far in the future the modeller must consider? |  |  |  |
|  | How far back in the past lie the roots of the problem? |  |  |  |
| Dynamic problem definition (reference modes^[[1]](#footnote-2)^) | What are the historical concepts and behaviours of the key variables and concepts? |  |  |  |
|  | What might be their behaviour in future? |  |  |  |
| **Formulation of dynamic hypothesis**^[[2]](#footnote-3)^ | | | | |
| Initial hypothesis generation | What are the current theories of the problematic behaviour? |  |  |  |
| Endogenous^[[3]](#footnote-4)^ focus | Formulate a dynamic hypothesis that explains the dynamics as endogenous consequences of the feedback structure. |  |  |  |
| Mapping | Develop maps of causal structure based on initial hypotheses, key variables, reference modes, and other available data, using the tools like model boundary diagrams^[[4]](#footnote-5)^, subsystem diagrams^[[5]](#footnote-6)^, causal loop diagrams^[[6]](#footnote-7)^, stock and flow maps^[[7]](#footnote-8)^, policy structure diagrams, or other facilitation tools |  |  |  |
| **Formulation of a simulation model** | | | | |
| Specification | Specify the structure and decision rules |  |  |  |
| Estimation | Estimate the parameters, behavioural relationships, and initial conditions |  |  |  |
| Tests | Test for the consistency with the purpose and boundary. |  |  |  |
| **Testing** | | | | |
| Comparison to reference modes | Does the model reproduce the problem behaviour adequately for your purpose? |  |  |  |
| Robustness under extreme conditions | Does the model behave realistically when stressed by extreme conditions? |  |  |  |
| Sensitivity | How does model behave under uncertainty in parameters, initial conditions, model boundary and aggregations? |  |  |  |
| **Policy design and evaluation** | | | | |
| Scenario specification | What environmental conditions might arise? |  |  |  |
| Policy design | What new decision rules, strategies, and structures might be tried in the real world? How can they be represented in the model? |  |  |  |
| What-if analysis | What are the effects of the policies? |  |  |  |
| Sensitivity analysis | How robust are the policy recommendations under different scenarios and given uncertainties? |  |  |  |
| Interactions of policies? | Do the policies interact? Are there synergies or compensatory responses? |  |  |  |

## **Agent-Based Modelling and Simulation: Key Characteristics**

Following the definition of a model in the previous sub-section, the term *‘simulation’* refers to *“the usage of a computational model to gain additional insights into a complex system’s behaviour by envisioning the implications of the modelling choices, but also to evaluate designs and plans without actually bringing them into existence in the real world”* (Bandini et al., 2009). Agent-based modelling and simulation (ABMS) is a computational technique used to simulate complex systems composed of autonomous entities called agents. Each agent has its own behaviour, decision-making rules, and interactions with other agents and the environment. This approach has been widely adopted for its ability to model complex human-environmental systems by capturing the dynamics and heterogeneity of individual actors. The steps involved in developing an ABMS are shown **Figure S2**.

Unlike system dynamics modelling, ABMS is a bottom-up approach (**Table S2**), starting from micro-level agents, and then proceeding towards the macro-level emergent patterns. The emergent patterns cannot be anticipated initially because they arise from complex interactions between agents, as well as agents with the environment. Furthermore, the agents in the ABMS are heterogeneous (viz., distinct characteristics, attributes, behaviours, and decision-making rules). This modelling approach is useful for modelling the non-linear dynamics of the system. Another important characteristic of the ABMS is agents here are stochastic in nature, and stochasticity is incorporated at the agent level. In ABMS, sensitivity analyses can be performed at both agent and systems levels, but in system dynamics theory it is only visible at the systems level.

Table S2. Steps involved in developing an agent-based model.

| **Steps No.** | **Steps tasks** | **Steps descriptions** |
| --- | --- | --- |
| 1 | Define the problem | Define problem statement(s), scope(s), and objective(s). |
| 2 | Define agents, system boundary, and their attributes | Identification of agents, their characteristics, attributes, and other relevant properties. |
| 3 | Create the environment | Define environment conditions, resources, and any other pertinent factors of the environment, within which the system of consideration belong. |
| 4 | Specify agents’ behaviours | Define agents’ rules, behaviours, reactions, movements, and their interactions in-between them and with the environment. |
| 5 | Implement the model | Implement the model in any suitable software. |
| 6 | Initialise the simulation | Define agents’ initial state, time-step for simulation, and duration of simulation. |
| 7 | Run the simulation | Run the simulation and note down the agents’ evolution over each time-step if possible. |
| 8 | Collect and analyse data | Collect data of agents’ trajectories, other outcomes, and system level metrics |
| 9 | Validate and calibrate | Validate the model by comparing the results with historical data. If necessary, calibrate the model. |
| 10 | Perform the sensitivity analyses | Perform sensitivity analyses by varying the key endogenous and exogeneous variables. |
| 11 | Perform scenario analyses | Perform scenario analyses through assuming the paths that system might follow. |
| 12 | Visualisation, reporting, and documentation | Visualise, report, and provide recommendation. |

In this context, note that ABMS has often been interchangeably used with complex adaptive system (CAS), although they are not synonymous. CAS is a conceptual framework that describes a class of complex systems characterized by emergent behaviour, self-organization, and adaptation. While ABMS is a specific modelling technique within the CAS framework that focuses on simulating the behaviour and interactions of individual agents to understand the emergent properties of the system (Nilsson and Darley, 2006).

Despite multiple benefits of ABMS, it has been criticised for multiple reasons, such as computational complexity, calibration and validation challenges, data requirements, simplification and abstraction of the model, model interpretation and transparency, sensitivity to the parameters, and limited predictive power.

To summarise, systems thinking provides the overarching framework for understanding complex systems, whereas system dynamics and ABMS offer specific methodologies and tools to study and simulate system’s behaviour. System dynamics and ABMS are not mutually exclusive and can complement each other. System dynamics models can capture the aggregate behaviour and feedback dynamics of a system, while agent-based models can capture the heterogeneity, individual-level behaviours, and interactions. Combining these approaches offers a deeper understanding of complex systems, examining both macro-level dynamics and micro-level interactions within a system.

# **Systems Thinking in Circular Economy**

In their report about systems thinking in circular economy concept, Ellen MacArthur Foundation emphasised that *“we have a microscope for understanding the infinitely small and the telescope for the infinitely great. Perhaps we need a macroscope for understanding the infinitely complex?”* (Ellen MacArthur Foundation, 2019). However, systems thinking has been conceptualised as sole system dynamics approach, where subsystems have been modelled feedback loops, stocks and flows, and interconnections (Barnabè and Nazir, 2022; Demartini et al., 2022; Fiksel et al., 2014). Through systems thinking approach, novel innovations, policy interventions, new operating practices, etc. can be put forward rather solely relying on various *‘R’*—based principles (Fiksel et al., 2014). While, Morales et al. (Morales et al., 2022) in their work considered systemic perspective in light of the complexity theory, and the authors argued that forecasting should be a critical perspective of systems thinking.

However, there are several schools of thought who have visualised CE differently, rather than just ‘decoupling’ growth from resource use (Reike et al., 2018). For example, some scholars expanded the discussions of industrial ecology to CE by increasing the system boundary, and thus incorporating stakeholders, supply chain perspectives, and resource conservation through systems thinking approach (Bocken et al., 2017). These scholars also argued that in systems thinking, rather than considering the one-dimensional flows within system boundaries, a combined thinking considering business models, materials, and products (and thus organisational aspects rather than technical matters) should be proposed. Another school of thought considered CE as a practical implementation tool for sustainability, and thus combining systems thinking as an approach for maximising the environmental, social and economic values (Lieder and Rashid, 2016). The list below highlights how systems thinking can benefit understanding circular economy:

1. Holistic perspective: It provides a holistic perspective so that stakeholders can better understand the interdependencies between different sectors, industries, and value chains.
2. Feedback loops and causal relationships: It examines feedback loops and dynamic behaviour, which are crucial in driving resource flows, waste reduction, and the adoption of circular practices. By identifying and understanding these feedback loops, stakeholders can develop strategies to reinforce positive feedback loops (e.g., increasing demand for recycled materials) and address negative feedback loops (e.g., reducing waste generation) (Roci et al., 2022).
3. Leverage points of the system: It identifies leverage points that, when targeted, can significantly impact on the overall system behaviour. Thus, stakeholders can identify and prioritize interventions with the greatest potential for transformative change (Walzberg et al., 2022a). By mapping and analysing the structure, relationships, and flows within a system, systems thinking can identify inefficiencies, bottlenecks, or areas where resources are lost or wasted. Thus, stakeholders can identify opportunities for resource optimization, closing material loops, and designing more circular business models.
4. Stakeholders’ collaborations: Systems thinking encourages collaboration and stakeholder engagement across different sectors and disciplines. The circular economy requires the active involvement and cooperation of various stakeholders, including policymakers, businesses, consumers, and waste management systems. Systems thinking helps foster a shared understanding of the complex challenges and facilitate collaborative efforts to overcome barriers and drive systemic change.
5. Long-term analysis: Systems thinking promotes a long-term perspective and resilience in the circular economy. Stakeholders thus consider the long-term consequences of decisions and actions, anticipate potential unintended consequences, and design strategies that ensure the longevity and viability of circular systems.

Applying systems thinking principles aids holistic understanding. This can inform the design of more effective policies, business models, and interventions to promote the transition to a more sustainable and circular economy.

# **PRISMA-ScR Checklist**

Table S.3. Preferred Reporting Items for Systematic reviews and Meta-Analyses extension for Scoping Reviews (PRISMA-ScR); checklist; after (Tricco et al., 2018).

| **Section** | **Item** | **PRISMA-ScR Checklist Item** | **Where reported in this study** |
| --- | --- | --- | --- |
| **Title** | | |  |
| Title | 1 | Identify the report as a scoping review | Front page |
| **Abstract** |  |  |  |
| Structured summary | 2 | Provide a structured summary that includes (as applicable): background, objectives, eligibility criteria, sources of evidence, charting methods, results, and conclusions that relate to the review questions and objectives. | Section 1 |
| **Introduction** |  |  |  |
| Rationale | 3 | Describe the rationale for the review in the context of what is already known. Explain why the review questions/objectives lend themselves to a scoping review approach. | Section 1 |
| Objectives | 4 | Provide an explicit statement of the questions and objectives being addressed with reference to their key elements (e.g., population of participants, concepts, and context) or other relevant key elements used to conceptualize the review questions and/or objectives. | Section 1 |
| **Methods** |  |  |  |
| Protocol and registration | 5 | Indicate whether a review protocol exists; state if and where it can be accesses (e.g., a Web address); and if available, provide registration information, including the registration number. | Not applicable (optional) |
| Eligibility criteria | 6 | Specify characteristics of sources of evidence used as eligibility criteria (e.g., years considered, language, publication status), and provide a rationale. | Section S.5 |
| Information sources* | 7 | Describe all information sources in the search (e.g., database with dates of coverage and contact with authors to identify additional sources), as well as the date the most recent search was executed. | Section S.5.1, and S.5.2 |
| Search | 8 | Present the full electronic search strategy for at least 1 database, including any limits used, such that it could be repeated. | Section S.5 |
| Selection of sources of evidence† | 9 | State the process for selecting sources of evidence (i.e., screening and eligibility) included in the scoping review | Section S.6 |
| Data charting process‡ | 10 | Describe the methods of charting data from the included sources of evidence (e.g., calibrated forms or forms that have been tested by the team before their use, and whether data charting was done independently or in duplicate) and any processes for obtaining and confirming data from investigators. | Section S.7 |
| Data items | 11 | List and define all variables for which data were sought and any assumptions and simplifications made. | Section S.6 |
| Critical appraisal of individual sources of evidence∆ | 12 | If done, provide a rationale for conducting a critical appraisal of included sources of evidence; describe the methods used and how this information was used in any data synthesis (if  appropriate). | Not applicable (optional) |
| Synthesis of results | 13 | Describe the methods of handling and summarizing the data that were charted. | Section S.7 |
| **Results** |  |  |  |
| Selection of sources of evidence | 14 | Give numbers of sources of evidence screened, assessed for eligibility, and included in the review, with reasons for exclusions at each stage, ideally using a flow diagram. | Section S.7 |
| Characteristics of sources of evidence | 15 | For each source of evidence, present characteristics for which data were charted and provide the citations. | Section S.7 |
| Critical appraisal within sources of evidence | 16 | If done, present data on critical appraisal of included sources of evidence (see item 12). | Section 3 |
| Results of individual sources of evidence | 17 | For each included source of evidence, present the relevant data that were charted that relate to the review questions and objectives. | Section 3 |
| Synthesis of results | 18 | Summarize and/or present the charting results as they relate to the review questions and objectives. | Section 4 |
| **Discussion** |  |  |  |
| Summary of evidence | 19 | Summarize the main results (including an overview of concepts, themes, and types of evidence available), link to the review questions and objectives, and consider the relevance to key groups. | Section 4 |
| Limitations | 20 | Discuss the limitations of the scoping review process. | Section 6 |
| Conclusions | 21 | Provide a general interpretation of the results with respect to the review questions and objectives, as well as potential implications and/or next steps. | Section 6 |
| Funding | 22 | Describe sources of funding for the included sources of evidence, as well as sources of funding for  the scoping review. Describe the role of the funders of the scoping review. | Section 7 |

PRISMA-ScR = Preferred Reporting Items for Systematic reviews and Meta-Analyses extension for Scoping Reviews.

* Where *sources of evidence* (see second footnote) are compiled from, such as bibliographic databases, social media platforms, and Web sites.

† A more inclusive/heterogeneous term used to account for the different types of evidence or data sources (e.g., quantitative and/or qualitative research, expert opinion, and policy documents) that may be eligible in a scoping review as opposed to only studies. This is not to be confused with *information sources* (see first footnote).

‡ The frameworks by Arksey and O’Malley (6) and Levac and colleagues (7) and the Joanna Briggs Institute guidance (4, 5) refer to the process of data extraction in a scoping review as data charting*.*

∆ The process of systematically examining research evidence to assess its validity, results, and relevance before using it to inform a decision. This term is used for items 12 and 19 instead of "risk of bias" (which is more applicable to systematic reviews of interventions) to include and acknowledge the various sources of evidence that may be used in a scoping review (e.g., quantitative and/or qualitative research, expert opinion, and policy document).

# **Methods**

## **Boolean search queries used in finding the literature for system dynamics applications in circular economy**

TITLE-ABS-KEY

( ( Circular Economy"  OR  "Circularity"  OR  "Circular" )

AND

("System Dynamics" OR "System Dynamics Theory") )

## **Boolean search queries used in finding the literature for agent-based modelling and simulation applications in circular economy**

TITLE-ABS-KEY

( ( Complex adaptive system"  OR  "Agent-based modelling"  OR  "Agent-based"  OR  "Agent based" )

AND

("Circular economy" OR "Circularity" OR "Circular") )

# **Inclusion and exclusion criteria**

## **Inclusion and exclusion criteria for system dynamics modelling in circular economy**

The three-stage screening process for selecting the pertinent literature related to circular economy and system dynamics modelling is shown in **Table S4**.

Table S 4. Criteria for selecting the pertinent literature related to applications of system dynamics modelling and circular economy.

| **Section** | **Detail** | **Included** | **Excluded** |
| --- | --- | --- | --- |
| Title of the research | Relevance and scope | - Mentioned the term(s) ‘circular economy’ and/or ‘system dynamics’. - Mentioned any *‘R’* of the circular economy. - Mentioned any terms which leads to circular economy (e.g., industrial symbiosis) - Mentioned the term ‘simulation’. | - Totally out-of-context title or research field (e.g., titles related to physics domain). - Concepts not relevant to circular economy. - Focused on directly policy analysis. |
|  | Study type | - Full length peer reviewed journal article - Case study - Methodology development | - Review article - Conference articles - Conference review - Book chapter |
|  | Languages | English | All other languages other than English |
|  | Temporal type | Articles published after 2016 | Published before 2016 |
| Abstract of the research | Relevance and scope | Have mentioned any of the terms: system dynamics, causal-loop diagram, stock and flow diagram, dynamic complexity, systems thinking. | Have specifically mentioned only the development of causal loop diagram but not stock and flow diagram. |
| After going through the full research text | Relevance and scope | - Developed the material flow/mass flow diagrams through stock and flow diagrams. - Quantified different value metrics emphasising on stock and flow diagrams. - Able to answer the research questions | - Did not show the mass flow/material flow through stock and flow diagrams. - Only developed causal loop diagram but not stock and flow diagrams. - Not able to answer the research questions |

## **Inclusion and exclusion criteria for applications of agent-based modelling and simulation in circular economy**

The three-stage screening process for selecting the pertinent literature related to circular economy and agent-based modelling and simulation is shown in **Table S5**.

Table S5. Criteria for selecting the pertinent literature related to applications of agent-based modelling and circular economy.

| **Section** | **Detail** | **Included** | **Excluded** |
| --- | --- | --- | --- |
| Title of the research | Relevance and scope | - Mentioned the term(s) circular economy and agent-based modelling or complex adaptive system - Mentioned any ‘R’ of the circular economy concept. - Mentioned any terms which leads to CE (e.g., industrial symbiosis) - Mentioned the term ‘Simulation’ | - Totally out of the context title or research field (e.g., titles related to physics domain) - Concepts not relevant to circular economy - Focused on directly policy analysis |
|  | Study type | - Full length peer reviewed journal article - Case study - Methodology development | - Review article - Conference articles - Conference review - Book chapter |
|  | Languages | English | All other languages other than English |
|  | Temporal type | Articles published after 2016 | Published before 2016 |
| Abstract of the research | Relevance and scope | Mentioned any of the terms: ABM, complexity theory, circular economy or any other terms related to circular economy | Did not present any data or not validated the approach through real-time and/or hypothetical data |
| After going through the full research work | Relevance and scope | - Showed complexity diagrams - How complexity arose and how this impacted the whole system - Able to answer the research questions | - Did not show any complexity diagram - Not able to answer the research questions |

# **Selection of Sources and Data Charting**

## **Selection of literature pertaining to systems dynamics applications in circular economy**

Please refer Figure 1.a. of the main article.

## **Selection of literature pertaining to ABMS applications in circular economy**

Please refer Figure 1.b. of the main article.

# **Descriptions of the Key Points, Detailed Answers to the Research Questions, and Other Information Related to Applications of System Dynamics Modelling in Circular Economy**

## **Bio-based sector**

Table S6. Elaborated descriptions of the key points and research questions pertaining to literatures of system dynamics applications in circular economy – bio-based sector.

| **Source** | **Key points** | **RQ-1: What are the contexts/necessities of applying system dynamics (circularity special focus)** | **RQ – 2: Which types of decisions were made through system dynamics model?** | **RQ -3: Any other tool(s)/method(s) used in conjunction with system dynamics approach? Why?** | **Location of the study** | **Software used** |
| --- | --- | --- | --- | --- | --- | --- |
| (Parsa et al., 2024) | - The socio-economic impacts of food-waste were investigated. - Food waste reduction in consumer sector and redistribution in supply sectors were favourable from overall environment, social and economic benefits. - For retail sector, both reduction and redistribution options were highly favourable. | - To capture the dynamics of socio-economic factors. - To map the flow of food and waste along the value-chain. - To develop scenarios and forecast the benefits from 2018-2030. | - Food waste reduction and redistribution of surplus food were key for achieving sustainable society. | No | United Kingdom | Stella |
| (Parsa et al., 2023) | - Challenged the static food waste hierarchy model. - Proposed an optimised model for food waste reduction in food, energy, water and climate (FEWC) nexus. - Food surplus redistribution in supply sector and reducing food waste generation at the consumer level were most critical factors impacting the food footprints. | - To capture the causal dynamics between different sectors, as well as flow of food and waste along the value-chain. - To generate and examine the impacts of different scenarios on the system. | - Food waste reduction at the consumer level and redistribution level were suggested. | Yes  Group model building was incorporated to consider the decisions and opinions of stakeholders at different levels. | United Kingdom | Stella |
| (Ranjbari et al., 2024) | - The adoption of food-sharing platform to reduce the food waste was explored. - Circularity indicator in terms of food waste prevention was quantified from systems thinking perspective. | - To understand and quantify the whole dynamics of the food-sharing app platform. - How population accepts the food sharing platform based on their knowledge and marketing force. | - The marketing force was sufficient in dealing with food waste. - Knowledge enhancement program between adults and younger generation yet to be improved which linked them to adoption of food-sharing app. | Yes  Bass diffusion model was adopted to model the customers willingness to adopt online food-sharing platform app. | Italy | Vensim |
| (Abbasi et al., 2024) | - A framework consisting of small and medium-scale farmers and their sustainable development through chicken farming was examined. - The forward and reverse value-chain were depicted, and different metrics were quantified through stocks and flows diagram. - The collaboration between different stakeholders were examined and interlinkages were pointed out. | - To develop the forward and reverse value chain model. The forward value-chain encompassed the material flows, and reverse one consisted of cash flow and waste valorisation of poultry waste. | - There are strong interlinkages between forward and reverse value chain, which led to the food security landscape of the chicken system. | No | Malaysia | Vensim |
| (Xing et al., 2023) | - A framework for reactive nitrogen (Nr) flow, emission, and mitigation strategy was proposed by integrating reactive nitrogen flow model and system dynamics approach. - The system dynamics model was adopted to predict the Nr flow in regional food system under three mitigation strategies: balanced diet of residents, minimized food loss waste and a recycling food system. | - System dynamics facilitated the modelling of the interaction between mitigation measures of Nr emissions with a forecasting between a period of 2000-2019. | - Authors observed that recycling food system scenario eventually decreased the environmental Nr emission. - The integration of material flow analysis with system dynamics aided authors to understand the sustainable food system in the considered region. | No | Fujian, southeast China | Vensim PLE |
| (de Carvalho Freitas et al., 2022) | - Investigated the potential of waste cooking oil as a source of second-generation biofuel for the timeframe between 2020-2030. - The supply chain was visualized by the system dynamics model. | - Causal loop diagram was used to represent the influence of different variables for waste oil production and biofuel production. - Stock and flow diagram was developed from causal loop diagram to represent different scenarios and the progression of different environmental parameters for the considered simulation period. | - The reuse and recycling option of waste cooking oil through sustainable scenario confirmed the minimization of waste and increase resource and energy efficiency. - Reducing the import of diesel, the proposed framework confirmed the reduction of fossil fuel import and penalties for green-house gas emission. | No | Brazil | Vensim PLE |
| (Viruega Sevilla et al., 2022) | - Only the flow of used oil in the waste treatment plant was considered for modelling through system dynamics approach. - Material recovery of the waste oil through sener interline process was considered. | - The system dynamics was adopted to develop different scenarios and thereafter to forecast the flows and stocks dynamics of waste oil management system. | - Flows of different materials and by-products were forecasted along with energy cost incurred by the plant, demand, and savings. | No | Spain | Vensim PLE |
| (Valencia et al., 2022) | - Developed the system dynamics model to describe the material and energy flows across the food, energy, water and waste (FEWW) sectors aiming to reach the urban sustainability transition. - The stock and flow diagram was used to highlight the resource interdependencies and interconnectedness between different sectors. - Some key circularity aspects considered were reduction in raw material consumption through storm water reuse, reuse of food waste for compost production, waste to energy, etc. - Apart from circularity indicators different sustainability and resilience indicators were also considered and examined through system dynamics approach in this study. | - Initially system dynamics was used to describe the flow of materials and waste inside the system boundary. - Later, system dynamics was used to compute and carbon footprint and waste footprint, directly coupled with flow of materials. | - System dynamics was used to portray the FEWW nexus in a region of USA. - System dynamics helped the authors in producing the material flow analysis happening inside the system boundary for the period of 12 months, and thereafter values of different indicators were computed (related to circularity, sustainability, and resilience). | Yes  - Cost benefit-risk trade off was presented for the considered sectors (e.g., urban agriculture, coal energy used, utilization of natural gas and solar PV, production, and consumption of landfill gas, etc.)  - MCDM method (viz., TOPSIS) was used to evaluate the four scenarios with respect to sustainability and resilience indicators. | United States | Stella |
| (El Wali et al., 2021) | - Authors used the system dynamics approach in conjunction with material flow analysis and social life-cycle assessment (SLCA) to address the social challenges involved in global phosphorus supply chain, and thereafter closing its loop through CE concept. - Using system dynamics, this study analyses the social impact of phosphorus circularity in the context of the sustainable development goals. - For multiple variables, historical data were used to estimate those values (sometimes following a 3-parameter Weibull distribution). - However, authors did not provide information on the causal loop diagram and stock and flow diagram, or how from the causal loop diagram, the stock and flow diagram was drawn. | - SD modelling was used to study the behaviour of complex (many objects interact with one another), interactive (variables' interaction produces the distinctive behaviour of the system) and dynamic (distinctive behaviour of the system varies over time) systems within changeable conditions. - The SD model was simulated till 2010-2050. - CE indicators were calculated including the macro view of 163 countries. | - The results showed that the circular economy affects phosphorus flows by different patterns of behaviour for each region including balancing and reinforcing social issues in long-term perspective until 2050. - Detailed analysis shows that the circular economy contributes to global improvement in the P security for all regions, except Northern Africa where linear economy model offers higher security. - The system dynamics model was aimed to provide results on different SDG goals: such as SDG 1, SDG 2, SDG 3, SDG 5, SDG 8, SDG 12 | Yes   - Considered system dynamics in conjunction with material flow analysis and SLCA. - The significance of this combination lies in the ability to quantify mass flows and assess social consequences within a holistic system considering the dynamic behaviour and interaction of multiple parameters of global phosphorus supply chain over time. - SLCA was used to determine the social impact of linear and circular phosphorus as input for system dynamics model. | Global | AnyLogic |

Table S7. Reasons for developing causal loop diagrams (CLDs), stock and flow diagrams (SFDs), and descriptions of scenarios for system dynamics modelling in circular economy applications – bio-based sector.

| **References** | **Causal loop diagram(s) developed?** | **For which purposes causal loop diagram(s) was/were developed?** | **Stock and flow diagram(s) developed?** | **For which purposes Stock and flow diagram(s) was/were developed?** | **Scenarios developed?** | **What were the scenarios?** |
| --- | --- | --- | --- | --- | --- | --- |
| (Parsa et al., 2024) | No | N/A | Yes | To describe the flow of materials between different sectors, and their cost-benefit dynamics evolution, and evolution of social benefits | Yes | Several scenarios on food surplus and waste management options, as well as food surplus redistribution options |
| (Parsa et al., 2023) | No | N/A | Yes, but shown through Sankey diagram | To describe the flow of materials (or food, and its waste) along the value chain, as well as their footprints. | Yes | Different scenarios and sub-scenarios were developed by varying the parameters related to food waste production, policy variables, consumption pattern, etc. |
| (Ranjbari et al., 2024) | Yes | To depict the causality between multiple variables | Yes | To show the co-flow between knowledge enhancement among adults and younger generations, adoption of food sharing platform, familiarity of food sharing platform and circular economy indicators evolution between 2015 – 2060. | Yes | Different scenarios were developed based on the marketing performance enhancement, extra knowledge enhancing efforts for adults and children, etc. |
| (Abbasi et al., 2024) | Yes | To depict the operational dynamics withing the indigenous chicken value chain. | Yes | To describe the dynamic nature of the chicken, egg, revenue, and waste flow inside the system. | Yes | Varied the number of chickens lay egg, transported to the slaughterhouse, etc. related to the material flow, and observed the system level impact. |
| (Xing et al., 2023) | Yes | To show the reactive nitrogen flow with recycling food system. | No. | The material flow was depicted in terms of Sankey Diagram. | Yes | Balanced diet, minimized food loss and waste, recycling food system. |
| (de Carvalho Freitas et al., 2022) | Yes | To show the influence between waste oil generation and biofuel production. | Yes | To present the flow of material. | Yes | Baseline, non-sustainable and sustainable. |
| (Valencia et al., 2022) | No | N/A | Yes | To represent the flow of materials, carbon footprint and water footprint analyses. | Yes | Baseline scenario, expansion of urban agriculture, increase in solar PV farms, stormwater reuse and recycling. |
| (Viruega Sevilla et al., 2022) | No | N/A | Yes | To model the flow of waste oil. | Yes | Waste oil not treated, sener interline process, sener interline process with treatment process implanted progressively, diesel oil product used as a fuel. |
| (El Wali et al., 2021) | No | N/A | Yes | To depict the phosphorus mass flow in its supply chain and social impacts associated with mass flow. | Yes | Linear economy scenario, circular economy scenario. |

**N/A:** not applicable

## **Construction sector**

Table S8. Elaborated descriptions of the key points and research questions pertaining to literatures of system dynamics applications in circular economy – construction sector.

| **References** | **Key points** | **RQ-1: What are the contexts/necessities of applying system dynamics (circularity special focus)** | **RQ – 2: Which types of decisions were made through system dynamics model?** | **RQ -3: Any other tool(s)/method(s) used in conjunction with system dynamics approach? Why?** | **Location of the study** | **Software used** |
| --- | --- | --- | --- | --- | --- | --- |
| (Gandhi et al., 2024) | - Estimated and forecasted cost of five different grades of recycled paver block using system dynamics approach. | - The interdependencies between 25 cost components were explored by causal loop and stock and flow diagrams. - The dynamic interactions between cost components were carried out. - To carry out scenarios analyses by varying multiple variables. | - Contractor profit and overhead expenses, as well as labour costs were the most influencing cost components in the dynamic interactions. | No. | India | Vensim |
| (Eissa and El-adaway, 2024) | - Quantification of the estimates of the embodied carbon emissions (projected growth of floorspace) to the US commercial building stocks and decarbonisation potential of different circular economy policies. | - Forecasting the embodied carbon emissions and impacts of circular economy principles on decarbonisation. - Creating detailed co-flow structures. | - To describe the interactions and dynamics of different variables associated with new floorspace, demolished floorspace, embodied emissions, and floorspace concrete emissions. - To perform scenario analyses. | No | United States | Not mentioned |
| (Ghufran et al., 2022) | - Identified the key enablers of CE for the sustainable development of construction industry and mapped their causal dependencies by causal loop diagram. - The system dynamics model was developed to address the complexity of the sustainable development of CE in construction industry. | - The causal loop diagram was developed to identify the relationships between the enablers of CE. - Quantification and forecasting of different enablers, such as organizational incentive schemes, policy support, sustainable development through stock and flow diagram. | - Aided the authors to reach a conclusion that policy supports, and incentive schemes play a pivotal role for the sustainable CE of construction industry. | No | Global participants | Vensim PLE |
| (Mostert et al., 2022) | - Developed a dynamic model for calculating the flow of building materials in the German construction sector in future, considering the potential reuse of recycled aggregates. - With the help of system dynamics, the future inflows, and outflows of residential buildings in Germany were forecasted with an assumed construction and deconstruction rate. | - System dynamics was used to estimate the resource conservation potential of the deconstruction materials. - Different scenarios were generated and tested to examine the resource conservation potential by reusing the recycled aggregates. | - A forecasting was carried out for the period of 2020-2060 regarding the utilization of recycled aggregates, saved sand and gravels and unused recycled aggregates for developed scenarios. | No | Germany | Powersim Studio |
| (Kliem et al., 2021) | - Developed a system dynamics model with relevant actors from public policy and industries to identify the barriers for utilizing the secondary mineral resources and the feasibility of different policies for closing the loop of the mineral-based construction materials. - Authors used socio-technical transition literature to combine technical, social, and historical perspective to understand the transition dynamics. To understand the actors from the private and public institutions, they used participatory system dynamics modeling. - Authors also tried to understand the feedback mechanism of construction material industry and governing institutions in case of exogeneous pressures. - Finally, in this work, authors identified the structures of the dynamics that was inhibiting the transition to sustainable resource management of excavation materials and construction and demolition waste (CDW). | - The stock-and-flow diagram was used to highlight the structure of the regional coupled extraction and disposal services and demand for recycled aggregates. It consisted of multiple inputs, endogenous policies, exogeneous policy variables. - Through modeling the problem by system dynamics approach, the aim of the work was to identify the governance feedback that formed the barriers to recycling of CDW in Switzerland and proposed policy levers that supported the transition to circularity. | - The study offered an operational perspective on transitions, where causality shaped system boundaries and endogenous barriers to transitions were identified by eliciting institutional decision rules. - Apart from other authors found that reducing the delay between developing and deploying alternative materials was a critical issue for circular material flows in resource intense economies. | No | Switzerland | Vensim PLE |

Table S9. Reasons for developing causal loop diagrams (CLDs), stock and flow diagrams (SFDs), and descriptions of scenarios for system dynamics modelling in circular economy applications – construction sector.

| **References** | **Causal loop diagram(s) developed?** | **For which purposes causal loop diagram(s) was/were developed?** | **Stock and flow diagram(s) developed?** | **For which purposes Stock and flow diagram(s) was/were developed?** | **Scenarios developed?** | **What were the scenarios?** |
| --- | --- | --- | --- | --- | --- | --- |
| (Gandhi et al., 2024) | Yes | To depict the interdependencies between different cost components. | Yes | To show the temporal evolution of total manufacturing cost based on the causal interdependencies of other cost variables. | Yes | It was performed by multivariate sensitivity analysis, where constant parameters values were varied between -50% to +50% of the uniform distribution for all four grades (M30, M35, M40, M45) of RPB. |
| (Eissa and El-adaway, 2024) | No | N/A | Yes | To describe the flows associated with new floorspace development, demolished floorspace, embodied emissions, and floorspace concrete emissions. | Yes | Building embodied carbon intensity reduction policy, unit size reduction policy, landfill diversion policy, adaptive reuse policy, concrete embodied carbon intensity reduction policy. |
| (Ghufran et al., 2022) | Yes | To identify the causal links between the enablers of CE in construction industry. | Yes | Organizational incentive schemes, policy support and sustainable development were chosen as stocks because they were influenced by inflows and outflows. | No. | N/A |
| (Mostert et al., 2022) | No | N/A | Yes | To compute the resource conservation potential by replacing the natural aggregates with recycled aggregates. | Yes | Recycling scenario, secondary input scenario, and resource saving scenario. |
| (Kliem et al., 2021) | Yes | To examine the public policy instruments impacts on the business model. | Yes | To model the sand and gravel quarry and disposal volumes. | Yes | Multiple policy scenarios, such as landscaping, lighthouse, resource constraint etc. |

N/A: not applicable

## **Electrical and electronics products sector**

Table S 10. Elaborated descriptions of the key points and research questions pertaining to literatures of system dynamics applications in circular economy – electrical and electronics products sector

| **References** | **Key points** | **RQ-1: What are the contexts/necessities of applying system dynamics (circularity special focus)** | **RQ – 2: Which types of decisions were made through system dynamics model?** | **RQ -3: Any other tool(s)/method(s) used in conjunction with system dynamics approach? Why?** | **Location of the study** | **Software used** |
| --- | --- | --- | --- | --- | --- | --- |
| (Lähdesmäki et al., 2023) | - Global flow of lithium was modelled through dynamic material flow analysis and considering the impact of different factors. - The usage, waste, and accumulation of lithium in different sectors were forecasted. | - To capture the dynamics of lithium flow in global scale. - To perform the scenarios analyses. | - Lithium might be in extinction by 2050 if the combined circular economy strategy is not followed. - Large amount of lithium is being wasted in extraction and refining phase as mine tailings, followed by EV batteries. | No. | Global study | Vensim |
| (Guzzo et al., 2022b) | - The developed system dynamics model tried to examine whether the interventions regarding collection of end-of-life products (electrical and electronics equipment) were according to the Brazilian Industrial Agreement for Electrical and Electronic Equipment (BIAEEE). - Twelve policy scenarios were developed including increase in collection coverage, spread of the collection points and rewards to facilitate the study. | - The system dynamics was implemented for designing different scenarios, and thereby examining their impacts. - The developed models and sub-models were used to capture the dynamics of demands of smartphones, their flows in the economy and waste generation and collection strategies. | - It helped the decision makers to reach in a decision on how to maximize the circularity of the waste electrical and electronic equipment by examining different scenarios. | No | Brazil | Vensim DSS |
| (Guzzo et al., 2022a) | - Proposed and tested a system dynamic based framework for examining the CE transition to support decision-making at micro, meso and macro levels. - Two case-studies related to healthcare industry (micro-level and bottom-up change) and EEE industry (macro-level/conceptual change) were used for the inductive theory building, and then one case-study pertaining to the EEE industry (macro-level/top-down approach) was solved by the deductive theory building. | - System dynamics was used to represent the flow of materials inside the system boundary. | - To examine the consequences of different policy scenarios in the CE transition for a prolonged period. | No. | Not mentioned | Vensim DSS |
| (Guzzo et al., 2021) | - The purpose of the model was to represent the long-term nationwide dynamics of EEE stocks and lows when introducing specific interventions for CE strategies implementation. - The scope of analysis is the long-term (1980 - 2050) use of specific EEEs, such as flat display panel TVs and refrigerators, nationwide. - Bass diffusion model was used to represent the adoption of technology. | - Authors developed the circular EEE system dynamics model, analogous to the dynamic MFA, which aimed to assess future stocks and flows of resources in the anthroposphere through scenarios generation based on past information. - The system dynamics model comprised of four sub-models: a) technology adoption, b) EEE obsolescence, c) EEE and WEEE flows, and d) material supply. - The EEE and WEEE flows represents first use, second use, remanufacturing, recycling, as well as WEEE disposal. The Weibull distribution was used for failure rate modeling. | - Six critical stock and flows for the eight defined scenarios were analysed and forecasted, namely availability of raw material, material extraction, EEE commissioning, total EEE in use, disposal of EEE as WEEE, WEEE recycled. | Yes   - Bass diffusion model was coupled with SD to model the diffusion of technology in the system. | Netherland | Vensim DSS |
| (Llerena-Riascos et al., 2021) | - Proposed an integrated optimisation-based simulation model, based on system dynamics theory to design a sustainable WEEE management system policies. - Optimisation-based system dynamics framework was used to represent the complexity of the WEEE generation processes. - The main objective of the paper was to highlight the incapability of sole system dynamics approach to capture the operative-strategic nature of the system and perform the optimal parameter updates, which is also known as policy optimisation in system dynamics nomenclature. | - The system dynamics models were developed for two things: (a) one for WEEE generation process, where system dynamics was used alone, and (b) WEEE management process, where system dynamics was coupled with mixed-integer non-linear programming (MINLP) optimisation process. - Circularity was considered in a vague manner, and a fraction of WEEE was considered for recycling. - Few environmental benefits, along with some economic benefits were simulated for the period of 2018-2037 on a weekly basis. - Different scenarios were generated and examined through system dynamics modeling approach. | - System dynamics coupled with optimisation process were able to maximize the profits from the WEEE management process as well as minimisation of environmental burdens. - system dynamics was aimed to identify the key actors in the WEEE management process as well as to examine the effectiveness of different policies on the profit and other economic indicators maximisation and environmental burden minimisation. | Yes  Mixed integer non-linear programming approach was used in combination with system dynamics to find out the optimal values of different SD parameters to increase the efficiency of the system dynamics model. | Colombia | Not mentioned |
| (Salim et al., 2021) | - Developed a participatory system dynamics model for managing end-of-life rooftop photo-voltaic (PV) panels based on CE concept. - Authors followed a qualitative-quantitative triangulation method to search for a consensus on the mental models among the stakeholders. - Interview transcripts were coded by using the grounded theory approach to identify the key themes around the end-of-life management of residential PV panels and battery energy storage system (BESS). - Workshops were conducted with the stakeholders to confirm the system boundary, causal relationships, and system archetypes. - Expert judgment was used in case of data unavailability. | - Causal loop diagram was adopted to map the dynamic hypothesis and information and material delays of the system under study. - Stock and flow diagram was derived from the causal loop diagram, which was segmented into three parts – market, policies and regulations and collection and recovery. - Scenario analyses were carried out. | - Different scenarios were analysed for forecasting multiple parameters such as collection rate, collection fraction, landfill, payback period, etc. - System dynamics was adopted to analyse the different socio-technical transition pathways toward the effective management of end-of-life of residential PV cells. - Shared responsibility revealed a balanced outcome in terms of collection and recovery rate, as well as effect on the payback period. | Yes  Qualitative-quantitative triangulation method was adopted to describe the mental model of the stakeholders. | Australia | Vensim DSS |
| (Alamerew and Brissaud, 2020) | - Authors tried to explore the dynamics of environmental, economic, and social aspects from a reverse supply chain perspective. - System dynamics was adopted to model the dynamics of cost, revenue, and strategic and regulatory decisions. - Enablers and barriers of end-of-life electric vehicle batteries (EVB) were identified. | - System dynamics was applied to model the interplay between the design, business model, reverse supply chain, product/service use, policy and end-of-life recovery. - It was also used to find out the interconnectedness between the socio-economic and policy drivers. - The stock and flow diagram was adopted to model the gross benefits of remanufacturing process. | - The dynamics of cost, revenue and regulatory decisions for recovery of EVBs were studied in the system dynamics model. - The system dynamics model suggested a need for shared understanding among the building blocks of CE including business models, reverse supply chain, policy, product/service use, end-of-life recovery, and product/service design. | No. | France | Vensim |
| (Chaudhary and Vrat, 2020) | - Highlighted the economic, environmental, and social benefits India could achieved by recycling the gold contained in the discarded cell phones. | - The system dynamics model was presented to show the flow of gold from mining / urban mining to the recycling stage. | - system dynamics model was used to demonstrate the system behaviour on social benefits, economic benefits and environmental benefits. | No | India | Stella |
| (Chaudhary and Vrat, 2019) | - Presented the sustainability link of e-waste reverse supply chain in India. - A system dynamics model of circular flow of electronic products from the manufacturing stage to recycling stage was developed. - Seven scenarios were developed to examine the effects of changes of different variables on the system behaviour. | - The system dynamics model was adopted to examine the benefits of increasing the collection capacity, pre-processing capacity, and precious metal extraction capacity in case of a reverse supply chain. | - Increasing the collection capacity, pre-processing capacity, and precious metal extraction capacity showed the increased sustainable benefits. Thus, recommendations were made to increase these variables of the organised sector by building infrastructure. | No | India | Stella |
| (Sinha et al., 2016) | - Examined the possibilities of closing the material flow loop in the global mobile phone product system while addressing the broad sustainability challenges linked to the recovery of materials. - Tried to identify the potential drivers for closing the material flow loops efficiently by better understanding the dynamics of global mobile phone product system (GMPPS). - Proposed a future eco-cycle scenario based on potential drivers and eco-cycle concept and provided suggestions for implementing this scenario in the real-world situation of the GMPPS. - Two circularity indicators were used: loop leakage and loop efficiency, which quantify the degree to which metals are efficiently preserved in the system. | - System dynamics model was adopted from the equation-based model to conduct metal flow analysis of GMPPS in a life cycle perspective to explore an eco-cycle scenario. - The mobile phone flows subsystem was portrayed using the system dynamics diagram notation. - Several subsystems were developed using the system dynamics concept: mobile phone flows subsystem, consumer subsystem (enumerates the consumer behaviour and choice), metal flows subsystem (metal requirement), economic subsystem (costs, price-demand elasticity), etc. | - Loop leakage as well as efficiency were minimized by tuning the following parameters: accessibility to collection pathways, mobile phone use time, gold recovery and mobile phone hibernation. - Parameter settings for eco-cycle scenario were found out. | Yes   - OptQuest optimizer was adopted to optimize both high and low sensitive parameters. | Global study | AnyLogic |

Table S 11. Reasons for developing causal loop diagrams (CLDs), stock and flow diagrams (SFDs), and descriptions of scenarios for system dynamics modelling in circular economy applications – electrical and electronics products sector

| **References** | **Causal loop diagram(s) developed?** | **For which purposes causal loop diagram(s) was/were developed?** | **Stock and flow diagram(s) developed?** | **For which purposes Stock and flow diagram(s) was/were developed?** | **Scenarios developed?** | **What were the scenarios?** |
| --- | --- | --- | --- | --- | --- | --- |
| (Lähdesmäki et al., 2023) | No | N/A | Yes, but not shown in the paper | To account the global material flow dynamics of the lithium flow. | Yes | Sustainable development scenario, and stated policies scenario |
| (Guzzo et al., 2022b) | No | N/A | Yes | To depict the flow of electrical and electronics equipment and their waste. | Yes | Developed 12 scenarios by varying the coverage increase (million inhabitants/year), distribution (inhabitants/collection point) and reward (dimensionless) parameters. |
| (Guzzo et al., 2022a) | No | But the passive interplay between value proposition, creation, delivery was showed. | Yes | To depict the flow of electrical and electronics equipment and their waste. | Yes | Baseline scenario, collection policy, BIAEEE targets, and 17% thresholds. |
| (Guzzo et al., 2021) | Yes | It was integrated in the SFD diagram. | Yes | To represent the flow of materials and technology adoption process. | Yes | Linear economy, baseline, advanced second use, advanced remanufacturing, advanced recycling, shorter lifetime, longer lifetime, and advanced CE. |
| (Llerena-Riascos et al., 2021) | No | N/A | Yes | To describe the reverse logistics process and its associated flow of materials. | Yes | First scenario: benefits of accounting reverse logistics looking for valorisation of the WEEE against the policy of elimination.  Second scenario: effect of processing capacity on the reverse logistic process performance  Third scenario: different replacement frequencies of the mobile phones  Fourth scenario: customers attitude towards repurchases of WEEE. |
| (Salim et al., 2021) | Yes | To study the dynamic hypothesis, information, and material delays of the studied system. | Yes | To model the residential PV market, waste collection and recovery pathways, and policies and regulations. | Yes | Business as usual, market driven growth, conservative development, shared responsibility, and disruptive change. |
| (Alamerew and Brissaud, 2020) | Yes | To model the dynamics of cost, revenue, and strategic and regulatory decisions. | Yes | To model the remanufacturing process of electric vehicle batteries. | Yes | Current condition, and future condition when logistic system was optimized (collection and transport cost was half of the current cost). |
| (Chaudhary and Vrat, 2020) | Yes | To depict the causal relationships between different elements of cell phone reverse supply chain | Yes | To depict the circular flow of gold contained in the cell phones | Yes | Nine (9) scenarios were explored by varying the gold resource exploration, demand for cell phones, collection efficiency of organized sector. |
| (Chaudhary and Vrat, 2019) | Yes | To depict the causality between different factors in the reverse supply chain of e-waste | Yes | To explicate the circular material consumption model and related variables, as well as the sustainable befits of e-waste recycling. | Yes | Seven (7) scenarios were simulated by varying the demand for electronics items, controlled disposal of e-waste, collection capacity and pre-processing capacity. |
| (Sinha et al., 2016) | Yes, but not presented in the article. | To find out the causal relationships between different factors in mobile phone recycling system. | Yes | To represent the flows of material inside the system boundary. | Yes | Business as usual scenario, eco-cycle scenario (maximisation of loop efficiency and minimization of loop leakage) by varying parameters, such as new mobile phone use time, new mobile phone hibernation, accessibility of collection pathways, consumer awareness and gold recovery rate). |

## **Circularity of single material**

Table S 12. Elaborated descriptions of the key points and research questions pertaining to literatures of system dynamics applications in circular economy – circularity of single material.

| **References** | **Key points** | **RQ-1: What are the contexts/necessities of applying system dynamics (circularity special focus)** | **RQ – 2: Which types of decisions were made through system dynamics model?** | **RQ -3: Any other tool(s)/method(s) used in conjunction with system dynamics approach? Why?** | **Location of the study** | **Software used** |
| --- | --- | --- | --- | --- | --- | --- |
| (Ghosh et al., 2023) | - Developed a plastics CE framework in US context considering the critical technologies, policy, and economic constraints for end-of-life management and investment options. - Different circularity metrics were used, and pathways were compared through life cycle assessment (LCA) for environmental impacts. | - System dynamics was used to model the effect of learning-by-doing, where efficiencies of sorting, mechanical recycling, and glycolysis were varied with time. | - Flow of plastics in US economy were realized along with forecasting different circularity indicators. - Glycolysis integrated with expansion of ‘drop-off’ collection access point provided best possible circularity, least global warming and cost over other scenarios. | No | United States | PyLCA for MFA |
| (Saidani et al., 2021) | - Proposed a multi-tool approach to systematically identify, classify, and assess the contribution of influence parameters and action levers to close the loop on the product and key materials. - Combined material flow analysis, fuzzy cognitive mapping, structural analysis, and system dynamics to model and quantify the impact of potential and promising CE strategies. - The connections between key action levers to close the loop on platinum were identified and highlighted, including regulations to limit the number of exports, mandatory recycling and reuse rate, end-users’ behaviours, based on regulatory constraints and financial motivations, and platinum price fluctuation. | - System dynamics was adopted to visualize the interaction among various factors in a product recovery management system, as well as to run 'what-if' simulations (to examine scenarios for various situations). | - Identified the action levers for CE of platinum in catalytic converter. - Due to lack of quantitative data, authors only presented the stock and flow diagram. | Yes   - Fuzzy cognitive mapping was adopted to identify the action levers, drivers, and parameters responsible for closing the loop on platinum. - Functional analysis system technique (FAST) and matrix impact cross-reference multiplication applied to a classification (MICMAC) to list the potential action levers and to select and cluster the key variables, respectively. | European Union | Vensim |
| (Ojha and Vrat, 2020) | - Highlighted the volatility in material prices and other environmental impacts due to sharp depletion of material resources. The depletion was an outcome of linear model of resource consumption based on take-make-dispose approach. - Reprocess and Reuse were used as circular strategy. | - The system dynamics model was developed to understand and analyse long-term policy implications of the circular model on manufacturing growth. - Different scenarios were generated which provided useful insights to facilitate sustainable policy decisions for manufacturing growth. | - Causal loop diagram and thereafter stock and flow diagram were constructed to map the flow of materials inside the system boundary considering the policy constraints. - System dynamics modelling served as an effective tool for policy makers to obtain useful behavioural insights of key variables in the circular consumption of zinc. | No | India | Stella |
| (Pfaff et al., 2018) | - Developed a methodology for analysing material flows in relation to the wider economic system for the special case of copper. - Macroeconomic model and substance flow model were coupled to determine the sectoral copper demand, and the availability of secondary copper. - Highlighted that material flow analysis is a suitable approach for assessing the availability of secondary materials from the point of entry into the economies, their processing, use and storage over product lifespans, and eventually their recycling and disposal. - The proposed model served two purposes: substance flow model showed the state of the current copper cycle in Germany and macroeconomic simulation enabled the analysis of a range of material efficiency scenarios. | - The system dynamics model was used for developing the dynamic material stock and flow model. - The stock-and-flow diagram was coupled with macroeconomic model, which was also implemented in system dynamics. - The macroeconomic model was based on ASTRA model, which has been used in several macroeconomic impact assessment studies. | - Different scenarios were defined (e.g., product lifetime extension, increase in scrap collection rate, increase in availability of secondary copper, reduction of copper intensity of products, fabrication efficiency, absolute demand reduction) and tested from 2013 -2050. - Results were obtained for total domestic end-of-life copper scrap, and total primary copper demand for different scenarios. | Yes.   - ASTRA based macroeconomic model was combined with system dynamics to evaluate the economic aspects of the sector. | Germany | Not mentioned |

Table S 13. Reasons for developing causal loop diagrams (CLDs), stock and flow diagrams (SFDs), and descriptions of scenarios for system dynamics modelling in circular economy applications – circularity of single material

| **References** | **Causal loop diagram(s) developed?** | **For which purposes causal loop diagram(s) was/were developed?** | **Stock and flow diagram(s) developed?** | **For which purposes Stock and flow diagram(s) was/were developed?** | **Scenarios developed?** | **What were the scenarios?** |
| --- | --- | --- | --- | --- | --- | --- |
| (Ghosh et al., 2023) | No | N/A | No | Although SFD was not developed to show the flow of PET resins, Sankey diagrams did the same thing. | Yes | No recycling scenario, mechanical recycling business-as-usual scenario, mechanical recycling with improvement of system parameters, waste to energy, chemical recycling with glycolysis, chemical upcycling via pyrolysis. |
| (Saidani et al., 2021) | Yes | Combined in stocks and flows diagram for indicating how collection-, recovery- and circularity rates were impacted by different parameters. | Yes | Flow of materials of platinum embedded in catalytic converter | Yes | Not mentioned. |
| (Ojha and Vrat, 2020) | Yes | To demonstrate the feedback and causality between the different factors related to linear and circular material consumption with the growth of manufacturing output. | Yes | To represent the circular material consumption (or material flows) in the manufacturing system. | Yes | 12 scenarios were developed by varying the resource explored, demand pattern, recollection efficiency, recollected material recycle ratio. |
| (Pfaff et al., 2018) | No | N/A | Yes | To understand the current stocks in use of copper, EOL material flow and respective recycling efficiencies based on mass balance calculations | Yes | Six (6) scenarios were defined by changing the product lifetime extension, increasing the scrap collection rate, increasing the availability of the secondary copper, reducing the copper intensity of products, fabrication efficiency, and absolute demand reduction. |

N/A: not applicable

## **Manufacturing sector**

Table S 14. Elaborated descriptions of the key points and research questions pertaining to literatures of system dynamics applications in circular economy – manufacturing sector.

| **References** | **Key points** | **RQ-1: What are the contexts/necessities of applying system dynamics (circularity special focus)** | **RQ – 2: Which types of decisions were made through system dynamics model?** | **RQ -3: Any other tool(s)/method(s) used in conjunction with system dynamics approach? Why?** | **Location of the study** | **Software used** |
| --- | --- | --- | --- | --- | --- | --- |
| (Roci et al., 2022) | - Proposed a multi-method simulation approach, comprised of system dynamics, agent-based modelling and simulation (ABMS) and discrete event simulation (DES) for evaluating the economic, environmental, and technical performance of a circular manufacturing system. - The approach was used to track each component in the value chain, and thereafter evaluating their lifecycle cost, lifecycle revenue, and lifecycle environmental impacts. | - Each stakeholder in the value chain was modelled through system dynamics, ABMS, and DES in a chained way. - Furthermore, the complex network was formed due to interconnectedness between the agents, who were interacting with the environment continuously. | - System dynamics was used to model the revenue generated by the firm (for each customer and each month) through stock and flow diagram. | Yes   - ABMS was used to represent the state-space chart. - DES was used to simulate the process flow inside the system boundary. | Over a wide region in Austria | Not mentioned |
| (Franco, 2019) | - Analysed the systemic effects of combining multiple product design and business model strategies for slowing and closing resource loops in CE. - The work aimed at promoting the systematic exploration and quantification of different product design, business models and post-use strategies in the context of manufacturing industry. - Defined different metrics for circular design process, such as disassembly index, recyclability index, functional risk, design for longevity, and compared product service system with purchase-only model. | - System dynamics model was developed to highlight the design and business models for slowing and closing the loops in CES (viz., product lifecycle and demand distribution, product replacement, product reuse, disassembly and recycling, maintenance and repair, product lifetime and discards, and functional risk and green image factor). | - Measuring different indexes for business transition from linear to circular economy system. | No | N/A | Dynaplan ® Smia |
| (Charnley et al., 2019) | - The remanufacturing process was mapped and simulated through DES approach to depict the decision-making process at the shopfloor of a hypothetical remanufacturing facility. - A series of interviews were conducted to find the variability in the condition of the returned products. - The certainty of product quality (CPQ) concept was developed and tested through system dynamics and DES models to understand the effects of CPQ on products awaiting remanufacture, including inspection, cleaning, and disassembly times. - It was found that by reducing the uncertainty of retuned components for remanufacturing can lead to significant cost reduction, and materials and resource savings. | - System dynamics was used to examine the dynamic and long-term behaviour of the system under study, specially remanufacturing. | - The analyses found that there were significant impacts of time spent for remanufacturing depending on the CPQ as well as effect of CPQ on reusable products. | Yes.  DES was used because it allowed the authors to model the system under analysis to be described as a sequence of operations focusing on processes in a system at a medium level of abstraction. | N/A | AnyLogic |

Table S 15. Reasons for developing causal loop diagrams (CLDs), stock and flow diagrams (SFDs), and descriptions of scenarios for system dynamics modelling in circular economy applications – manufacturing sector

| **References** | **Causal loop diagram(s) developed?** | **For which purposes causal loop diagram(s) was/were developed?** | **Stock and flow diagram(s) developed?** | **For which purposes Stock and flow diagram(s) was/were developed?** | **Scenarios developed?** | **What were the scenarios?** |
| --- | --- | --- | --- | --- | --- | --- |
| (Roci et al., 2022) | No | N/A | Yes | To simulate the continuous time financial flow and emissions generated by the customers during the product use phase. | No | N/A |
| (Franco, 2019) | Yes | It was embedded in SFD, to highlight the causality of different indexes and their effects on SFD. | Yes | To represent the flow of materials in different modules: product lifecycle and demand distribution, product replacement, product reuse, maintenance and repair, product lifetime and discard, disassembly, and recycling. | Yes | Different scenarios were generated which were grouped into two categories: short-lifetime products and long-lifetime products by varying different variables, such as potential adopters, contact rate, initial sales per adopter, average residence time, disassembly index, etc. |
| (Charnley et al., 2019) | No | N/A | Yes | To model the remanufacturing process of the fuel cell. | No | N/A |

N/A: not applicable

## **Industrial symbiosis**

Table S 16. Elaborated descriptions of the key points and research questions pertaining to literatures of system dynamics applications in circular economy – industrial symbiosis.

| **References** | **Key points** | **RQ-1: What are the contexts/necessities of applying system dynamics (circularity special focus)** | **RQ – 2: Which types of decisions were made through system dynamics model?** | **RQ -3: Any other tool(s)/method(s) used in conjunction with system dynamics approach? Why?** | **Location of the study** | **Software used** |
| --- | --- | --- | --- | --- | --- | --- |
| (Zhao et al., 2023) | - Emergy analysis was used to quantitatively evaluate the sustainable development of an eco-industrial park through four scenarios – inertia, economic, environmental protection, and science and technology scenarios. - Detailed emergy analysis was carried out considering the causal interrelationships between different social, economic, and environmental sub-systems of the system and other industries. - In stock and flow diagram the flow of energy, materials, and cash were shown along with causality between different system variables. - The system was simulated from 2009-2028. | - To capture the complexity of flows of energy, materials, and cash between different parts of the system. - To perform scenario analysis in a temporal scale. | - The best scenario in terms of sustainable development was selected, which was science and technology scenario. | No. | China | Vensim |
| (Morales et al., 2022) | - Developed a system dynamics model to analyse the sugar-beet value chain in the bio-based industrial symbiosis (BBIS) (from extraction to end-of-life) for understanding the circularity. - It was shown that system dynamics was capable to integrate the MFA in complex structures but was not used to measure circularity. Instead, it was used as a mean to measure multiple variables related to material flow analysis. | - System dynamics helped in systemic understanding of the horizontal and vertical value chain collaborations influencing BBIS. - Different scenarios were analysed for the timeframe of 2018-2027. | - Predictions were carried out for production of sugar, stillage, CO2 emissions, and bioethanol production for all the considered scenarios. | No. | France | Vensim PLE |
| (Dong et al., 2017) | - Used a case study of coal power and cement production in China to analyse the comprehensive effects of CE paradigm through developing a system dynamics model. - Study found that transition to CE can reduce the waste emission and increase economic profit. - Authors also found out that the CE transition can help in reduction of regional pollution, saved mineral resources, improved atmospheric environment, and saving of 14.11 Mt of natural gypsum and 22.67 Mt of coal. | - System dynamics was used to describe the flow of materials inside the system (one coal power plant, two cement plants). It described how the waste of coal power plant became the resource of the cement production plant. - It was also used to build the subsystems for ecological (carbon emission, smoke dust emission, sulphur dioxide mission) and economic effects (sales revenue, reuse of materials and energy, sales revenue of by-products), subsystem of market demand, sub-system of demand of municipal heat. | - Predicted the saving of natural resources, reduction of pollutant and carbon emissions, different economic benefits supply-demand difference of recycled materials, electric power yield, cement production, etc. | No | China | Vensim |

Table S 17. Reasons for developing causal loop diagrams (CLDs), stock and flow diagrams (SFDs), and descriptions of scenarios for system dynamics modelling in circular economy applications – industrial symbiosis.

| **References** | **Causal loop diagram(s) developed?** | **For which purposes causal loop diagram(s) was/were developed?** | **Stock and flow diagram(s) developed?** | **For which purposes Stock and flow diagram(s) was/were developed?** | **Scenarios developed?** | **What were the scenarios?** |
| --- | --- | --- | --- | --- | --- | --- |
| (Zhao et al., 2023) | Yes | To capture the complex relationships between different sub-sectors. | Yes | To capture the flows of energy, materials and cash inside the system | Yes | Inertia, economic, environmental, and science and technology scenario |
| (Morales et al., 2022) | No. | N/A | Yes | Sugar-beet material flows in the bio-based industrial symbiosis of the area of France. | Yes | Baseline scenario, non-viable value chain scenario, outstanding implementation of CE scenario. |
| (Dong et al., 2017) | Yes | To represent how the economy and ecology could be benefitted by different factors in the system, which comprised of one coal power plant, and two cement production plants. | Yes | To depict different flows and stocks (e.g., sales revenue, electric power yield, savings of limestone, clay, gypsum, cost parameters etc.) changing inside the system influenced by different parameters, such as coal consumption, production capacity of the coal plant, etc. | Yes | For economic and ecological effects, different scenarios were generated and analysed, which were grouped into low-risk and high-risk categories. |

## **Miscellaneous sectors**

Table S 18. Elaborated descriptions of the key points and research questions pertaining to literatures of system dynamics applications in circular economy – miscellaneous sector.

| **References** | **Key points** | **RQ-1: What are the contexts/necessities of applying system dynamics (circularity special focus)** | **RQ – 2: Which types of decisions were made through system dynamics model?** | **RQ -3: Any other tool(s)/method(s) used in conjunction with system dynamics approach? Why?** | **Location of the study** | **Software used** |
| --- | --- | --- | --- | --- | --- | --- |
| (Sezer et al., 2024) | - Territorial competitiveness index (TCI) was used to express the sustainable development of Izmir city in Turkey. | - To model the causal dependencies among the various parameters which in turn affected the GDP growth and waste generation rate, and thereby TCI. | - The TCI increased from 2022-2027 due to technological innovations, GDP growth, and implementation of sustainable policies. | No | Turkey | Stella |
| (Kuo et al., 2021) | - To develop a recycle fund system, a system dynamics model was developed and simulated to optimize the recycling funds and subsidies based on decentralized reverse supply chains. - Study suggested that recycling rate was increased by increasing the recycling fund. | - The developed system dynamics model investigated the waste recycling system, including the recycling framework with the stakeholders, and the effects of recycling funds and subsidies on the performance of the recycling activities. - The model tried to achieve the goal of maximisation of recycling rate and minimisation of recycling fund. | - Different scenarios were developed and simulated for the collection and recycling rate based on the extended producer responsibility (EPR) tariff, subsidy cut, recycler subsidy, and collection partner subsidy. - The model also identified the key parameters affecting the generated EPR fund. - Recycling rate, tax-subsidies, and norms had an impact on the policy design. Thus, support from the government was a key part in CE implementation in this work. | No | Indonesia | Vensim PLE |
| (Gao et al., 2020) | - Integrated the system dynamics model into material flow analysis of CE theory to establish a framework that evaluates the comprehensive regional economy. - Different scenarios were generated to provide strategic recommendations for the development of CE in Guangdong province in China. | - The material flow analysis- system dynamics coupling model used system dynamics to connect 13 indexes of material flow analysis to form a regional CE evaluation model. - According to the material flow analysis evaluation and characteristics of CE, the study selected 20 indexes for the comprehensive evaluation of CE in Guangdong province in China. Afterward, these indexes were linked by the system dynamics theory and assigned to four subsystems: socio-economic subsystem, resource consumption subsystem, intensity efficiency subsystem, environmental impact subsystem. - Took 2007 as the base case and simulated the model for 15 years. | - Through scenarios development, biological substance consumption, fossil fuel consumption, solid waste emission, total material input of 10000 RMB of GDP, TMO of 10000 RMB of GDP, building material consumption, industrial exhaust emission were simulated and forecasted from 2007 - 2014. - Results highlighted that building minerals were the main components of domestic material inputs and gas outputs was the main domestic outputs. - Under the circumstances of decrease of birth rate by 2%, the growth rate of tertiary industry increased by 2%. - From policy perspective, authors concluded that govt. interventions were needed to (a) control the birth rate, (b) to develop the tertiary industry for promoting the CE. | Yes  Material flow analysis was used to account for the material flows inside the region, and thereafter 20 indexes were developed and coupled with system dynamics model to predict their values for 15 years period. | China | Vensim |
| (Cheng et al., 2019) | - Developed a Circular-Economy-Effect-and-Policy-Simulation-System-Dynamics (CEEPS- system dynamics) model to evaluate the ecological and economical comprehensive effects of a CE system implemented in a fragility-economic poverty vicious (FPVC) area in China. - In the study, the CE had four benefits - pollution emission elimination, water saving, agricultural waste recycling, as well as energy conservation and emission reduction. | - System dynamics was used to track the flow of material and energy among industries, evaluating the ecological and economic effects, simulating the future development trends, proposing policy improvement areas. - It was also used for scenario development: traditional scenario, CES scenario. | - Improvements in ecological and economic sectors were presented for a period of 16 years. | No | China | Vensim |
| (Asif et al., 2016) | - In their work, they used *'Resource Conservative Manufacturing'* as the synonym for CE. Whereas 'Circular Product Systems' in their context was an approach to implement resource conservative manufacturing or circular economy. - ABMS included the business model and market aspects which provide product demands as the output. - The system dynamics model takes the output of ABMS as input and considered different product design attributes to assess the economic and environmental performance of the circular product systems. - The information about economic and environmental performance was then taken back by the ABMS as an input which eventually influences the demands coming from the ABMS. - Defined physical lifetime, use lifetime, and thereafter different indexes such as estimated CO2, estimated cost in terms of reusability, manufacturability, recyclability, and leakage. - All the indices corresponded to the material mass level. | - The supply chain was modelled using the causal loop diagram. The production rate was triggered by the gap in inventory. - The supply chain and its different components were modelled through system dynamics approach. Initially the system dynamics was used to account the different design attributes and delays. The outputs of the 1st part of system dynamics model were then fed into the second part of the system dynamics model, which accounted the cost and carbon footprint indices. | - The environmental performance, cost based economic performance, and profit based economic performance were estimated for the supply chain which accounted the reuse, recycle, and remanufacturing options of CE. | Yes  ABMS was coupled with system dynamics approach to consider the market information (through population, income of the population, etc) and offer attributes (price to offer, convenience of the offer, etc.) | NA | AnyLogic |

Table S 19. Reasons for developing causal loop diagrams (CLDs), stock and flow diagrams (SFDs), and descriptions of scenarios for system dynamics modelling in circular economy applications – miscellaneous sector.

| **References** | **Causal loop diagram(s) developed?** | **For which purposes causal loop diagram(s) was/were developed?** | **Stock and flow diagram(s) developed?** | **For which purposes Stock and flow diagram(s) was/were developed?** | **Scenarios developed?** | **What were the scenarios?** |
| --- | --- | --- | --- | --- | --- | --- |
| (Sezer et al., 2024) | Yes | How the territorial competitiveness index was causally linked with environment, social, and economic indicators of sustainability. | Yes | The accumulation and growth of annual GDP and waste for the system. | No | N/A |
| (Kuo et al., 2021) | Yes | To study the dynamics of waste generation incorporating the production, costs, and recycling interventions. | Yes | To represent the flow of materials. | Yes | 6 types of scenarios were developed by varying the recycler capacity, EPR tariff, subsidy cut, recycler subsidy, and collection partner subsidy. |
| (Gao et al., 2020) | Yes | To show the causality between different subsystems, such as resource consumption, intensity efficiency, environmental impact, socioeconomic, and thereafter how they impact the regional CE. | Yes | To develop the stock and flow diagram of the whole regional CE system comprised of four subsystems and then to measure different indexes coupled with MFA. | Yes | Nine scenarios were developed by varying the control variables, namely birth rate, growth rate of primary, secondary, and tertiary industry. |
| (Cheng et al., 2019) | No | N/A | Yes | To depict the flows of stocks in dry farming and crop processing industry, biogas and organic fertilizer production industry, energy industry, water saving project. | Yes | Traditional scenario and circular economy scenario by varying different parameters, such as the proportion of faeces in biogas production, growth rate of cultivation, utilization ratio of mulching film recycling, etc. |
| (Asif et al., 2016) | Yes | To depict the causal relationships between different factors of economic, environmental performance, and inventory control mechanism. | Yes | To represent the material flows happening in the supply chain. | Yes | Business as usual scenario, and others by changing the values of the cost, price, and other variables. |

N/A: not applicable

# **Descriptions of the Key Points, Detailed Answers to the Research Questions, and Other Information Related to Applications of Agent-Based Modelling and Simulation Approach in Circular Economy**

## **Bio-based sector**

Table S20. Key literature points pertaining to applications of agent-based modelling and simulation approach to circular economy – bio-based sector

| **References** | **Key points** |
| --- | --- |
| (Farahbakhsh et al., 2023) | - Aim of the study was to add an ecosystem level in the socio-economic aspects of decision-making, and then to make decisions whether do adopt CE or not. - The drivers and barriers of adopting CE strategy in organic waste treatment were explored. - The feasibility of technology adoption was explored in different ways, such as surveys, cost-benefit analysis, economic choice models (feasibility and likelihood of technology adoption). |
| (Voss et al., 2022) | - Chemical recycling was considered as a circular approach for municipal waste management by recirculating the recyclable carbon containing waste as secondary feedstock of chemical production. - The study was aimed at finding the environmental, social, and economic impacts of chemical recycling. - An integrated approach where process-based life-cycle assessment, techno-economic analysis, and social indicators were linked in the framework of an ABMS was developed to examine the sustainability implications of chemical recycling (CR) via gasification of residual MSW in Germany. - Different scenarios were examined, such as business-as-usual (BAU), emission trading adjustment, recycling rate, emission trading adjustment at strong increase in certification price, and recycling rate at strong increase in certification prices. |
| (Tong et al., 2018) | - Authors highlighted that consumer behaviour have a significant role in waste separation process in China, which in turn affects the successful implementation of circular economy. - Authors studied the behavioural change of the households in an effective recycling program through modeling it by integrating ABM and theory of planned behaviour. |

Table S21. Elaborated answers to the research questions for literature pertaining to agent-based modelling and simulation approach to circular economy – bio-based sectors

| **References** | **Who were the agents?** | **RQ-1: Why agent-based modelling and simulation? What are the benefits of using agent-based modelling and simulation (in CE context)?** | **RQ – 2: Which types of decisions were aided by agent-based modelling and simulation?** | **RQ -3: Any other tool/method used in conjunction with agent-based modelling? Why?** |
| --- | --- | --- | --- | --- |
| (Farahbakhsh et al., 2023) | Waste treatment plants. | - The ABMS was adopted to explore the complex interactions between industries, as well as feedback loops. | - Which path to follow (PUFA, SCO, or PHA) under techno-economic constraints. | - No. |
| (Voss et al., 2022) | Administrative areas, primary/secondary waste treatment plants. | - To model the dynamics of sustainability indicators in the context of circularity of chemical recycling of carbon-containing waste of MSW. | - Results showed that chemical recycling contributed to the reduced climate change and addressed challenges of territorial acidification and fossil fuel scarcity. - Different environment impacts (viz., climate change, terrestrial acidification, fossil resource scarcity), economic impact (system costs), and social impact (impact on local employment) were forecasted for a prolonged period. | - No. |
| (Tong et al., 2018) | Households in a specific geographical location. | - The complex interactions of agents and their heterogeneity in terms of socio-economic status, cognitive status, local status, behaviour constraints as well as interactions of the agents with the environment, including: the amount and location of containers, the availability of recycling methods, economic incentives, and provision of information. | - The key factors influencing the households for more recycling practices were identified and improvement scopes were suggested. | - Theory of planned behaviour (TPB) to model the households recycling intentions. |

Table S22. Location of the case-studies, description of the scenarios developed, and software adopted for implementing agent-based models pertaining to circular economy – bio-based sector

| **References** | **Location of the case study** | **Scenarios developed?** | **What were the scenarios?** | **Software adopted for implementing agent-based model** |
| --- | --- | --- | --- | --- |
| (Farahbakhsh et al., 2023) | European Union | Yes | Support of slow growing technology, support of fast-growing technology and strong marker growth and fast-growing technology. | NetLogo |
| (Voss et al., 2022) | Germany | Yes | Baseline scenario, direct residual municipal solid waste (rMSW) incineration was subjected to certificate trading, recycling rate for rMSW and refuse derived fuel (RDF) was introduced, 2^nd^ scenario + strong increase of certificate process and 3^rd^ scenario + strong increase of certificate prices. | MATLAB 2019a |
| (Tong et al., 2018) | China | Yes | Two scenarios were presented: baseline, and new scheme (varying the recycling method, container incentives, collector incentives, and different parameters of theory of planned behaviour). | NetLogo |

## **Electrical and electronics products sector**

Table S 23. Key literature points pertaining to applications of agent-based modelling and simulation approach to circular economy – electrical and electronics products sector

| **References** | **Key points** |
| --- | --- |
| (Walzberg et al., 2022a) | - The developed ABMS intended to understand the how the socio-economic and social status were impacting the end-users’ decisions on EOL management of hard-disk drives apart from shredding. - The data uncertainty was considered by a semi-quantitative approach using a data pedigree matrix. Whereas experts’ judgements were used to assess the data quality and their associated uncertainties. - The simulation was performed for a period of 2020-2050. The quantity of EOL hard-disk drives were modelled through Weibull distribution. |
| (Walzberg et al., 2021) | - Authors adopted the ABMS to integrate the social aspects along with the techno-economic factors for the assessment of the circularity potential of end-of-life management of the photovoltaic solar cells. - Authors performed the sensitivity analyses of the ABM and tried to speed up the model by using a machine learning metamodel. - Five end-of-life strategies were considered, namely: repair, reuse, recycling, landfilling and storage. The model was simulated for the period of 30 years. - The developed ABMS was stochastic in nature as it accounted the variability of some parameters, such as landfill costs. |
| (Luo et al., 2019) | - The recycling system for household waste recovery in China was divided into four categories by the authors, namely: individual recycling, formal recycling stations, producer recycling and online recycling. - Different policy scenarios were explored (six single policy scenario, and four multi-policy scenario), and thereafter optimal policy situation was derived. - The environment of the agent was defined in terms of cost, natural environment, policy, market, resources, technology, and education. |
| (Mashhadi et al., 2019) | - The work investigated the customers’ intention towards leasing mobile phones instead of purchase through a survey analysis. - A regularized logistic regression model was framed to conduct the consumers’ decision model. - The decision model was then adopted to develop the agent-based framework with an intention to articulate the social influences, previous decision, and heterogeneous personal traits towards leasing. |
| (Lieder et al., 2017a) | - The main aim of the study was to provide a decision support system at the intersection of multiple lifecycle design and business models in the circular economy context to identify the effects of cost and CO2 emission. - Thus, authors developed a multi-method simulation approach to quantify the design efforts for circular design options. - Initially design efforts for reuse, recycling, and remanufacturing processes were quantified, and thereafter the associated cost and CO2 emission options for these design efforts were explored. Finally, authors performed an optimization experiment to identify the most cost-effective combinations of efforts. - Authors explored three business model scenarios: buy-back, leasing and pay-per-use. |
| (Lieder et al., 2017b) | - Authors studied the customer behaviour towards accepting new business models in circular economy context. - Authors tried to put forward a quantitative business tool to identify proper marketing and pricing strategy for obtaining best fit demand behaviour of the chosen new business model. - The tool tried to aid the decision-makers to determine the impact of circular business model. - The developed ABMS considered socio-demographic factors of the population and customers' preferences over product attribute price, environmental friendliness, and service-orientation. |

Table S 24. Elaborated answers to the research questions for literature pertaining to agent-based modelling and simulation approach to circular economy – electrical and electronics products sector

| **References** | **Who were the agents?** | **RQ-1: Why agent-based modelling and simulation? What are the benefits of using agent-based modelling and simulation (in CE context)?** | **RQ – 2: Which types of decisions were aided by agent-based modelling and simulation?** | **RQ -3: Any other tool/method used in conjunction with agent-based modelling? Why?** |
| --- | --- | --- | --- | --- |
| (Walzberg et al., 2022a) | End users (service providers, governmental entities, commercial customers), initial service providers, recyclers, and manufacturers. | - To examine the influence of techno-economic and social factors on hard disk drive (HDD) circularity. | - Reuse of the HDD was found to be more useful in terms of environment friendliness than shredding and material recovery. | - TPB was used to model the purchase and end-of-life (EOL) management decisions. |
| (Walzberg et al., 2021) | Photo Voltic (PV) cell owners, PV cell installers, recyclers and manufacturers. | - ABMS was primarily used to represent the flow of materials between different agents across a vast geographical region. - Secondly, it was used to represent the interconnectedness between societal factors and techno-economic factors which affects the overall circularity of PV cells. | - Authors concluded based on the results of the ABMS that exclusions of social factors did not comprehensively represent the material circularity. | - TPB was adopted by the agent (viz., PV owner) to make decisions about whether to comply with CE strategy or not. - Machine learning (ML) metamodel of the ABMS was used to conduct a variance-based sensitivity analysis and explore the ABMS parameter space at a higher speed. ABMS was used to generate both the training and cross-validation data of the ML metamodel, and latter provided expected outputs of the ABMS for a given parameter combinations. |
| (Luo et al., 2019) | Residents, four types of recyclers and government. | - Complex interactions between the agents were modelled through ABMS. - Flows of material from the residents to the recyclers were also modelled by ABMS. | - Different policy scenarios were analysed for best recovery option in China context. - The evolution of the whole resource recovery industry was forecasted, and policy suggestions were made. | - No. |
| (Mashhadi et al., 2019) | Customers involved in cell phone leasing. | - Adopted to model the heterogeneous characteristics of the consumers, such as different socio-demographics and attributes, the effect of social influence and peer pressure, and the ability to trace the agent's decision throughout the simulation. - It was also utilized for modeling the effect of social interactions and previous purchase decision on leasing acceptance attitudes. | - Product-service system was introduced to implement CE by shifting from buying to leasing decision. | - Discrete choice analysis (DCA) a method for consumer choice modeling was adopted to model the consumer preferences and design attributes to predict future market demand. |
| (Lieder et al., 2017a) | Customers | - To describe the heterogeneity in customer behaviour, as well as to account the complex interactions between socio-demographic factors, social network structures, and information diffusion. | - To determine whether the new circular business model (e.g., buy-back or pay-per-use) would be accepted by the customers or not. - Quantification and comparisons of different business models for the following parameters (for 5 years’ time period): market share, customer satisfaction, price and environment friendliness. | - Social connections between agents were modelled through Theory of Homophily, customer preferences by utility functions, and information exchange through Bass diffusion model. |
| (Lieder et al., 2017b) | Each component of the considered system (viz., washing machine). | - To map and track each component of the washing machine, along with their EOL strategies, all their design decisions and other data were required to be transferred. The ABMS was able to track each component and their interactions, and thereafter to combine them for developing the whole product. | - Authors considered three types of design options and they were mapped for four types of EOL strategies. For each of the design scenario, and each of the business model (and thereafter supply chains), the number of customers served, aggregated lifecycle cost, aggregated lifecycle impact was calculated for 15 years period. - Also, material savings for optimized design and linear model were computed to highlight the benefits of CE. | - DES was used to model CE business operations, such as assembly, forward transport, customer use phase as well as reverse transport to account for quality, quantity and timing of the products returns. - Based on the outputs of the ABMS, authors tried to optimize the pay-per-use business model for cost effective outputs. |

Table S 25. Location of the case-studies, description of the scenarios developed, and software adopted for implementing agent-based models pertaining to circular economy – electrical and electronics products sector

| **References** | **Location of the case study** | **Scenarios developed?** | **What were the scenarios?** | **Software adopted for implementing agent-based model** |
| --- | --- | --- | --- | --- |
| (Walzberg et al., 2022a) | United States | Yes | Baseline scenario (assuming all hard disk drivers were shredded, and materials were recovered as per current practice), partial rare earth element (REE) recovery, complete REE recovery, and complete REE recovery including manufacturing waste. | Mesa Python package |
| (Walzberg et al., 2021) | United States | Yes | Landfill ban, high material recovery, lower recycling costs, higher landfill costs, improved lifetime, improved learning effect, reuse warranties, seeding reuse, and baseline | Mesa Python package to define the types of agents, and NetworkX Python to build the social networks of the agents |
| (Luo et al., 2019) | China | Yes | Six single policy scenarios, and four multiple policy scenarios were developed and examined. | NetLogo |
| (Mashhadi et al., 2019) | United States | No | Sensitivity analyses were performed by changing the price policies. | Not mentioned |
| (Lieder et al., 2017a) | Sweden | Yes | Conventional sales and competition, buy-back offer, pay-per-use offer. | AnyLogic |
| (Lieder et al., 2017b) | Sweden | Yes | Different types of design options for multiple scenarios: buy-back, leasing and pay-per-use. | AnyLogic |

## **Industrial symbiosis**

Table S 26. Key literature points pertaining to applications of agent-based modelling and simulation approach to circular economy – industrial symbiosis

| **References** | **Key points** |
| --- | --- |
| (Lange et al., 2021a) | - Authors pointed out that the failures of industrial symbiosis networks were caused by the non-active participations of the agents. - The developed ABMS studied the effects of the behaviour of the agents on the industrial symbiosis network. - The work simulated the implementation of synergies for local waste exchange and compost production. |
| (Lange et al., 2021b) | - Tried to simulate the circular business model prior to implementation. - The ABMS was adopted to simulate the viability effects of two circular business models in different scenarios. - Authors found that most of the industrial symbiosis failed due to early departure of the participants (or agents), possibly because of less supply of waste materials as expected. |
| (Yu et al., 2021) | - The authors addressed the issue of lacked economic incentives in the context of industrial symbiosis network emerged by replacing natural concrete aggregate by recycled concrete aggregate. - The ABMS was implemented to demonstrate the supply-demand and collaboration dynamics of recycled concrete aggregate industrial symbiosis network. The system was integrated with global information system. |
| (Raimbault et al., 2020) | - The goal of the work was to introduce a method for modelling the symbiotic exchanges between the agents from the macro or system level perspective. - The proposed model tried to locate the different actors in different regions, and to cluster them as per their distance and by-products. - All the agents had same goal: minimizing the waste and maximizing the profit. - Authors concluded that geographical properties have an important effect on the macro performance of the symbiotic process. |
| (Fernandez-Mena et al., 2020a) | - Authors simulated alternative scenarios of material flows across a single farming region through developing a ABMS based *‘Flows in Agro-Food Networks’*. - The flow of materials between two agents were dependent on (stochastic in nature): the distance between the agents, the quantity demanded and supplied, the preference coefficient chosen by the simulator for a specific material use. |
| (Fernandez-Mena et al., 2020b) | - Simulated the processing and exchange of fertilizers, food, feed, waste among farms and multiple upstream and downstream partners in small farming region of France. - The material flows/exchanges among these firms were simulated through ABMS. - Authors also highlighted the strong interconnections between the spatial distance of agents, their disposition to exchange, and their preference for specific materials. |
| (Fraccascia and Yazan, 2018) | - Authors investigated the role of information sharing in a successful industrial symbiotic network. - Authors designed an ABM to simulate the advent and procedures of self-organized industrial symbiosis networks in three scenarios – no information sharing platform, a platform where companies shared information about their geographical location and types and quantity of produced and required wastes, and a platform where companies shared information regarding their costs of operation of industrial symbiosis. |
| (Yazan and Fraccascia, 2020) | - The paper proposed a methodology that integrated enterprise input-output modeling with ABMS to assess the sustainability of industrial symbiotic network. - Authors identified multiple key factors that affected the sustainability of the industrial symbiotic network, including resource efficiency, waste reduction and economic benefits. |
| (Fraccascia et al., 2020) | - Authors investigated the impact of increased redundancy on economic and environmental performances of the industrial symbiosis. - Authors referred the optimal redundancy strategy to an optimal number of partners to (from) which waste producers (users) should send (receive) waste to maximize the industrial symbiosis performance. - The agents were characterized by their capacity to produce and consume different types of resources and their willingness to engage in resource exchanges with other actors in the network. The simulation included the presence of some external factors, such as resource availability and technological innovation that affected the behaviour and interactions of the agents. |
| (Fraccascia, 2020) | - Authors investigated the direct network effect for online platforms supporting industrial symbiosis, a strategy to support transition towards CE. - ABMS was designed to simulate the emergence of IS relationships among companies located in a geographical area. |

Table S 27. Elaborated answers to the research questions for literature pertaining to agent-based modelling and simulation approach to circular economy – electrical and electronics products sector

| **References** | **Who were the agents?** | **RQ-1: Why agent-based modelling and simulation? What are the benefits of using agent-based modelling and simulation (in CE context)?** | **RQ – 2: Which types of decisions were aided by agent-based modelling and simulation?** | **RQ -3: Any other tool/method used in conjunction with agent-based modelling? Why?** |
| --- | --- | --- | --- | --- |
| (Lange et al., 2021a) | Waste suppliers, local waste processor, and external waste incinerators. | - Since real world experimentation is impracticable to explore many parameters and actor behaviours, the ABMS was used to model the relationships between the actors in the industrial symbiosis network (ISN). | - How planned behaviour affects the cash flow outcomes of the social agents and robustness of the ISN. | - TPB was adopted to model the agent behaviour in time-dependent bilateral negotiations and synergy evaluation process. |
| (Lange et al., 2021b) | Waste suppliers, waste processors, and waste incinators. | - To find out which circular business model (CBM) and in which combination led to highest survival rate and higher value captured. - Thereafter, proposals were made to improve the CBM design, from company level to network level. | - Two types of scenarios were compared and their efficiencies in terms of survival rate and value captured/lost. | - TPB was adopted to model the behavioural intentions among the waste suppliers and waste processors. |
| (Yu et al., 2021) | Two types of agents in the recycled concrete aggregate (RCA) supply chain: destination agents and vehicle agents. | - To capture the dynamic interactions between the actors as well as with the environment, ABM was adopted. - It was also used as a tool to facilitate the policymaking. | - It was found that ISN exists in the construction industry in implicit manner as RCA treatment requires the collaboration of different actors across substantial spatial and temporal differences. | - The geographic information system (GIS) served as a digital tool which articulated the complex spatial relationships of industrial actors and delivered a visual framework for the conceptualizing understanding, and prescribing decisions regarding emergence of IS agglomerations. |
| (Raimbault et al., 2020) | Enterprise located on a spatial plane, each had some inputs and outputs in terms of needs and waste. | - To model the complex interactions between the agents in a micro-scale symbiotic network. | - Geographical properties are key factor in success of a symbiotic network. - Spatial correlation led to a very different and effective macro performance. - Symbiotic linkages could be optimized to get the maximum benefits from ISNs. | - Multi-objective optimization was adopted to minimize the cost and waste products given that agents were distributed in a different geographical location. |
| (Fernandez-Mena et al., 2020a) | Farms, partner, grain food collector, milk and cheese industry, slaughterhouse, fruit and vegetable industry, wastewater treatment station and fertilizer wholesaler. | - To simulate the flow of materials across the range of components in an agro-food network. - To account the complex interactions between the agents. | - Authors found that a strong connection exists between the spatial distance between agents, their tendency to exchange and preferences for specific materials in small farming region of flows in agro-food network (FAN). | - GIS was adopted to highlight the distances between the agents. |
| (Fernandez-Mena et al., 2020b) | Different firms and their upstream and downstream partners. | - To model the interactions arising from material exchanges in the farming activities by simulating recycling flows and assessing their degree of circularity. - The ABMS was also used to simulate the set of activities performed over a period, such as feed exchanges, livestock production, food processing, food waste and by-products exchanges and bioenergy production. | - Authors found that collective solutions for recycling (viz., organic fertilization and anaerobic digestion) improved the degree of circularity without hampering the food production. - Maximum circularity scenario substantially reduced the GHG emissions. | - Adopted multi-criteria assessment to compare the performances of different scenarios through relative score obtained for key production (viz., crop, livestock, and bioenergy production) and circularity indicators (viz., feed autonomy, bioenergy production, N cycle closing, and GHG mitigation). |
| (Fraccascia and Yazan, 2018) | ISN participators | - To model the spontaneous creation and operations of an ISN in different scenarios. | - The economic and environmental performances of ISNs in different scenarios were measured and compared. | - Physical and monetary flows were modelled through enterprise input-output analysis (EIOA). |
| (Yazan and Fraccascia, 2020) | ISN in China including multiple organizations from steel, chemical and power generation sectors. | - To model and simulate the complex interactions between the agents involved in the ISN. | - Participants of the ISN could be benefited in terms of reduced resource consumption and waste generation and increased economic benefits. | - No. |
| (Fraccascia et al., 2020) | Different actors involved in an ISN. | - ABMS was used to explore the effects of different redundancy strategies on the economic and environmental performance of the companies involved in an ISN. | - Results showed that optimal redundancy strategy was guided by collective influences of waste market dynamicity and transactions costs. | - No. |
| (Fraccascia, 2020) | Waste producers and waste receivers. | - Benefits companies could obtain from online platforms supporting ISNs. - Due to lack of data, ABMS was adopted as it can perform well in that scenario also for decision-making. | - Authors tried to find out what was the minimum usage rate of online platforms to get benefits supporting IS. | - No. |

Table S 28. Location of the case-studies, description of the scenarios developed, and software adopted for implementing agent-based models pertaining to circular economy – electrical and electronics products sector

| **References** | **Location of the case study** | **Scenarios developed?** | **What were the scenarios?** | **Software adopted for implementing agent-based model** |
| --- | --- | --- | --- | --- |
| (Lange et al., 2021a) | Netherland | No | But sensitivity analyses were carried out by varying the theory of planned behaviour ON/OFF, changing moderating variables (e.g., organisational design and context variables) value (note: organisational design variable values were fixed). | Not mentioned (but based on the previous work, it is assumed that author implemented it in NetLogo) |
| (Lange et al., 2021b) | Netherland | Yes | Incinerator payment per month and incinerator payment per tonne of waste | NetLogo |
| (Yu et al., 2021) | Netherland | Yes | Quantity scenarios: waste supplies of demolition sites and up-cycling efficiency were varied.  Cost scenarios: purchasing cost, down-cycling cost, up-cycling costs, combined costs of traditional business model and circular business model were varied. | AnyLogic |
| (Raimbault et al., 2020) | Netherland | No | But authors put forward the changes in circularity level by varying the level of clustering of agents as well as the changes to relative costs with respect to total waste generation. | NetLogo |
| (Fernandez-Mena et al., 2020a) | France | Yes | Business as usual, best management practices at the farm scale, promotion of the material exchanges between farms and their partner network, implementation of biogas production plants at maximum potential, a complete crop-livestock symbiosis, maximum circularity, livestock reduced to half of the population, maximum circularity along with livestock reduced to half of its population. | GIS Agent-based Modeling Architecture (GAMA) platform |
| (Fernandez-Mena et al., 2020b) | France | Yes | A basic scenario was analysed which tried to maximize the material exchange and bioenergy production. | GIS Agent-based Modeling Architecture (GAMA) platform |
| (Fraccascia and Yazan, 2018) | N/A | Yes | No information sharing, platform sharing non-sensitive information and platform sharing sensitive information. | Not mentioned |
| (Yazan and Fraccascia, 2020) | N/A | Yes | Different scenarios were developed: base-case, and others by varying the parameters, such as waste discharge cost, input purchase cost, additional cost, etc. | Not mentioned |
| (Fraccascia et al., 2020) | N/A | Yes | Different scenarios were examined by varying the redundancy strategy, waste market dynamicity, and transaction costs of the firms. | Not mentioned |
| (Fraccascia, 2020) | N/A | Yes | Different scenarios were generated by different platform usage rate. | Not mentioned |

## **Miscellaneous sectors**

Table S 29. Key literature points pertaining to applications of agent-based modelling and simulation approach to circular economy – miscellaneous sectors

| **References** | **Key points** |
| --- | --- |
| (Walzberg et al., 2022b) | - A spatially resolved ABMS combined with machine learning metamodel showed that including the behavioural factors was important in designing effective policies. The outputs generated by the ABM were fed to the machine learning model. - Wind power industry stakeholders’ behavioural decisions can impact the wind blade circularity, along with the transportation distance. - Theory of planned behaviour (TPB) was adopted to represent agent’s decisions, which were stochastic in nature. The model was simulated for a period of 30 years. |
| (Fani et al., 2022) | - Authors tried to develop a business model of the fashion renting process considering both customer and company’s viewpoint. - ABMS facilitated the modeling of customer’s behaviour and interactions. - Discrete event simulation was used to model the process flow of fashion renting process. - Authors identified the barriers and motivators of the fashion renting process. |

Table S 30. Elaborated answers to the research questions for literature pertaining to agent-based modelling and simulation approach to circular economy – miscellaneous sectors

| **References** | **Who were the agents?** | **RQ-1: Why agent-based modelling and simulation? What are the benefits of using agent-based modelling and simulation (in CE context)?** | **RQ – 2: Which types of decisions were aided by agent-based modelling and simulation?** | **RQ -3: Any other tool/method used in conjunction with agent-based modelling? Why?** |
| --- | --- | --- | --- | --- |
| (Walzberg et al., 2022b) | Wind plant owners, wind plant developers, recyclers, original equipment manufacturers, landfills, and regulators. | - To represent the heterogeneity of the stakeholders in a spatial case. - The ABMS coupled with TPB forecasted the recycling of wind turbine blades as per different pathways. | - How the behavioural patterns could affect the end-of-life pathways of wind turbine blades. | - TPB was adopted to represent agents’ behaviour and its effects on the neighbouring agents. |
| (Fani et al., 2022) | Customer behaviour | - The customer behaviour and the (experience during the service when the customer is in the store for renting) and the experience when the customer leaves the store. | - The attitude of the customer to participate in the fashion renting process and thereby the success of the business model. | - DES was used to model the fashion renting process. |

Table S 31. Location of the case-studies, description of the scenarios developed, and software adopted for implementing agent-based models pertaining to circular economy – miscellaneous sectors

| **References** | **Location of the case study** | **Scenarios developed?** | **What were the scenarios?** | **Software adopted for implementing agent-based model** |
| --- | --- | --- | --- | --- |
| (Walzberg et al., 2022b) | United States | Yes | Baseline scenario, no transportation cost, summation of no transportation costs + perceived barrier in adopting the behaviour was zero, sum of no transportation costs + subjective norm + + perceived barrier in adopting the behaviour was zero.  Other scenarios were early adoption scenario, adopting the thermoplastic blade design. | Mesa Python package |
| (Fani et al., 2022) | No specific region | No | N/A | AnyLogic |

# **Funding**

This research was funded as part of the UKRI Interdisciplinary Circular Economy Centre for Mineral-based Construction Materials (ICEC-MCM), by the Engineering and Physical Sciences Research Council (EPSRC) - Grant reference: EP/V011820/1.

# **CRediT Author Statement**

**Soumava Boral:** Conceptualisation, Methodology, Writing – Original draft preparation, Formal analysis, Data Curation, Visualization; **Leon Black:** Conceptualisation, Writing – Review and Editing, Visualisation, Supervision, Funding acquisition; **Costas Velis:** Conceptualisation, Methodology, Writing – Review and Editing, Supervision, Project administration, Funding acquisition.

# **Acknowledgements**

We acknowledge the support of UKRI Interdisciplinary Circular Economy Centre for Mineral-based Construction Materials (EP/V011820/1). We also acknowledge Mr. Ed Cook from University of Leeds for his constructive suggestions while drafting this article.

**References**

Abbasi, I.A., Shamim, A., Shad, M.K., Ashari, H., Yusuf, I., 2024. Circular economy-based integrated farming system for indigenous chicken: Fostering food security and sustainability. J. Clean. Prod. 436, 140368.

Alamerew, Y.A., Brissaud, D., 2020. Modelling reverse supply chain through system dynamics for realizing the transition towards the circular economy: A case study on electric vehicle batteries. J. Clean. Prod. 254, 120025.

Arnold, R.D., Wade, J.P., 2015. A definition of systems thinking: A systems approach. Procedia Comput. Sci. 44, 669–678.

Asif, F.M., Lieder, M., Rashid, A., 2016. Multi-method simulation based tool to evaluate economic and environmental performance of circular product systems. J. Clean. Prod. 139, 1261–1281.

Bandini, S., Manzoni, S., Vizzari, G., 2009. Agent based modeling and simulation: an informatics perspective. J. Artif. Soc. Soc. Simul. 12, 4.

Barnabè, F., Nazir, S., 2022. Conceptualizing and enabling circular economy through integrated thinking. Corp. Soc. Responsib. Environ. Manag. 29, 448–468.

Bocken, N.M., Olivetti, E.A., Cullen, J.M., Potting, J., Lifset, R., 2017. Taking the circularity to the next level: a special issue on the circular economy. J. Ind. Ecol.

Charnley, F., Tiwari, D., Hutabarat, W., Moreno, M., Okorie, O., Tiwari, A., 2019. Simulation to enable a data-driven circular economy. Sustainability 11, 3379.

Chaudhary, K., Vrat, P., 2020. Circular economy model of gold recovery from cell phones using system dynamics approach: a case study of India. Environ. Dev. Sustain. 22, 173–200.

Chaudhary, K., Vrat, P., 2019. Sustainable benefits of electronic waste recycling in India: a system dynamics analysis. Int. J. Product. Qual. Manag. 27, 369–393.

Cheng, H., Dong, S., Li, F., Yang, Y., Li, Y., Li, Z., 2019. A circular economy system for breaking the development dilemma of ‘ecological Fragility–Economic poverty’vicious circle: A CEEPS-SD analysis. J. Clean. Prod. 212, 381–392.

de Carvalho Freitas, E.S., Xavier, L.H., Oliveira, L.B., Guarieiro, L.L.N., 2022. System dynamics applied to second generation biofuel in Brazil: A circular economy approach. Sustain. Energy Technol. Assess. 52, 102288.

Demartini, M., Tonelli, F., Govindan, K., 2022. An investigation into modelling approaches for industrial symbiosis: A literature review and research agenda. Clean. Logist. Supply Chain 3, 100020.

Dong, S., Wang, Z., Li, Y., Li, F., Li, Z., Chen, F., Cheng, H., 2017. Assessment of comprehensive effects and optimization of a circular economy system of coal power and cement in Kongtong District, Pingliang City, Gansu Province, China. Sustainability 9, 787.

Eissa, R., El-adaway, I.H., 2024. Circular Economy Policies for Decarbonization of US Commercial Building Stocks: Data Integration and System Dynamics Coflow Modeling Approach. J. Manag. Eng. 40, 04024003.

El Wali, M., Golroudbary, S.R., Kraslawski, A., 2021. Circular economy for phosphorus supply chain and its impact on social sustainable development goals. Sci. Total Environ. 777, 146060.

Ellen MacArthur Foundation, 2019. Systems and the circular economy – deep dive. URL https://ellenmacarthurfoundation.org/systems-and-the-circular-economy-deep-dive (accessed 5.5.23).

Fani, V., Pirola, F., Bindi, B., Bandinelli, R., Pezzotta, G., 2022. Design Product-Service Systems by Using a Hybrid Approach: The Fashion Renting Business Model. Sustainability 14, 5207.

Farahbakhsh, S., Snellinx, S., Mertens, A., Belderbos, E., Bourgeois, L., Van Meensel, J., 2023. What’s stopping the waste-treatment industry from adopting emerging circular technologies? An agent-based model revealing drivers and barriers. Resour. Conserv. Recycl. 190, 106792.

Fernandez-Mena, H., Gaudou, B., Pellerin, S., MacDonald, G.K., Nesme, T., 2020a. Flows in Agro-food Networks (FAN): An agent-based model to simulate local agricultural material flows. Agric. Syst. 180, 102718.

Fernandez-Mena, H., MacDonald, G.K., Pellerin, S., Nesme, T., 2020b. Co-benefits and trade-offs from agro-food system redesign for circularity: A case study with the FAN agent-based model. Front. Sustain. Food Syst. 4, 41.

Fiksel, J., Bruins, R., Gatchett, A., Gilliland, A., Ten Brink, M., 2014. The triple value model: a systems approach to sustainable solutions. Clean Technol. Environ. Policy 16, 691–702.

Fraccascia, L., 2020. Quantifying the direct network effect for online platforms supporting industrial symbiosis: an agent-based simulation study. Ecol. Econ. 170, 106587.

Fraccascia, L., Yazan, D.M., 2018. The role of online information-sharing platforms on the performance of industrial symbiosis networks. Resour. Conserv. Recycl. 136, 473–485.

Fraccascia, L., Yazan, D.M., Albino, V., Zijm, H., 2020. The role of redundancy in industrial symbiotic business development: A theoretical framework explored by agent-based simulation. Int. J. Prod. Econ. 221, 107471.

Franco, M.A., 2019. A system dynamics approach to product design and business model strategies for the circular economy. J. Clean. Prod. 241, 118327.

Gandhi, A.B., Lad, V., Patel, DJ, Patel, DA, 2024. Feasibility Study of Recycled Paver Block for Circular Economics Using System Dynamics Model. J. Leg. Aff. Dispute Resolut. Eng. Constr. 16, 04523063.

Gao, Chengkang, Gao, Chengbo, Song, K., Fang, K., 2020. Pathways towards regional circular economy evaluated using material flow analysis and system dynamics. Resour. Conserv. Recycl. 154, 104527.

Ghosh, T., Avery, G., Bhatt, A., Uekert, T., Walzberg, J., Carpenter, A., 2023. Towards a circular economy for PET bottle resin using a system dynamics inspired material flow model. J. Clean. Prod. 383, 135208.

Ghufran, M., Khan, K.I.A., Ullah, F., Nasir, A.R., Al Alahmadi, A.A., Alzaed, A.N., Alwetaishi, M., 2022. Circular Economy in the Construction Industry: A Step towards Sustainable Development. Buildings 12, 1004.

Guzzo, D., Pigosso, D., Videira, N., Mascarenhas, J., 2022a. A system dynamics-based framework for examining Circular Economy transitions. J. Clean. Prod. 333, 129933.

Guzzo, D., Rodrigues, V.P., Mascarenhas, J., 2021. A systems representation of the Circular Economy: Transition scenarios in the electrical and electronic equipment (EEE) industry. Technol. Forecast. Soc. Change 163, 120414.

Guzzo, D., Rodrigues, V.P., Pigosso, D.C., Mascarenhas, J., 2022b. Analysis of national policies for Circular Economy transitions: Modelling and simulating the Brazilian industrial agreement for electrical and electronic equipment. Waste Manag. 138, 59–74.

Kliem, D., Scheidegger, A., Kopainsky, B., 2021. Closing the mineral construction material cycle–An endogenous perspective on barriers in transition. Resour. Conserv. Recycl. 175, 105859.

Kopainsky, B., Alessi, S.M., Davidsen, P.I., 2011. Measuring knowledge acquisition in dynamic decision making tasks, in: The 29th International Conference of the System Dynamics Society. System Dynamics Society Albany, NY, pp. 1–31.

Kuo, T.-C., Hsu, N.-Y., Wattimena, R., Hong, I.-H., Chao, C.-J., Herlianto, J., 2021. Toward a circular economy: A system dynamic model of recycling framework for aseptic paper packaging waste in Indonesia. J. Clean. Prod. 301, 126901.

Lähdesmäki, S., Hanski, J., Huttunen-Saarivirta, E., 2023. Insights into circular economy potential of lithium by system dynamic modelling of material streams. Batter. Supercaps 6, e202300231.

Lange, K.P., Korevaar, G., Nikolic, I., Herder, P., 2021a. Actor behaviour and robustness of industrial symbiosis networks: an agent-based modelling approach. J. Artif. Soc. Soc. Simul. 24.

Lange, K.P., Korevaar, G., Oskam, I.F., Nikolic, I., Herder, P.M., 2021b. Agent-based modelling and simulation for circular business model experimentation. Resour. Conserv. Recycl. Adv. 12, 200055.

Lieder, M., Asif, F.M., Rashid, A., 2017a. Towards Circular Economy implementation: an agent-based simulation approach for business model changes. Auton. Agents Multi-Agent Syst. 31, 1377–1402.

Lieder, M., Asif, F.M., Rashid, A., Mihelič, A., Kotnik, S., 2017b. Towards circular economy implementation in manufacturing systems using a multi-method simulation approach to link design and business strategy. Int. J. Adv. Manuf. Technol. 93, 1953–1970.

Lieder, M., Rashid, A., 2016. Towards circular economy implementation: a comprehensive review in context of manufacturing industry. J. Clean. Prod. 115, 36–51.

Llerena-Riascos, C., Jaén, S., Montoya-Torres, J.R., Villegas, J.G., 2021. An Optimization-Based System Dynamics Simulation for Sustainable Policy Design in WEEE Management Systems. Sustainability 13, 11377.

Luo, M., Song, X., Hu, S., Chen, D., 2019. Towards the sustainable development of waste household appliance recovery systems in China: an agent-based modeling approach. J. Clean. Prod. 220, 431–444.

Mashhadi, A.R., Vedantam, A., Behdad, S., 2019. Investigation of consumer’s acceptance of product-service-systems: A case study of cell phone leasing. Resour. Conserv. Recycl. 143, 36–44.

Morales, M.E., Lhuillery, S., Ghobakhloo, M., 2022. Circularity Effect in the Viability of Bio-Based Industrial Symbiosis: Tackling extraordinary events in value chains. J. Clean. Prod. 348, 131387.

Mostert, C., Weber, C., Bringezu, S., 2022. Modelling and Simulation of Building Material Flows: Assessing the Potential for Concrete Recycling in the German Construction Sector. Recycling 7, 13.

Nilsson, F., Darley, V., 2006. On complex adaptive systems and agent-based modelling for improving decision-making in manufacturing and logistics settings: Experiences from a packaging company. Int. J. Oper. Prod. Manag. 26, 1351–1373.

Ojha, R., Vrat, P., 2020. Circular material resource consumption for sustainability of manufacturing growth: a System Dynamics analysis. Int. J. Manuf. Technol. Manag. 34, 349–375.

Parsa, A., Van De Wiel, M., Schmutz, U., Fried, J., Black, D., Roderick, I., 2023. Challenging the food waste hierarchy. J. Environ. Manage. 344, 118554.

Parsa, A., Van De Wiel, M., Schmutz, U., Taylor, I., Fried, J., 2024. Balancing people, planet, and profit in urban food waste management. Sustain. Prod. Consum.

Pfaff, M., Glöser-Chahoud, S., Chrubasik, L., Walz, R., 2018. Resource efficiency in the German copper cycle: Analysis of stock and flow dynamics resulting from different efficiency measures. Resour. Conserv. Recycl. 139, 205–218.

Railsback, S.F., Grimm, V., 2019. Agent-based and individual-based modeling: a practical introduction. Princeton university press.

Raimbault, J., Broere, J., Somveille, M., Serna, J.M., Strombom, E., Moore, C., Zhu, B., Sugar, L., 2020. A spatial agent based model for simulating and optimizing networked eco-industrial systems. Resour. Conserv. Recycl. 155, 104538.

Ranjbari, M., Esfandabadi, Z.S., Siebers, P.-O., Pisano, P., Quatraro, F., 2024. Digitally enabled food sharing platforms towards effective waste management in a circular economy: A system dynamics simulation model. Technovation 130, 102939.

Reike, D., Vermeulen, W.J., Witjes, S., 2018. The circular economy: new or refurbished as CE 3.0?—exploring controversies in the conceptualization of the circular economy through a focus on history and resource value retention options. Resour. Conserv. Recycl. 135, 246–264.

Richmond, B., 1994. Systems thinking/system dynamics: Let’s just get on with it. Syst. Dyn. Rev. 10, 135–157.

Roci, M., Salehi, N., Amir, S., Shoaib-ul-Hasan, S., Asif, F.M., Mihelič, A., Rashid, A., 2022. Towards circular manufacturing systems implementation: a complex adaptive systems perspective using modelling and simulation as a quantitative analysis tool. Sustain. Prod. Consum. 31, 97–112.

Saidani, M., Yannou, B., Leroy, Y., Cluzel, F., Kim, H., 2021. Multi-tool methodology to evaluate action levers to close the loop on critical materials–Application to precious metals used in catalytic converters. Sustain. Prod. Consum. 26, 999–1010.

Salim, H.K., Stewart, R.A., Sahin, O., Dudley, M., 2021. Dynamic modelling of Australian rooftop solar photovoltaic product stewardship transition. Waste Manag. 127, 18–29.

Sezer, M.D., Kazancoglu, Y., Mangla, S.K., 2024. Analysing of the territorial competitiveness index in Izmir through dynamic model. Resour. Policy 88, 104431.

Sinha, R., Laurenti, R., Singh, J., Malmström, M.E., Frostell, B., 2016. Identifying ways of closing the metal flow loop in the global mobile phone product system: A system dynamics modeling approach. Resour. Conserv. Recycl. 113, 65–76.

Sterman, J.D., 2000. Business Dynamics: Systems Thinking and Modeling for a Complex World. McGraw-Hill Higher Education, United States of America.

Sweeney, L.B., Sterman, J.D., 2000. Bathtub dynamics: initial results of a systems thinking inventory. Syst. Dyn. Rev. J. Syst. Dyn. Soc. 16, 249–286.

The British Standards Institution, 2024. Circular economy - Vocabulary, principles and guidance for implementation.

Tong, X., Nikolic, I., Dijkhuizen, B., van den Hoven, M., Minderhoud, M., Wäckerlin, N., Wang, T., Tao, D., 2018. Behaviour change in post-consumer recycling: applying agent-based modelling in social experiment. J. Clean. Prod. 187, 1006–1013.

Tricco, A.C., Lillie, E., Zarin, W., O’Brien, K.K., Colquhoun, H., Levac, D., Moher, D., Peters, M.D., Horsley, T., Weeks, L., others, 2018. PRISMA extension for scoping reviews (PRISMA-ScR): checklist and explanation. Ann. Intern. Med. 169, 467–473.

Valencia, A., Zhang, W., Chang, N.-B., 2022. Sustainability transitions of urban food-energy-water-waste infrastructure: A living laboratory approach for circular economy. Resour. Conserv. Recycl. 177, 105991.

Viruega Sevilla, D., Francisco López, A., Bello Bugallo, P.M., 2022. The Role of a Hazardous Waste Intermediate Management Plant in the Circularity of Products. Sustainability 14, 1241.

Voss, R., Lee, R.P., Fröhling, M., 2022. A consequential approach to life cycle sustainability assessment with an agent-based model to determine the potential contribution of chemical recycling to UN Sustainable Development Goals. J. Ind. Ecol.

Walzberg, J., Burton, R., Zhao, F., Frost, K., Muller, S., Carpenter, A., Heath, G., 2022a. An investigation of hard-disk drive circularity accounting for socio-technical dynamics and data uncertainty. Resour. Conserv. Recycl. 178, 106102.

Walzberg, J., Carpenter, A., Heath, G.A., 2021. Role of the social factors in success of solar photovoltaic reuse and recycle programmes. Nat. Energy 6, 913–924.

Walzberg, J., Cooperman, A., Watts, L., Eberle, A.L., Carpenter, A., Heath, G.A., 2022b. Regional representation of wind stakeholders’ end-of-life behaviors and their impact on wind blade circularity. Iscience 25, 104734.

Xing, L., Lin, T., Hu, Y., Lin, M., Liu, Y., Zhang, G., Ye, H., Xue, X., 2023. Reducing food-system nitrogen input and emission through circular agriculture in montane and coastal regions. Resour. Conserv. Recycl. 188, 106726.

Xu, J., Li, X., Wu, D.D., 2009. Optimizing circular economy planning and risk analysis using system dynamics. Hum. Ecol. Risk Assess. 15, 316–331.

Yazan, D.M., Fraccascia, L., 2020. Sustainable operations of industrial symbiosis: an enterprise input-output model integrated by agent-based simulation. Int. J. Prod. Res. 58, 392–414.

Yu, Y., Yazan, D.M., Bhochhibhoya, S., Volker, L., 2021. Towards Circular Economy through Industrial Symbiosis in the Dutch construction industry: A case of recycled concrete aggregates. J. Clean. Prod. 293, 126083.

Zhao, Y., Yu, M., Xiang, Y., Chang, C., 2023. An approach to stimulate the sustainability of an eco-industrial park using coupled emergy and system dynamics. Environ. Dev. Sustain. 25, 11531–11556.

1. A set of graphs and other descriptive data showing the development of the problem over time. [↑](#footnote-ref-2)
2. The hypothesis is dynamic because it must provide an explanation of the dynamics characterizing the problem in terms of underlying feedback and stock and flow structures of the system. It is a hypothesis because it is always provisional, subject to revision or abandonment as you learn from the modelling process and form the real world. [↑](#footnote-ref-3)
3. ‘Endogenous’ means ‘arising from within’, which implies that the dynamics of the problem is created by the interactions between the variables or agents. [↑](#footnote-ref-4)
4. It summarises the scope of the model by listing which key variables are included endogenously, which are exogenous, and which are excluded from the model. [↑](#footnote-ref-5)
5. Subsystem diagrams show the overall architecture of the model. Each major subsystem is shown along the flows of the materials, money, goods, etc. coupling the subsystem to one another. [↑](#footnote-ref-6)
6. Earlier two diagrams don’t show how the variables are connected in a system. Causal loop diagrams (CLD) are simply maps showing the cause-and-effect relationships of the variables. [↑](#footnote-ref-7)
7. CLD emphasises the feedback structure of a system. Whereas stock and flow diagrams (SFD) focus on their underlying physical structure. It might be the accumulation of material, money, information, etc. [↑](#footnote-ref-8)
